# Antimicrobial potential of toothpaste formulated from extracts of *Syzygium aromaticum*, *Dennettia tripetala* and *Jatropha curcas* latex against some oral pathogenic microorganisms

**DOI:** 10.1186/s13568-019-0744-2

**Published:** 2019-02-04

**Authors:** Olugbenga Oludayo Oluwasina, Ifunanya Vivian Ezenwosu, Clement Olusola Ogidi, Victor Olusegun Oyetayo

**Affiliations:** 1 Department of Chemistry, The Federal University of Technology, PMB 704, Akure, Nigeria; 2 Department of Microbiology, The Federal University of Technology, PMB 704, Akure, Nigeria; 3 Biotechnology Unit, Department of Biological Sciences, Kings University, PMB 555, Odeomu, Nigeria

**Keywords:** Herbal toothpaste, Dental caries, Medicinal plants, Bioactive compounds

## Abstract

The need for a broad spectrum antimicrobial mouthwash is highly desirable to reduce, control and prevent various types of dental diseases. Hence, research into the production of herbal toothpaste to suppress the incidence of dental diseases is pertinent. The present study formulated herbal toothpastes from edible and medicinal plants namely; *Syzygium aromaticum*, *Dennettia tripetala*, and *Jatropha curcas* latex. The antimicrobial activity of the toothpastes was assessed against some oral pathogenic microorganisms using agar well diffusion. Phytochemical analysis of *S. aromaticum* and *D. tripetala* revealed the presence of phenols, flavonoids, alkaloid, and saponins. The major constituent of the plants from gas chromatography mass spectrometer (GC/MS) analysis are eugenol (83.58%), caryophyllene (4.35%) and phenol, 2-methoxy-4-(2-propenyl)-, acetate (12.07%) from *S. aromaticum,* while *D. tripetala* had glutaric acid (57.57%), eugenol (2.9%), caryophyllene (1.12%), and 1,6,10-dodecatrien-3-ol, 3,7,11-trimethyl-,(E)- (3.36%). The pH and specific gravity of the formulated toothpastes respectively ranged from 8.57 to 9.67 and 1.08 to 1.10, while the pH and specific gravity of commercial toothpastes were from 5.39 to 8.55 and 0.97 to 1. 11 respectively. The formulated toothpastes have better and significant (P < 0.05) antimicrobial effect when compared to commercial toothpastes. The zones of inhibition of formulated toothpastes against the tested microorganisms ranged from 4.0 to 18.30 mm, while MIC ranged from 2.5 to 20.0 mg/mL. The formulated toothpastes exhibited potent antimicrobial property against the tested pathogens as a result of bioactive compounds in them. Hence, these biomolecules can be extracted for the production of safe and effective herbal-based toothpaste.

**Electronic supplementary material:**

The online version of this article (10.1186/s13568-019-0744-2) contains supplementary material, which is available to authorized users.

## Introduction

Dental caries is becoming a major concern to the health sector in the world. This is because dental plagues are causing different oral diseases and dental caries. Dental caries has caused the destruction of the superficial dental structure, wearing of the enamel and removal of the tooth by releasing acid due to metabolic activities of cariogenic bacteria on the remnant food in the mouth (Botelho et al. 2007). The tooth is an important part of the body; its unhealthy could result in digestive problem and malnutrition. Therefore, there is a need to reduce the activities of cariogenic microorganisms in order to prevent certain diseases associated with dental caries (Galati et al. 1999). Microbes such as *Streptococcus mutans, Staphylococcus aureus, Streptococcus mitis* and *Candida albicans* have been implicated in dental diseases (Hamada and Slade 1980; Loesche 1986; Bhattacharya et al. 2003; Akhtar and Bhakuni 2004).

The advent of modern technology has brought in branded toothpastes and mouthwashes with the incorporation of synthetic antimicrobial agents such as chlorhexidine, triclosan, and fluoride (Zauri-Arite et al. 2001; Maripandi et al. 2011). The use those pastes have been very effective but with some drawbacks. Fluoridated toothpaste, apart from not being recommended for children below 6 years of age, it cause pigment of teeth and weakening of enamel, while chlorhexidine, a chlorophenyl bisbiguanide causes pigmentation of dental, mouth and tongue environment with altered sense of taste, irritation and oral dryness, scaling of gingival and negative systemic effects in ingestion (Parwani et al. 2013; Jain and Ranjan 2014; Ghelichli 2014). Although, much is not yet known about triclosan with respect to it negative oral health effect, nonetheless, it does not bind well to the oral site due to its strong positive charge (Peter 1999).

Though, the modern chemical based toothpaste and mouthwash have been effective in combating cariogenic microbes. However, the major challenging issue is the resistance of these cariogenic microorganisms to some commonly used antibiotics such as penicillin, chloramphenicol, clindamycin, ampicillin and other antimicrobial chemicals (Jarvinen et al. 1993; Bhattacharya et al. 2003).

These drawbacks are the driving forces that cause the need for the formulation of toothpaste from medicinal and edible plants containing natural antimicrobial agents. Nature is endowed with herbs for human use but the world out of a desire for modern development has overlooked this potent provision of the ‘herbs’. Since time immemorial, plant materials have been traditionally used for tooth wash, as chewing stick, latex or exudate or powder mouth to maintain dental health, cure dental disease and to increase oral hygiene. The use of chewing stick is still being practiced in some part of the world such as Africa, South Asia, isolated area in America and Southern United State, owing to some unique characteristics such as foaminess (presence of saponins), hardness (woody nature of the chewing stick) and bitterness (secondary metabolite like saponin and alkaloids) (Kolapo et al. 2009).

The medicinal plants such as *Zanthoxylum americanum*, *Acacia farnesiana*, *Gymnosporia spinosa, Symphytum officinale* (Comfrey), *Garcinia manni, Masularia accuminata, terminalia glaucescens, Anogeissus leiocarpus, Pseudocedrela kotschyi, Xanthoxyllum gilletti, Prosopis africana* and *Azadiracta indica* have displayed antimicrobial activities against some microorganisms responsible for dental caries (Akande and Hayashi 1998; Agboola 2005; Barnes et al. 2007; Khare 2007). Plant materials could be referred to as natural antibiotic reservoir and the development of resistance to various antibiotics has rekindled interest into the medicinal importance of plant. Thus, plant materials need to be investigated for a possible solution to the resistance of cariogenic microbes.

*Syzygium aromaticum* (L.) commonly called clove is from the family *Myrtaceae*. It is an aromatic spice, cultivated in India, Madagascar, Sri Lanka, Indonesia, the south of China and some part of Africa. It is used in preparing Nigerian pepper soup with meats and fishes. Clove oil is medicinal and mostly used for flavouring pastry, special sauces, and condiments. The common uses of clove have been attributed to some of its biological activities such as antibacterial, antifungal, insecticidal and antioxidant properties (Lee and Shibamoto 2001; Huang et al. 2002; Velluti et al. 2003).

*Dennettia tripetala* (pepper fruit) is of *Annonaceae* plant family and could be found in Western Cameroons, Ivory Coast and Nigeria (Southern, Eastern, and Western parts) (Hutchinson and Dalziel 1954; Okiy 1960; Chandraseharen 1994; Etukudo 2000). The whole part of the plant has characteristics like pungent spicy and pepperish taste, which make suitable for spices and condiments (Oyemitan 2006). Its mature fruit, which is the main edible part could be chewed as a fresh green, freshly ripened red, black dry fruit and dry de-hulled seed (Achinewhu et al. 1995). The medicinal property of the plant has been linked to the presence of saponins, flavonoids, tannins, alkaloids, terpenoids, and cyanogenic glycosides (Lewis and Ausubel 2006; Nwaogu et al. 2007). These phytochemicals have contributed to their antimicrobial, antihistaminic and coronary activities (Kumar et al. 2010).

*Jatropha curcas* L. belongs to the family of *Euphorbiaceae*. It is a plant that has its origin in Central America but could be found in many other parts of the world such as tropics and sub-tropics Africa and Asia (Gubitz et al. 1999; Martinez-Herrera et al. 2006; Janick and Paull 2008; Kumar and Sharma 2008). It has been used from time immemorial as chewing stick and is reputable for the treatment of various diseases such as fever, mouth infections, jaundice, guinea worm sores, dysentery, colic and joint rheumatism (Irvine 1961; Oliver-Bever 1986; Parveen et al. 2007). The antimicrobial activities of different parts of the plants have been reported (Oskoueian et al. 2011; Namuli et al. 2011; Garba and Okeniyi 2012).

Although, some literatures have reported the antibacterial activities of *J. curcas*, essential oils from *D. tripetala* and *S. aromaticum* but there is no much research studies on the formulation of toothpaste using ethanolic extract of these plants, either singly or in combined form as reported herein. In this regard, this research work aim to provide information on the chemical components in ethanolic extract of *D. tripetala* and *S. aromaticum* through the use of gas-chromatography–mass spectrophotometer and to assess the antimicrobial activity of the formulated toothpastes formulated from these extracts against oral pathogenic microorganisms.

## Materials and methods

The *J. curcas* latex was obtained into a sterile amber bottle as liquid exudate from the cut stalk of leaves and young stem of *J. curcas* plants grown in Ilara-mokin, Ondo State. The dried seeds of *D. tripetala* (pepper fruit) were obtained from Relief Market, Oyingbo, Lagos State, while *S. aromaticum* (clove) buds were bought from King’s Market, Akure, Ondo State. The samples were sorted and stored in air tight glass bottles to protect them from contaminants. All plant samples were authenticated at the Department of Crop, Soil and Pest Management, The Federal University of Technology Akure, Nigeria. The dried seeds were ground to powder. Powdered samples were sieved to a particle size of 212–249 µm and used for the analysis. The entire chemicals used in this research are analytical grade obtained from Sigma Aldrich.

### Source of commercial toothpaste

**Fig. 1:**
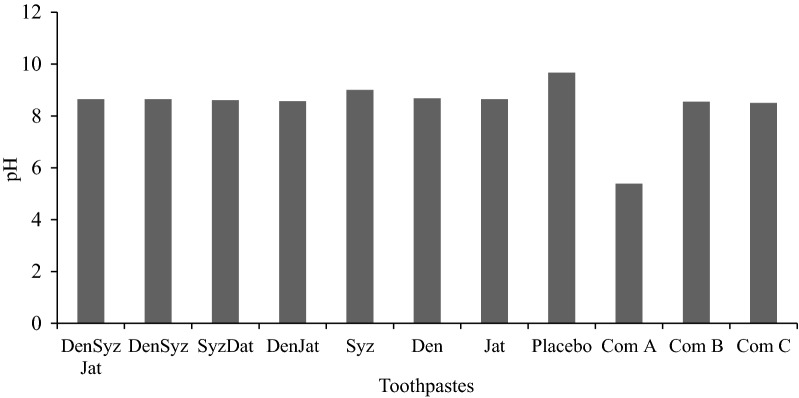
pH of the formulated and commercial toothpastes. DenSyzJatlax-(Triarotex)—*Dennettia tripetala, Syzygium aromaticum*, *Jatropha curcas* latex mixture. DenSyz-(Triaro)—*Dennettia tripetala, Syzygium aromaticum* mixture. SyzJatlax(Arotex)—*Syzygium aromaticum*, *Jatropha curcas* latex mixture. DenJatlax-(Tritex)—*Dennettia tripetala, Jatropha curcas* latex mixture. Syz-(Aro)—*Syzygium aromaticum* mixture. Den-(Tri)—*Dennettia tripetala*. Jatlax-(Tex)—*Jatropha curcas* latex. Placebo-without plant extracts material. Commercial fluoride toothpaste—Com A. Commercial herbal toothpaste—Com B. Commercial ordinary toothpaste—Com C

The branded toothpaste produced by companies were purchased and labelled as Com A, Com B, and Com C. The pH of commercially sold toothpastes was shown in Fig. 1. Com A contain sorbitol, aqua, hydrated silica, sodium lauryl sulphate, aroma, cellulose gum, trisodium phosphate, sodium phosphate, sodium fluoride, carbomer, polyethylene, limonene, Com B contain sorbitol, sodium hydroxide, glycerine, eugenol aqua, hydrated silica, sodium lauryl sulphate, cellulose gum, trisodium phosphate, sodium phosphate and Aloe vera plant, while Com C is made up of sodium fluoride, sorbitol, water, hydrated silica, sodium lauryl sulfate, flavor, cellulose gum, sodium saccharin without any herbal plant.

Oral B—Sorbitol, aqua, hydrated silica, sodium lauryl sulphate, aroma, cellulose gum, trisodium phosphate, sodium phosphate. Sodium saccharin, sodium chloride, carbomer, polyethylene, limonene, C177891, C142090.

Close Up—Sorbitol, aqua, hydrated silica, sodium lauryl sulphate, PEG-32, aroma, cellulose gum. Sodium saccharin, sodium chloride, zinc sulphate, mica, sodium hydroxide, glycerine, eugenol, C112490, C1I6035, C17700, C177491 C1 7789.

### Indicator microorganisms

Oral pathogenic microorganisms such as *Escherichia coli, Bacillus* sp., *Staphylococcus aureus, Staphylococcus epidermidis, Micrococcus luteus, Streptococcus mutans*, *Streptococcus pyogenes, Lactobacillus acidophilus, Candida albicans* were collected from Dental Department, Ondo State Specialist Hospital, Akure, while typed cultures viz; *S. aureus* (ATCC 29213), *Pseudomonas aeruginosa* (ATCC 27853), *E. coli* (ATCC 35218) and Methicillin-resistant *Staphylococcus aureus* (MRSA) were obtained from Medical Microbiology Department, University College Hospital, Ibadan.

### Preparation of ethanolic extract from plants

Exactly 100 g each of the powdered sample of *D. tripetala* seed and *S. aromaticum* clove were exhaustively extracted in 800 mL of 95% v/v ethanol using Soxhlet extractor. The extract from each sample was concentrated by exposing solvent contained extracted solution to air. The concentrated extracts were then kept separately in a clean and dried glass vial.

### Phytochemical screening of ethanolic extract from plants

Phytochemical screening for total phenols, saponins, flavonoids, and alkaloids were carried out using the standard procedure as follows:

### Test for Total Phenols

To 1 mL of the ethanolic extract of a plant sample, 3 mL of distilled water and few drops of neutral 5% FeCl_3_ solution were added. A dark green color confirmed the presence of phenolic compounds (Hayashi et al. 1998).

### Test for saponins

The equivalent of 2.0 g powdered sample was boiled in 20 mL of distilled water in a test tube in boiling water bath and filtered. About 10 mL of the filtrate was mixed with 5 mL of distilled water and shaken vigorously to form a stable persistent froth. The frothing was mixed with three drops of olive oil and shaken vigorously for the formation of emulsion characteristic of saponins (Obadoni and Ochuko 2001; Amos et al. 2015).

### Test for flavonoids

A 2.0 g portion of plant powder was heated with 10 mL of ethyl acetate in a test tube over a steam bath for 3 min. The mixture was filtered and 4 mL of the filtrate was shaken with 1 mL of dilute ammonia solution. Yellow coloration was observed that indicated the presence of flavonoids (Harborne 1973; Sofowora 1993).

### Test for alkaloids

A 0.5 g portion of the plant extract was stirred with 5 cm^3^ of 1% aqueous HCl on a steam bath. Few drops of picric acid solution were added to 2 cm^3^ of the extract. The formation of a reddish brown precipitate was taken as a preliminary evidence for the presence of alkaloids (Harborne 1973; Trease and Evans 1989).

### Gas chromatography–mass spectrometry analysis of plant extracts

The GC/MS analysis of the plant extracts was carried out at the Central Laboratory, the Federal University of Technology Akure, Ondo State in Nigeria. The isolated sample was analyzed on a wall coated gas chromatograph interfaced to a mass spectrometer (GC/MS, Agilent 7890A, US) instrument employing the following conditions: wall coated capillary column (BP-1; 50 m × 0.22 mm × 0.25 μm), which was programmed as follows: 60 °C to 70 °C at 2 k/min ending with a 20 min at 220 °C to 230 °C. The carrier gas was helium at a flow rate of 1 mL/min, split mode, with a ratio of 1:5 and injection volume of 1 μL in a CH_2_Cl_2_ and the ionization voltage, 70 eV.

### Toothpaste formulation

Toothpastes of various compositions were formulated using the ethanolic extracts of *D. tripetala* (pepper fruit) seeds, buds of *S. aromaticum* with *J. curcas* latex as the active ingredients and other none bioactive ingredients as given in Table 1. The pH of the formulated toothpastes from medicinal herbs was shown in Fig. 1.

**Table 1:** Composition of formulated toothpaste

Ingredient (g)	Composition							
	DenSyzJat	DenSyz	SyzJat	DenJat	Syz	Den	Jat	Negative control
*Syzygium aromaticum*	0.75	0.75	0.75	–	0.75	–	–	–
*Dennettia tripetala*	0.50	0.50	–	0.50	–	0.50		–
*Jatropha curcas* latex	5.00	–	5.00	5.00	–	–	5.00	–
10% CMC (w/w) in solution of 5% sorbitol	7.00	7.00	7.00	7.00	7.00	7.00	7.00	7.00
Calcium carbonate	2.00	2.00	2.00	2.00	2.00	2.00	2.00	2.00
Glycerine	2.00	2.00	2.00	2.00	2.00	2.00	2.00	2.00
Distilled water	5.00	5.00	5.00	5.00	5.00	5.00	5.00	5.00

The active ingredients contained in each of the paste are given as follows

DenSyzJat-(Triarotex)—*Dennettia tripetala, Syzygium aromaticum,* and *Jatropha curcas* latex

DenSyz-(Triaro)—*Dennettia tripetala* and *Syzygium aromaticum* as the active ingredients

SyzJat(Arotex)—*Syzygium aromaticum* and *Jatropha curcas* latex as active ingredients

DenJat-(Tritex)—*Dennettia tripetala* and *Jatropha curcas* latex

Syz-(Aro)—*Syzygium aromaticum* mixture

Den-(Tri)—*Dennettia tripetala*

Jat-(Tex)—*Jatropha curcas* latex

Negative control—without plant extracts material

### Determination of pH of the toothpaste

The net quantity of 5 g of sample was accurately weighed and placed in a 150 mL beaker. To this, 45 mL of freshly boiled and cooled water was added at 27 °C. It was stirred well to make a thorough suspension. The pH was determined within 5 min by using pH-016A benchtop pH meter (Dave et al. 2014).

### Antimicrobial activity of toothpaste against indicator microorganisms

Agar well diffusion method described by Cheesbrough (2000) and was adopted for assessing the inhibitory zone displayed by the formulated toothpastes. Briefly, the indicator microorganisms were sub cultured into nutrient broth and malt extract broth. The test tubes containing bacteria were incubated at 37 °C for 24 h, while tube with yeast was incubated at 28 °C for 48 h. Thereafter, inoculum size was adjusted to 0.5 McFarland turbidity standards. A sterile cotton swab was aseptically used to transfer organism on the dried surface of sterile Mueller Hinton Agar plate. Sterile cork borer was used to make well of 7 mm.

The formulated toothpaste was adjusted to 20 mg/mL and sterilized using Millipore filter (0.22 μm), which was introduced into the well in the Petri dishes that have already inoculated with indicator microorganisms. Commercial antibiotics such as flucloxacillin and ketoconazole were used as positive control for bacteria and yeast respectively, while sterile distilled water was used as negative control.

### Determination of minimum inhibitory concentration (MIC) of the toothpaste

The concentration of toothpaste was varied from 2.5 to 20 mg/mL and mixed with sterile nutrient broth and 0.1 mL of standardized inoculum (0.5 McFarland turbidity standards) in test tubes (Cheesbrough 2000). The tubes containing bacterial isolates were incubated aerobically at 37 °C for 24 h, while the yeast was incubated at 28 °C for 48 h. The Test tubes containing the growth medium and the inoculum of each microorganism were maintained as a control. The lowest concentration of the extract that produced no visible growth (no turbidity) when compared to the control tubes was regarded as MIC.

### Statistical analysis

The data obtained in triplicate were analyzed by Probit Analysis using Duncan’s Multiple Range Test (DMRT) and Analysis of Variance (ANOVA).

## Results

The qualitative phytochemical screening of the plant materials indicated the presence of some secondary metabolites namely; alkaloids, saponins, flavonoids and phenolics (Table 2). The percentage chemical composition of present compounds as detected by GC/MS analysis of the ethanolic extracts (Additional files 1 and 2) of the two plant materials is presented in Table 3. It was revealed that *S. aromaticum* contained some other bioactive chemical compounds in addition to all the four bioactive chemical components found in the seed of *D. tripetala*. The various chemical components found in *Dennettia tripetala* are the followings, 2-methylbenzyl alcohol, eugenol, nerolidol and 9-Octadecenoic acid (Oleic acid) while, 2-methylbenzyl alcohol, eugenol, caryophyllene, 1, 5-dimethyl-1-vinyl-4-hexenyl butyrate, 1-methyltricyclo [2.2.1.0 (1,6)] heptane, nerolidol, 9,12-octadecadienoic acid (Z, Z) (linoleic acid), 2-methyl-Z,Z-3, 13-octadecadienol, 9-octadecenoic acid, 9-octadecenoic acid (Z)- 2,3-dihydroxypropyl ester, 1,19-eicosadiene, 9-octadecenoic acid (Z)-, 2 hydroxyethyl ester, trichloroacetic acid, undec-2-enyl ester, 1,3,12-nonadecatriene, 9-octadecenal (Z) and Di (Z) hex-3-enyl phthalate are detected in the bud of *S. aromaticum*.

**Table 2:** Qualitative phytochemical composition of plant samples

Phytochemical	Plants	
	*Dennettia tripetala*	*Syzygium aromaticum*
Alkaloids	++	+++
Saponins	+++	++
Flavonoids	++	+++
Phenolics	+++	++

+ sign indicates the presence and the strength of the phytochemical constituent

+: traces, ++: moderate, +++: major

**Table 3:** The chemical composition of *Dennettia tripetala* and *Syzygium aromaticum*

Identified compound	*Dennettia tripetala*		*Syzygium aromaticum*	
	Retention time (min)	Relative composition (%)	Retention time (min)	Relative composition (%)
2-Methylbenzyl alcohol	20.050	85.34	16.118	57.57
Eugenol	24.770	3.62	20.386	2.90
Caryophyllene	Nil	Nil	24.907	1.12
1,5-Dimethyl-1-vinyl-4-hexenyl butyrate	Nil	Nil	26.721	0.52
1-Methyltricyclo [2.2.1.0 (1,6)] heptane	Nil	Nil	30.005	0.38
Nerolidol	37.673	4.72	32.792	3.36
9,12-Octadecadienoic acid (Z, Z)	Nil	Nil	61.287	11.80
2-Methyl-Z,Z-3,13-octadecadienol	Nil	Nil	62.071	3.30
9-Octadecenoic acid	80.479	6.32	62.220	2.20
9-Octadecenoic acid (Z)-, 2,3-dihydroxypropyl ester	Nil	Nil	63.512	4.39
1,19-Eicosadiene	Nil	Nil	67.976	0.80
9-Octadecenoic acid (Z)-, 2 hydroxyethyl ester	Nil	Nil	73.486	1.21
Trichloroacetic acid, undec-2-enyl ester	Nil	Nil	73.841	1.49
1,3,12-Nonadecatriene	Nil	Nil	75.066	1.18
9-Octadecenal (Z)	Nil	Nil	75.455	6.69
Di (Z) hex-3-enyl phthalate	Nil	Nil	78.007	1.10

The antimicrobial activities of the formulated pastes against test microorganisms are presented in Table 4. The formulated toothpastes labeled as DenSyzJat, DenSyz and SyzJat exhibited antimicrobial property against all the tested microorganisms with inhibition zones ranged from 10.7 to 18.3 mm, 7.3 to 14.6 mm and 8.33 to 14.4 mm respectively (Table 4). The following bacteria; *Bacillus* sp., *S*. *aureus*, *S*. *mutans* and *P*. *aeruginosa* were not susceptible to DenJat at 20 mg/mL. Formulated toothpaste with only *S. aromaticum* extract (Syz) seems to be much more active among the three bioactive materials used for the formulation, having recorded zone of inhibition of 14.0 mm against *S*. *aureus* when compared (P < 0.05) to Jat and Den paste with inhibition zones of 10.0 mm and 8.0 mm against *Lactobacillus acidophilus*. Com C, commercially produced toothpaste without any herb product has no inhibition against tested microorganisms.

**Table 4:** Zones of inhibition displayed by the formulated pastes against tested microorganisms at 20 mg/mL

Toothpaste	*E. coli*	*Bacillus* sp.	*S. aureus*	*S. epidermidis*	*M. luteus*	*S. mutans*	*C. albicans*	*S. pyogenes*	*L. acidophilus*	MRSA	*S. aureus* (ATCC 29213)	*P. aeruginosa* (ATCC 27853)	*E. coli* (ATCC 35218)
DenSyzJat	13.8^e^ ± 0.58	10.00^cd^ ± 0.00	18.00^d^ ± 0.0	14.00^e^ ± 0.00	17.7^g^ ± 0.6	17.67^d^ ± 0.58	16.30^e^± 0.0	10.70^c^ ± 0.01	18.30^f^ ± 0.02	13.90^d^ ± 0.58	15.00^e^ ± 0.0	11.30^cd^ ± 0.58	12.80^d^ ± 0.05
DenSyz	8.00^c^ ± 0.00	8.33^c^ ± 0.58	14.67^c^ ± 0.58	10.83^d^ ± 0.06	10.00^d^ ± 0.00	9.27^b^ ± 0.30	10.30^c^ ± 0.0	7.30^b^ ± 0.0	13.30^d^ ± 0.03	10.00^c^ ± 0.0	11.00^d^ ± 0.0	9.70^c^ ± 0.04	9.00^c^ ± 0.03
SyzJat	10.00^d^ ± 0.00	9.00^c^ ± 0.02	15.67^c^ ± 0.26	10.67^d^ ± 0.06	13.00^e^ ± 0.02	12.00^c^ ± 0.02	12.00^d^ ± 0.0	9.30^c^ ± 0.0	16.00^e^ ± 0.0	10.00^c^ ± 0.0	13.10^e^ ± 0.00	10.00^c^ ± 0.0	11.30^d^ ± 0.56
DenJat	5.00^b^ ± 0.01	0.00^a^	0.00^a^	8.33^c^ ± 0.58	15.00^f^ ± 0.00	0.00^a^	14.40^d^ ± 0.0	0.0^a^	12.70^d^ ± 0.01	9.80^c^ ± 0.0	9.30^c^ ± 0.00	0.0^a^	8.80^c^ ± 0.02
Syz	6.67^b,c^ ± 0.58	0.00^a^	14.00^c^ ± 0.00	10.30^d^ ± 0.58	0.00^a^	7.67^b^ ± 0.58	6.00^b^ ± 0.0	6.30^b^ ± 0.0	8.40^c^ ± 0.02	7.30^b^ ± 0.53	0.0^a^	7.00^b^ ± 0.0	8.60^c^ ± 0.54
Den	0.00^a^	0.00^a^	0.00^a^	0.00^a^	4.33^b^ ± 0.58	0.00^a^	6.00^b^ ± 0.0	0.0^a^	8.00^c^ ± 0.0	0.0^a^	6.70^b^ ± 0.00	0.0^a^	0.0a
Jat	4.00^b^ ± 0.0	4.67^b^ ± 0.58	7.33^b^ ± 0.58	0.00a	8.33^c^ ± 0.58	0.00^a^	8.70^c^ ± 0.2	6.60^b^ ± 0.0	10.00^d^ ± 0.0	8.00^b^ ± 0.0	0.0^a^	7.33^b^ ± 0.0	6.00^b^ ± 0.00
Com A	0.00^a^	0.00^a^	6.67^b^ ± 0.58	6.00^b^ ± 0.00	4.00^b^ ± 0.00	0.00^a^	5.00^b^ ± 0.0	0.0^a^	5.00^b^ ± 0.0	0.0^a^	5.00^b^ ± 0.02	0.0^a^	0.0^a^
Com B	0.00^a^	0.00^a^	6.67^b^ ± 0.19	6.00^b^ ± 0.19	4.00^b^ ± 0.19	0.00^a^	4.80^b^ ± 0.0	0.0^a^	5.00^b^ ± 0.0	0.0^a^	4.60^b^ ± 0.02	0.0^a^	5.30^b^ ± 0.02
Com C	0.00^a^	0.00^a^	0.00^a^	0.00^a^	0.00^a^	0.00^a^	0.0^a^	0.0^a^	0.0^a^	0.0^a^	0.0^a^	0.0^a^	0.0^a^
Flucloxacillin^a^	16.00^f^ ± 0.00	8.00^c^ ± 0.00	14.67^c^ ± 0.58	21.00^f^ ± 0.00	18.00^g^ ± 0.20	16.78^d^ ± 0.49	21.30^f^ ± 0.0	12.3 ± 0.02^d^	16.60^e^ ± 0.0	15.1 ± 0.0^d^	14.00^e^ ± 0.0	7.60^b^ ± 0.02	10.30^cd^ ± 0.24
Glycerin	0.00^a^	0.00^a^	0.00^a^	0.00^a^	0.00^a^	0.00^a^	0.0^a^	0.0^a^	0.0^a^	0.0^a^	0.0^a^	0.0^a^	0.0^a^
Distilled water	0.00^a^	0.00^a^	0.00^a^	0.00^a^	0.00^a^	0.00^a^	0.0^a^	0.0^a^	0.0^a^	0.0^a^	0.0^a^	0.0^a^	0.0^a^

Values are mean ± SD of replicates (n = 3), means with different letters within a column are significantly different by Duncan (P < 0.05)

DenSyzJat-(Triarotex)—*Dennettia tripetala, Syzygium aromaticum*, and *Jatropha curcas* latex

DenSyz-(Triaro)—*Dennettia tripetala and Syzygium aromaticum* as the active ingredients

SyzJat(Arotex)—*Syzygium aromaticum* and *Jatropha curcas* latex as active ingredients

DenJat-(Tritex)—*Dennettia tripetala and Jatropha curcas* latex

Syz-(Aro)—*Syzygium aromaticum* mixture

Den-(Tri)—*Dennettia tripetala*

Jat-(Tex)—*Jatropha curcas* latex

Placebo-without plant extracts material

Commercial fluoride toothpaste—Com A

Commercial herbal toothpaste—Com B

Commercial ordinary toothpaste—Com C

^a^Yeast was tested against ketoconazole

In Table 5, the minimum inhibitory concentration (MIC) of the formulated paste (DenSyzJat) containing raw materials from three plants ranged from 2.5 to 5.0 mg/mL, followed by paste with two plant materials; SyzJat (2.5–10 mg/mL) and DenSyz (5.0 to 10 mg/mL). The MIC value of DenJat is within 10 to 20 mg/mL, while the paste with single plant labelled as Szy, Den and Jat ranged from 5 to 20 mg/mL, 10 to 20 mg/mL and 5 to 20 mg/mL respectively.

**Table 5:** minimum inhibitory concentration (mg/mL) of the formulated paste against tested microorganisms

Toothpaste	*E. coli*	*Bacillus* sp.	*S. aureus*	*S. epidermis*	*M. luteus*	*S. mutans*	*C. albicans*	*S. pyogenes*	*L. acidophilus*	MRSA	*S. aureus* (ATCC 29213)	*P. aeruginosa* (ATCC 27853)	*E. coli* (ATCC 35218)
DenSyzJat	2.50	5.00	5.00	5.00	5.00	5.00	5.0	5.0	2.5	2.5	2.5	5.0	5.0
DenSyz	5.00	5.00	5.00	5.00	5.00	10.0	10.0	5.0	5.0	5.0	5.0	10.0	10.0
SyzJat	5.0	10.00	10.00	5.00	5.00	5.0	5.0	5.0	2.5	5.0	2.5	10.0	10.0
DenJat	10.00	0.0	0.0	10.00	10.00	0.0	10.0	0.0	10.0	10.0	10.0	20.0	10.0
Syz	10.00	0.0	10.0	10.0	0.0	20.0	20.0	10.0	10.0	5.0	0.0	0.0	20.0
Den	0.0	0.0	0.0	0.0	20.0	0.0	20.0	0.0	20.0	0.0	10.0	0.0	0.0
Jat	10.00	10.0	10.0	0.0	20.00	0.0	10.0	5.0	10.0	0.0	0.0	0.0	10.0
Com A	0.0	0.0	20.0	20.0	20.0	0.0	20.0	0.0	20.0	20	20.0	0.0	0.0
Com B	0.0	0.0	20.0	10.00	10.00	0.0	20.0	0.0	20.0	0.0	20.0	0.0	20.0
Com C	0.0	0.0	0.0	0.0	0.0	0.0	0.0	0.0	0.0	0.0	0.0	0.0	0.0
Flucloxacillin	15.0	5.0	10.0	10.0	10.0	5.0	10.0	2.5	2.5	5.0	2.5	10	10.0
Glycerin	0.0	0.0	0.0	0.0	0.0	0.0	0.0	0.0	0.0	0.0	0.0	0.0	0.0
Distilled water	0.0	0.0	0.0	0.0	0.0	0.0	0.0	0.0	0.0	0.0	0.0	0.0	0.0

Values are means of triplicates (n = 3)

DenSyzJat-(Triarotex)—*Dennettia tripetala, Syzygium aromaticum*, and *Jatropha curcas* latex

DenSyz-(Triaro)—*Dennettia tripetala and Syzygium aromaticum* as the active ingredients

SyzJat(Arotex)—*Syzygium aromaticum* and *Jatropha curcas* latex as active ingredients

DenJat-(Tritex)—*Dennettia tripetala and Jatropha curcas* latex

Syz-(Aro)—*Syzygium aromaticum*

Den-(Tri)—*Dennettia tripetala*

Jat-(Tex)—*Jatropha curcas* latex

Placebo-without plant extracts material

Commercial fluoride toothpaste—Com A

Commercial herbal toothpaste—Com B

Commercial ordinary toothpaste—Com C

## Discussion

The importance of bioactive compounds like alkaloids, phenolics, flavonoids and some other secondary metabolites have been reported by Kala et al. (2010). The presence of these active ingredients would be an added advantage to the formulated toothpastes. Ihemeje et al. (2013); and Elekwa et al. (2011) have reported the detection of observed phytochemical compounds in *D. tripetala* (pepper fruit) seed, which are the basis for its medicinal prowess. The presence of alkaloids, flavonoids, total phenols, saponins among other phytochemicals has been attributed to antifungal, antimicrobial, antiinflammatory and antidiabetes of its seed (Reihemann et al. 1999; Sparg et al. 2004). Phytochemical compounds such as phenolics, alkaloids, and flavonoids were detected in *S. aromaticum* (clove) buds. In this study, the identified bioactive ingredients are valuable drug components that could be used as antimicrobial and analgesic. Hence, clove is a good pharmaceutical ingredient (Amin et al. 2013).

The bioactive ingredient like; 9-octadecanoic acid (30.53%), trichloroacetic acid, undec-2-enyl ester (12.69%) and caryophyllene (0.83%) are present in the essential oil of *D. tripetala,* while, eugenol (49.71%) and caryophyllene (18.94%), were detected in the buds oil *S. aromaticum* (Elekwa et al. 2011; Bhuiyan et al. 2010) and caryophyllene, eugenol. 9,12-octadecadienoic acid (Z, Z)- and octadecanoic acid among other compounds after found in the ethanolic extract of *S. aromaticum* (Hema et al. 2012). The low quantity of essential oils in the two plant extracts could be due to the method of extraction but essential oils are mostly extracted when steam distillation method is adopted. Also, the process of concentrating the extracts through exposure to air could have reduced some of the extracted essential oils. All these factors could have lowered the number of essential oils detected by GC/MS analysis.

The pH values recorded for the formulated toothpaste indicated that all have different pH values. Most of the formulated toothpastes and two of the commercial toothpastes (Com B and C) are in alkaline state pH region but Com A is in weak acidic pH region. The presence of tannin, alkaloid, flavonoids, and phenols in those extracts as indicated by the qualitative analysis (Table 2), which could have influenced the pH of the herbal pastes. Most of these phytochemicals contain a hydroxyl group in their chemical structures thus, causing the paste to be alkalinity in nature. The pH result obtained in this research work was in agreement with those reported by Oyewale (2005). The researcher analyzed 20 different types of commercial toothpaste and revealed varying pH values, about 60% have neutral pH, 35% have acidic pH (5–5.8) and only one has alkaline pH of 8.26. The finding of Oyewale (2005) was not in agreement with Hilgenberg et al. (2011) who reported pH of some commercial toothpastes as 7.87 for G1. Sorriso Dentes Brancos (Conventional toothpaste), 9.12 for G2. Close-UP Whitening (Whitening toothpaste) and 10.09 for G3. Sensodyne Branqueador (Whitening toothpaste). Also, antibacterial herbal toothpaste prepared using *Eugenia caryophyllus, Acacia nilotica* and *Mimusops elengi* as an active ingredient were reported to have pH ranged from 8.50 to 8.70 (Dave et al. 2014).

Greater bioactivity was recorded for the mixture of three or two extracts, which is could be the results of good synergy between the entire extracts. The inhibitory activity of Jat paste could be associated with the presence of bioactive phytochemicals in its latex. The *J. curcas* latex has been used to treat bacterial and fungal mouth infection due to the presence of alkaloids, saponins, tannins, glycosides, phenols, and flavonoids (Suhaili et al. 2011; Sharma et al. 2016). Hence, *J. curcas* has been a traditional toothbrush in some places and used for various trado-medicines. Also, gas chromatography-mass spectroscopy analysis of *J. curcas* latex revealed the presence of dotriacontane, pentatriacontane, hexatriacontane, 1,2-benzene dicarboxylic acid and β-sitosterol (Mahmoodi et al. 2012). The secondary metabolites of *J. curcas* have been reported to hinder the growth of various bacteria such as *P. aeruginosa E. coli, S. aureus, Enterococcus faecalis and Shigella flexneri* (Igbinosa et al. 2009; Sharma et al. 2016). Though, it was noticed from the phytochemical screening of the two plant materials used in this study and literature search on *J. curcas* that secondary metabolites, such as alkaloids, saponins, tannins, saponins and flavonoids present in these plant. These phytochemicals have been reported to possess good antibacterial activity against several oral caries microbes (Hassan et al. 2004). Notwithstanding, the bioactive compounds detected by the GC/MS seems to have a synergetic role with those phytochemicals contributing to their antimicrobial prowess. Many of these compounds have been reported to be good antimicrobial agents (Holopainen 2004; Kubo et al. 1993).

The good performance of Syz paste above than that of Den and Jat toothpaste, is likely to be a function of the other bioactive components such as caryophyllene, 1, 5-dimethyl-1-vinyl-4-hexenyl butyrate, 1-methyltricyclo [2.2.1.0 (1,6)] heptane, 9,12-octadecadienoic acid (Z,Z), 2-methyl-Z,Z-3, 13-octadecadienol, 9-octadecenoic acid (Z)- 2, 3-dihydroxypropyl ester, 1,19-Eicosadiene, 9-octadecenoic acid (Z)-,2 hydroxyethyl ester, trichloroacetic acid, undec-2-enyl ester, 1,3,12-nonadecatriene, 9-octadecenal (Z) and Di (Z) hex-3-enyl phthalate found in it, which may not be present in the *Jatropha curcas* latex and *Dennettia tripetala* extract. Plant extracts containing 1,19-eicosadiene, 9-octadecenal (Z) and 1, 5-dimethyl-1-vinyl-4-hexenyl butyrate have been reported to have good antimicrobial, insecticides, anti-inflammatory and nematicides activities (Kahsay and Unnithan 2016; Borrelli and Izzo 2000; Mahmoodi et al. 2012).

Findings of Mostafa et al. (2018) revealed that ethanolic extract of *S. aromaticum* showed potential antimicrobial activity against the highly susceptible strains of food borne bacteria namely; *S. aureus* and *P. aeruginosa* with MIC ranged from 2.5 to 5.0 mg/mL. In the studies of Okoh et al. (2016), the essential oil of *Dennettia tripetala* displayed pronounced inhibitory activity against pathogenic microorganisms with lower MIC of 0.05–0.2 mg/mL. The variability in MIC may be as result of extraction method, type or size of indictor microorganisms and quantity of phytochemicals in the plant extracts.

The result of the antimicrobial activities of the formulated toothpastes, commercial toothpastes, and the controls revealed that all have different inhibitory actions against the tested oral pathogen. The difference observed could be an indication of the biological strength of the phytochemicals contained in each of the plant extract used for the toothpaste formulation. This is evident in the none-bioactivity character of the paste formulated without any active ingredient (negative control) towards any of the microbes. It could be inferred that the presence of alkaloids, flavonoids, saponins, and phenols apart from other bioactive components detected by GC/MS in those plant materials could enhance their bioactivities against the pathogens. Alkaloids as a secondary metabolite have been used to develop various human drugs and have potential toxicity against foreign organism cell (Kam and Liew 2002). These bioactive compounds; phenols, have several hydroxyl groups, flavonoids whose ring structure consists of different phenolic hydroxyl groups and saponin with the characteristic foaming/bitter taste properties and consists of polycyclic aglycones attached to one or more sugar side chains, have been proved to possess various biological activities such as antimicrobial, anti-inflammatory, antiangiogenic, analgesic, anti-allergic, cytostatic and antioxidant (Hodek et al. 2002; Majinda 2012).

The presences of alkyl aryl alcohols (AAAs) fragrance such as benzyl alcohol, a-methylbenzyl alcohol, and phenethyl alcohol etc., have been reported to occur in plants (Belsito et al. 2012). Also, according to Pelczar et al. (1988), phyto-compounds with phenolic groups such as carvacrol, eugenol, and thymol possess antimicrobial activity against microbes due to the presence of alkyl substitution on the phenolic nucleus. It has been reported that these group of compounds could act as bactericidal or bacteriostatic agent. Thus the presence of 2-methylbenzyl alcohol and eugenol in the extracts of *D. tripetala* and *S. aromaticum* would have impacted antimicrobial activity to the pastes formulated from it. The bioactive activities of *D. tripetala* and *S. aromaticum* extract could also be linked to nerolidol, an aliphatic sesquiterpene alcohol. This has been reported to have good antibacterial and antifungal activities with skin penetration effect, which could make it dissolve the cell wall of the pathogenic microorganisms (Byron and Johnson 2003; Cornwell and Barry 1994; Srinivasan et al. 2016).

Although, *D. tripetala* has higher percentage of chemical components than *S. aromaticum*, but, there is presence of other chemical components such as 9,12-octadecadienoic acid (z, z) (linoleic acid), β-caryophyllene, 9-octadecenoic acid (Z)- 2, 3-dihydroxypropyl ester, nerolidol, (linoleic acid), 9-octadecenoic acid (oleic acid, 9-octadecenal, trichloroacetic acid undec-2-enyl ester and 5-dimethyl-1-vinyl-4-hexenyl butyrate in *Syzygium aromaticum*, all which have been documented to have good antimicrobial and medicinal properties (Holopainen 2004; Kubo et al. 1993; Amban et al. 2010; Sharma and Vijayvergia 2015; Kahsay and Unnithan 2016), hence aided their better antimicrobial activity. The antibacterial activity of caryophyllene has made it an ingredient for antiseptic products (Jadhav et al. 2004).

The synergistic activity of the essential oils, fatty acids and other secondary metabolites in the extracts used for the formulation of the toothpastes could also be an influential factor in their bioactivities. It is known that bacterial cell walls are made up of lipid fractions in plasma membranes (Trombetta et al. 2005).

Essential oils, which are known to be mixture of volatile lipophilic (fat loving, i.e., soluble in fat) and fatty acid present in those extracts, might pass through the cell wall and cytoplasmic membrane, disrupting the structural arrangement of different polysaccharides, fatty acids and phospholipids layers and this may affect others cellular structure leading to the death of the bacterial (Carson et al. 2002; Burt 2004; Longbottom et al. 2004). The destruction of the cell wall membrane of bacterial by the essential oil and the fatty acid could make the organism vulnerable to other phytochemicals such as flavonoids, saponins, tannins and alkaloid which been reported as antibacterial agents. According to Aliyu et al. (2012), saponin mode of antibacterial involves membranolytic properties rather than simply altering the surface tension of the extracellular medium. Thus, opening up of the membrane could activate the saponins antibacterial activity.

From this study, *S. aromaticum* has more essential oils, fatty acid and other chemical components like trichloroacetic acid undec-2-enyl ester, 1,3, 12-nonadecatriene, Di (Z) hex-3-enyl phthalate2-methyl-Z,Z-3, 13-octadecadienol, 1-methyltricyclo [2.2.1.0 (1,6)] heptane, 1, 5-dimethyl-1-vinyl-4-hexenyl butyrate and 9-octadecenoic acid (Z)- 2,3-dihydroxypropyl ester not present in Jatropha curcas latex and *D. tripetala*, this could have been reason for its better antimicrobial activity. The study of Hossain et al. (2014) reported no bioactive activity of essential oil from *S. aromaticum* clove against *P. aeruginosa*, although, strong activity was observed against *Staphylococcus* spp. and *E. coli.*

The antimicrobial study showed that the toothpaste formulated using plant extracts had better performance than all the commercial toothpastes. The Com C had no bioactivity against all the tested microbes, Com A and Com B had bioactivities against *S. aureus*, *S. epidermis,* and *Micrococcus luteus,* with lower zone of inhibition when compared to the plant extract pastes. DenSyzJat paste compared favorably well with the positive control and recorded better performance against some microbes, while other plant extracts paste compared moderately well with the positive control.

Arekemase et al. (2011) reported phytochemical analysis and antimicrobial activity of *Jatropha curcas* plant against some selected microorganisms, the extracts, and latex of *Jatropha curcas* displayed potent antimicrobial activity against *Staphylococcus aureus, Neisseria gonorrhea, Pseudomonas aeruginosa, Escherichia coli, Candida albicans,* and *Aspergillus flavus,* giving minimum inhibitory concentration as low as 0.5 mL. The results confirmed the potency of this plant in treating human infections including sexually transmitted diseases. Therefore, it is worthwhile to note that the plant extracts and exudates in the formulated toothpastes showed pronounced inhibitory activities against cariogenic organisms than the commonly used fluoridated tooth pastes. This implies that bioactive constituents of the plant extracts and latex could be useful raw material in producing toothpaste that may, which will reduce the occurrence of microbial pathogens associated with dental diseases.

In conclusion, the use of medicinal and edible plants for the formulation of toothpaste should be of interest in oral care products due to the presence of bioactive compounds that are capable of inhibiting the growth of microorganisms causing oral infection. The adoption of herbal toothpaste by consumers and dentists will safeguard the side effect of oral care products containing synthetic compounds and reduce the cost of treatment.

## Additional files

**Additional file 1.** The chromatogram of *Dennettia tripetala* extract. The GCMS revealed the library ID of bioactive compounds in the extract with their peaks, area, retention time, molecular formulae and respective weight.

**Additional file 2.** The chromatogram of *Syzygium aromaticum* extract. GCMS revealed the library ID of bioactive compounds in the extract with their peaks, area, retention time, molecular formulae and respective weight.
