# Supplementary material for: Antimicrobial potential of toothpaste formulated from extracts of Syzygium aromaticum, Dennettia tripetala and Jatropha curcas latex against some oral pathogenic microorganisms
Source: AMB Express. 2019 Feb 4;9:20. doi: 10.1186/s13568-019-0744-2 (PMC6362185; doi:10.1186/s13568-019-0744-2)
Supplement: Supplementary file 1 — Additional file 1. The chromatogram of Dennettia tripetala extract. The GCMS revealed the library ID of bioactive compounds in the extract with their peaks, area, retention time, molecular formulae and respective weight. [file 13568_2019_744_MOESM1_ESM.pdf]

Additional file 1

AMB Express

Antimicrobial Potential of Toothpaste formulated from Extracts of *Syzygium aromaticum*, *Dennettia tripetala* and *Jatropha curcas* latex against some Oral Pathogenic Microorganisms

Olugbenga Oludayo Oluwasina (oooluwasina@futa.edu.ng)[1*], Ifunanya Vivian Ezenwosu (oluwasinagbenga@yahoo.com)[1], Clement Olusola Ogidi (clementogidi@yahoo.com)[2,3] and Victor Olusegun Oyetayo (ovonew67@gmail.com)[2]

[1]Department of Chemistry, The Federal University of Technology, PMB 704, Akure, Nigeria

[2]Department of Microbiology, The Federal University of Technology, PMB 704, Akure, Nigeria

[3] Biotechnology Unit, Department of Biological Sciences, Kings University, PMB 555, Odeomu, Nigeria

***Corresponding author: oooluwasina@futa.edu.ng, +2348107246660

Additional file 1: the chromatogram of *Dennettia tripetala* extract. GCMS revealed the library ID of bioactive compounds in the extract with their peaks, area, retention time, molecular formulae and respective weight.

Scanned by CamScanner

mple Name: GBENGA 3
sc Info :
al Number: 1

Dennett

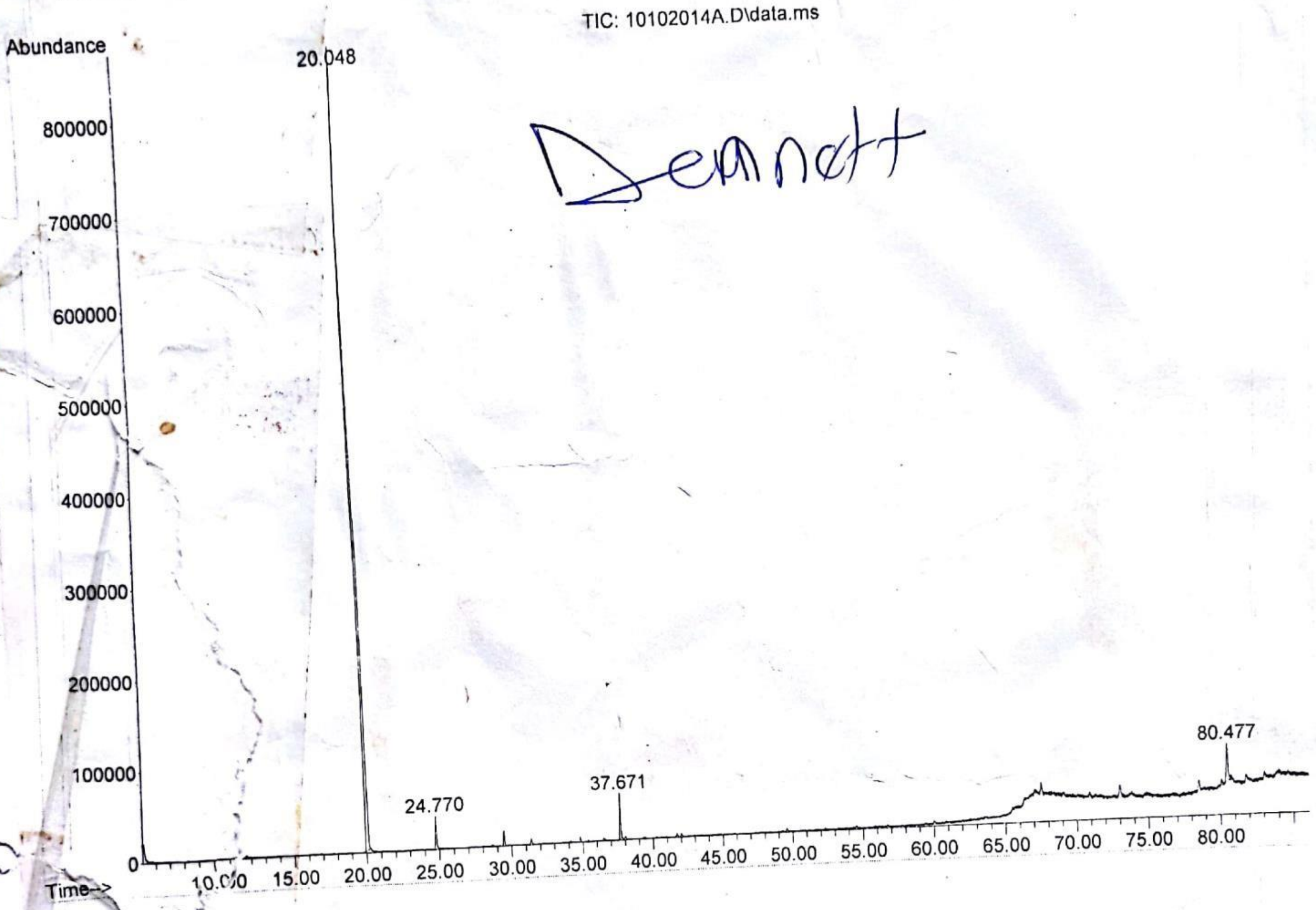

Linoleic acid

ample Name: GBENGA 3
Misc Info :
ial Number: 1

Dennen

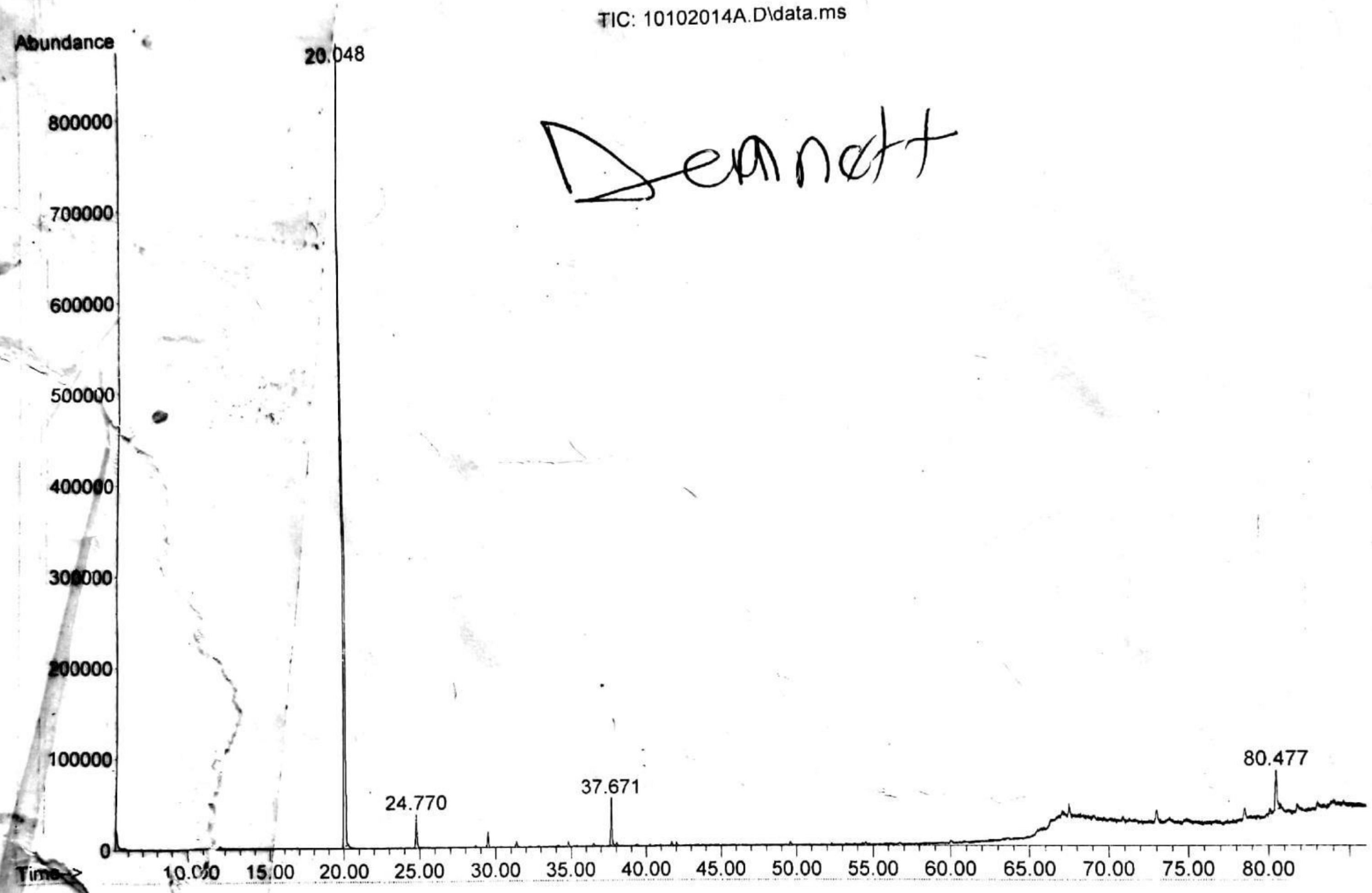

Dennott

Linolere acid

Scanned by CamScanner

Sample : GBENGA 3
Misc :
ALS Vial : 1 Sample Multiplier: 1

Integration Parameters: events.e
Integrator: ChemStation

Method : C:\MSDCHEM\1\METHODS\IBITOYE METHOD\IBITOYE METHOD.M
Title :

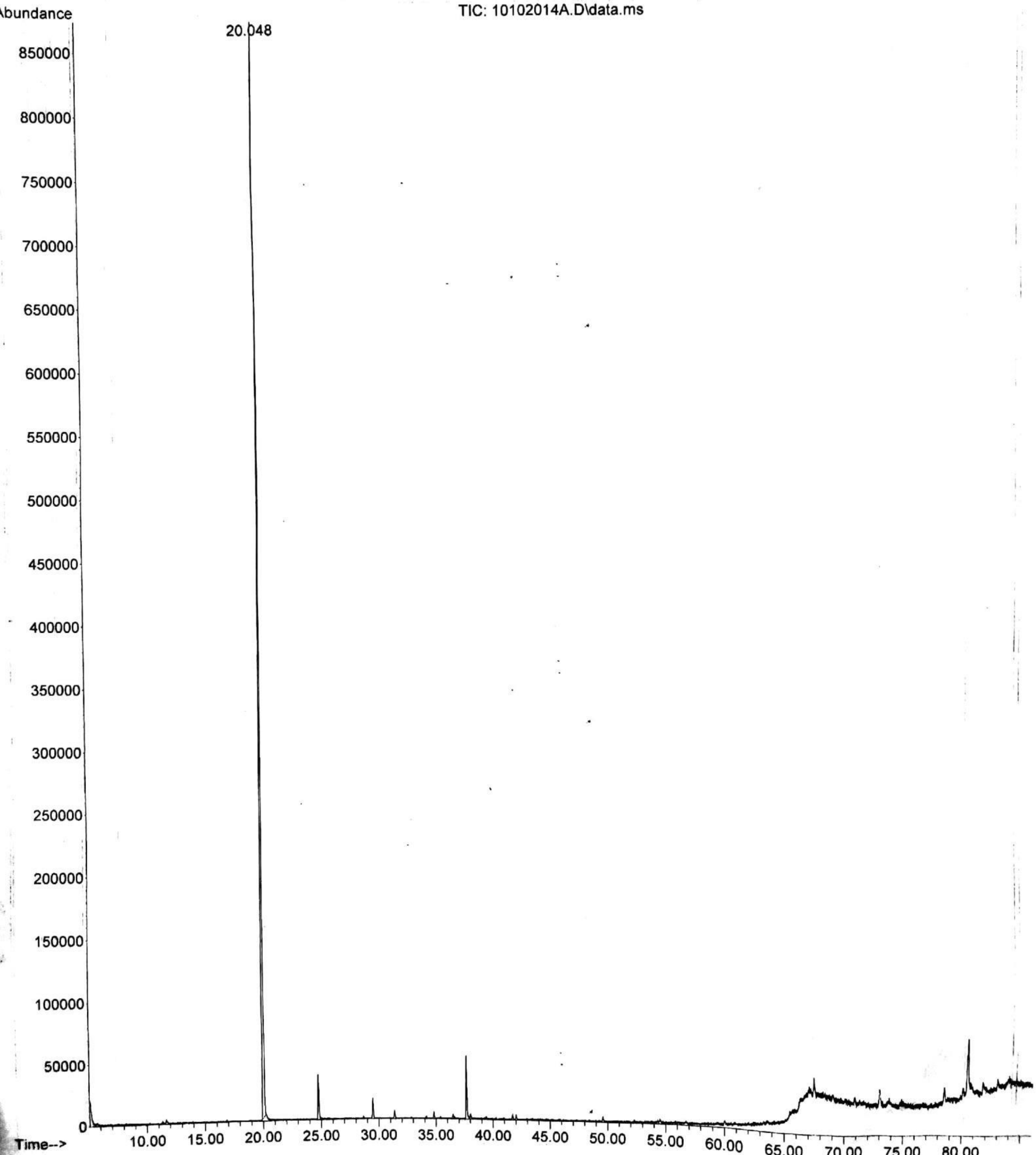

Scanned by CamScanner

Operator :
Sample : GBENGA 3
Misc :
ALS Vial : 1 Sample Multiplier: 1

Integration Parameters: events.e
Integrator: ChemStation

Method : C:\MSDCHEM\1\METHODS\IBITOYE METHOD\IBITOYE METHOD.M
Title :

Signal : TIC: 10102014A.D\data.ms

peak #	R.T. min	first scan	max scan	last scan	PK TY	peak height	corr. area	corr. % max.	% of total
1	20.048	2588	2615	2648	BB 3	870988	48784686	100.00%	100.000%

Sum of corrected areas: 48784686

Scanned by CamScanner

[illegible] : [illegible]
Sample : GBENGA 3
Misc :
ALS Vial : 1 Sample Multiplier: 1

Search Libraries: C:\Database\NIST11.L Minimum Quality: 0

Unknown Spectrum: Apex
Integration Events: ChemStation Integrator - autoint1.e

Pk#	RT	Area%	Library/ID	Ref#	CAS#	Qual
1	20.050	85.34	C:\Database\NIST11.L			
			Oxalic acid, 2-phenylethyl propyl ester	90433	1000309-65-5	64
			Formic acid, (2-methylphenyl)methy l ester	24532	1000368-93-3	64
			Benzenemethanol, 2-methyl-, acetat	33429	017373-93-2	59
2	24.770	3.62	C:\Database\NIST11.L			
			Eugenol	33238	000097-53-0	98
			Eugenol	33242	000097-53-0	98
			Phenol, 2-methoxy-4-(1-propenyl)-, (Z)-	33448	005912-86-7	96
3	37.673	4.72	C:\Database\NIST11.L			
			Nerolidol 2	79382	1000285-43-6	78
			3,7,11-Trimethyl-3-hydroxy-6,10-do decadien-1-yl acetate	129254	1000144-12-7	72
			1,6,10-Dodecatrien-3-ol, 3,7,11-tr imethyl-, [S-(Z)]-	79446	000142-50-7	72
4	80.479	6.32	C:\Database\NIST11.L			
			9-Octadecenoic acid, (E)-	129352	000112-79-8	86
			Octadec-9-enoic acid	129341	1000190-13-7	64
			Oleyl alcohol, trifluoroacetate	193100	1000352-68-4	55

Scanned by CamScanner

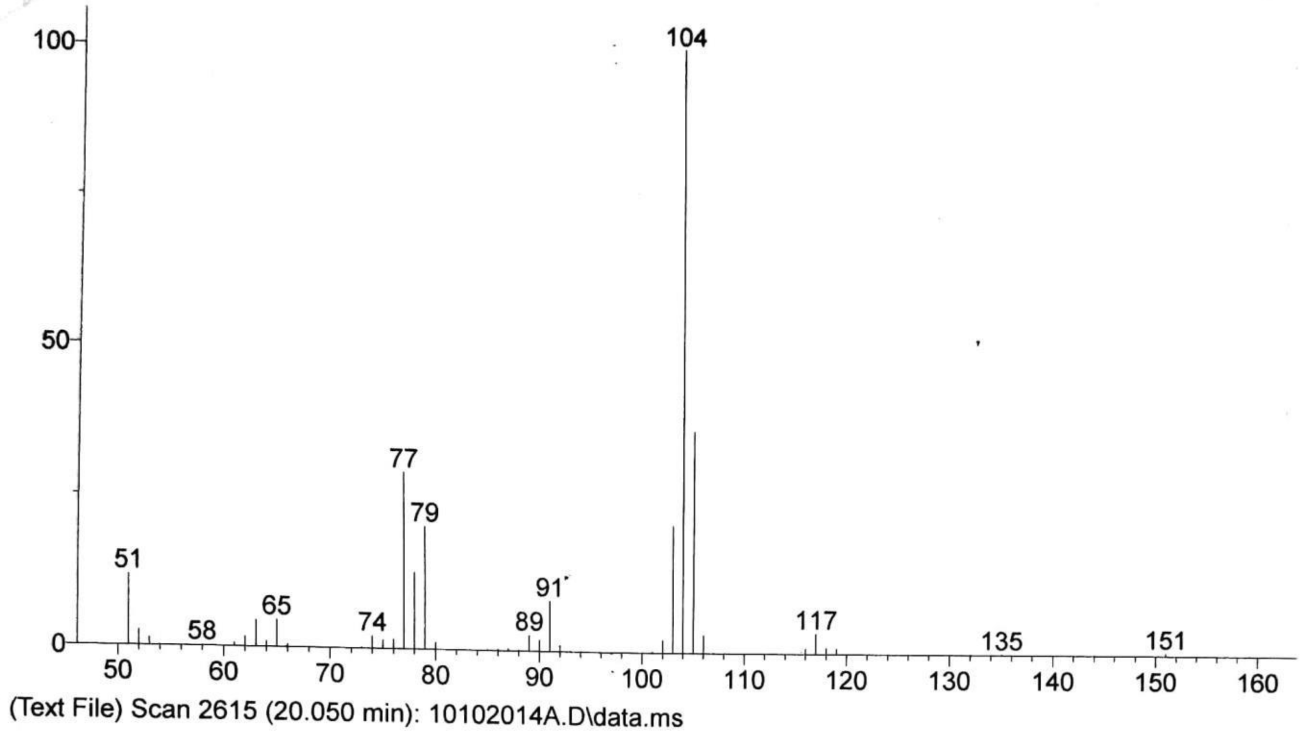

(Text File) Scan 2615 (20.050 min): 10102014A.D\data.ms

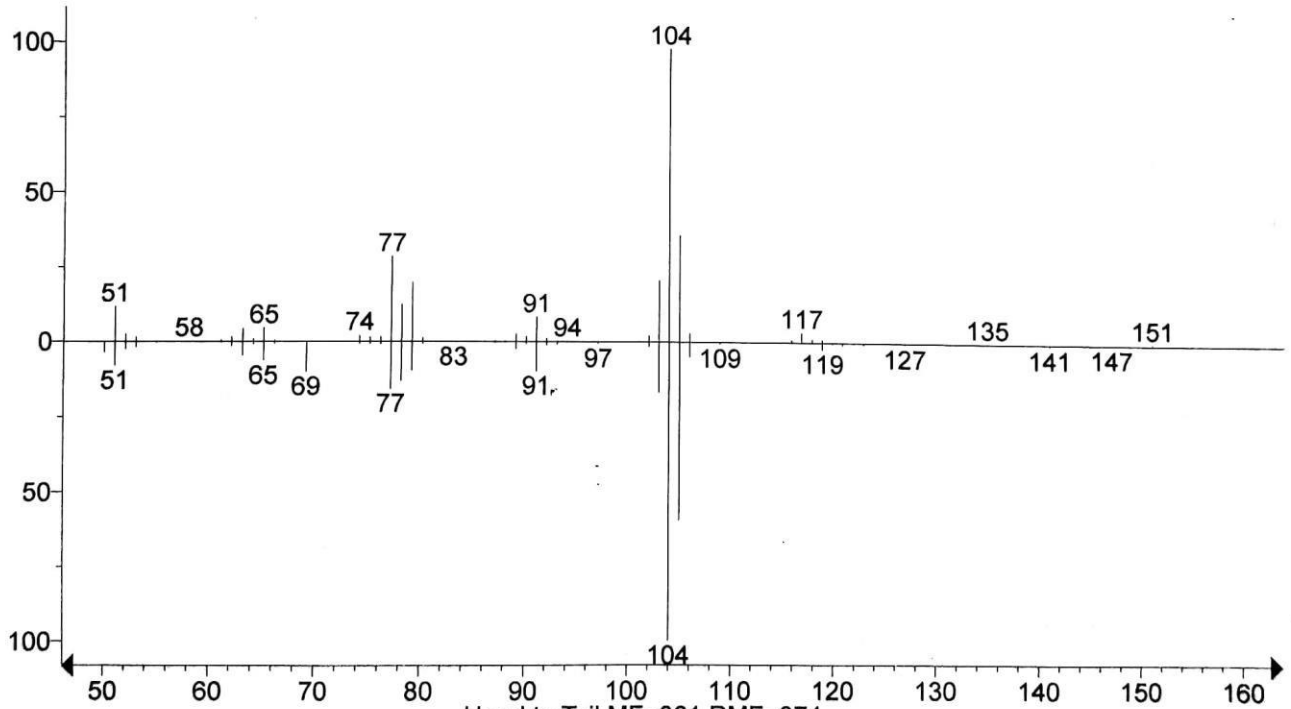

Head to Tail MF=861 RMF=874

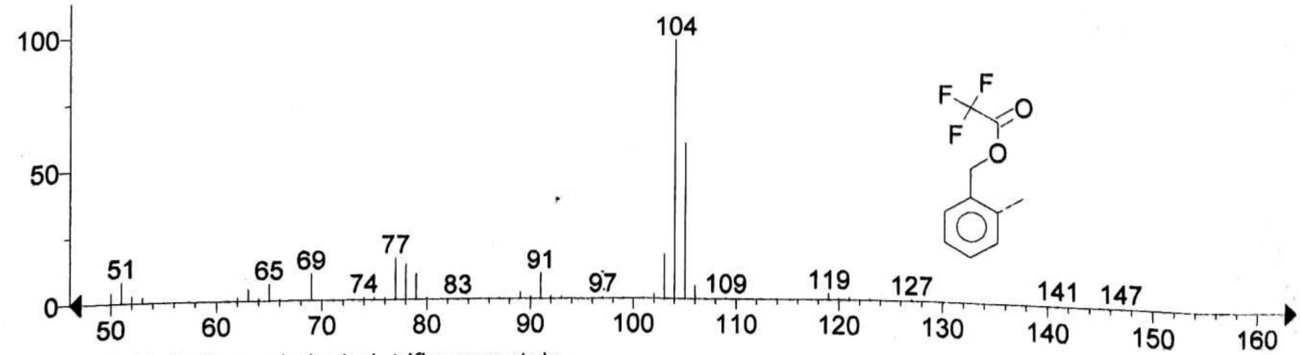

(mainlib) 2-Methylbenzyl alcohol, trifluoroacetate

Scanned by CamScanner

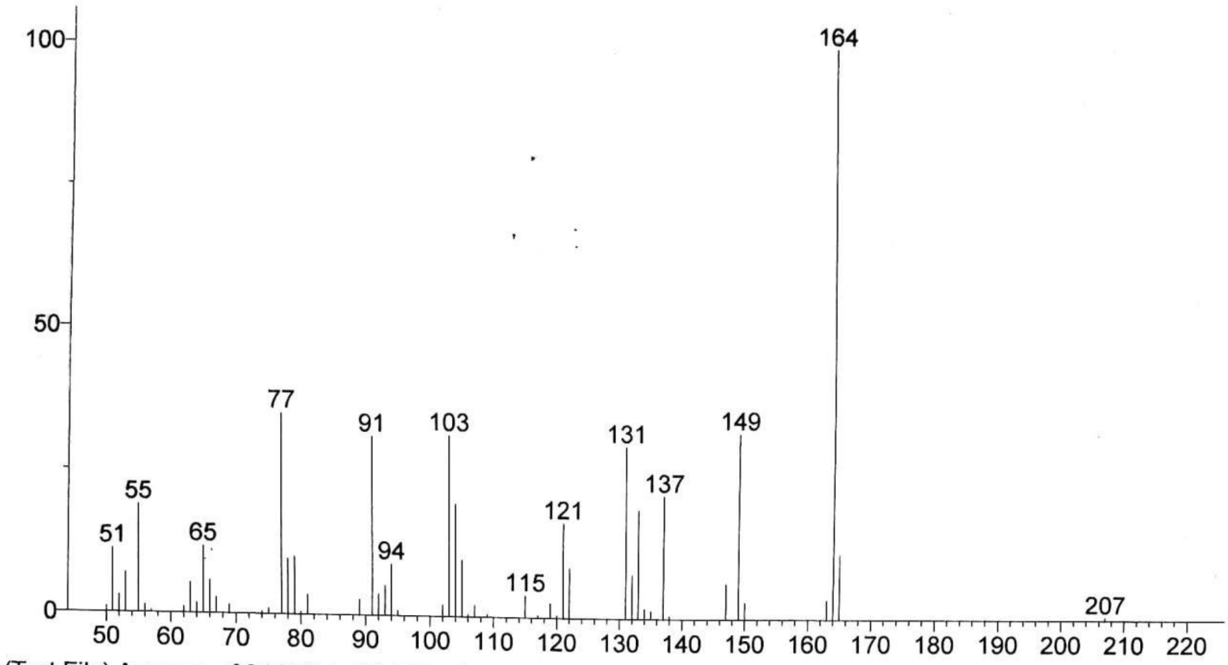

(Text File) Average of 24.770 to 24.799 min.: 10102014A.D\data.ms

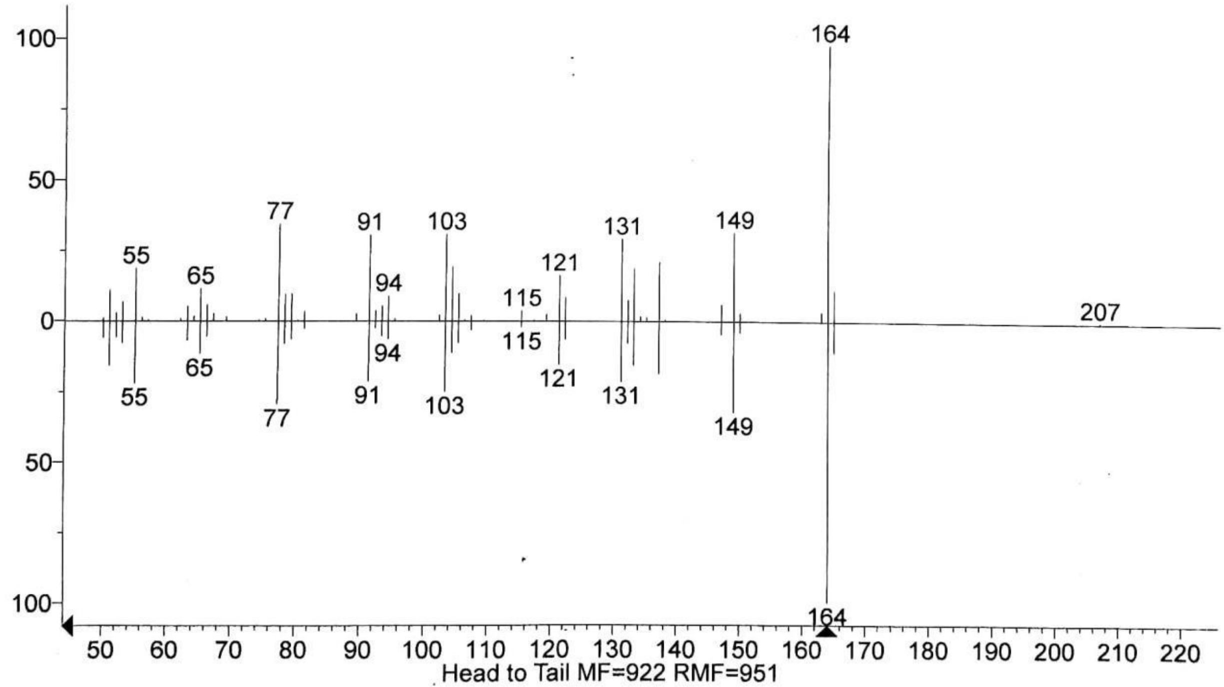

Head to Tail MF=922 RMF=951

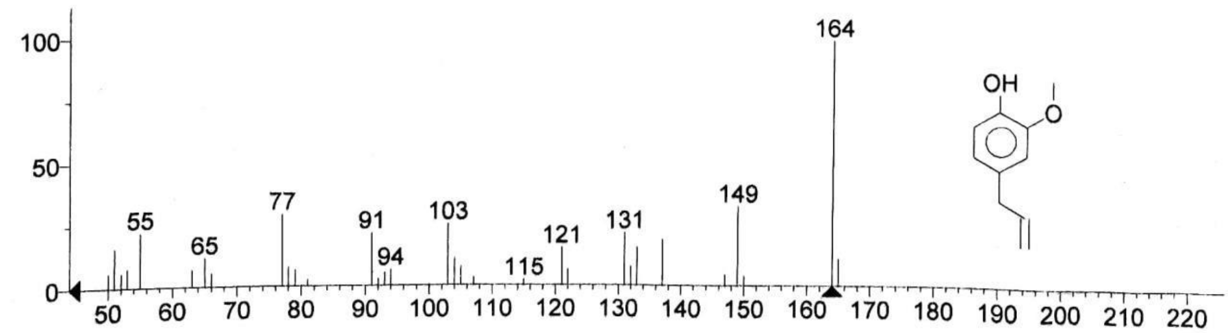

(replib) Eugenol

Scanned by CamScanner

Name: Eugenol
Formula: $C_{10}H_{12}O_2$
MW: 164 Exact Mass: 164.08373 CAS#: 97-53-0 NIST#: 21878 ID#: 23451 DB: replib
Other DBs: Fine, TSCA, RTECS, EPA, USP, HODOC, NIH, EINECS, IRDB
Contributor: K. VLADIMIR INSTITUTE OF CHEMISTRY BRATISLAVA CZECHOSLOVAKIA
10 largest peaks:

164 999 |	149 324 |	77 294 |	103 252 |	55 220 |
131 216 |	91 214 |	137 188 |	39 172 |	51 158 |

Synonyms:
1.Phenol, 2-methoxy-4-(2-propenyl)-
2.Phenol, 4-allyl-2-methoxy-
3.p-Allylguaiacol
4.p-Eugenol
5.Caryophyllic acid
6.Engenol
7.Eugenic acid
8.2-Methoxy-1-hydroxy-4-allylbenzene
9.2-Methoxy-4-allylphenol
10.4-Allyl-2-methoxyphenol
11.4-Allylguaiacol
12.4-Hydroxy-3-methoxyallylbenzene
13.NCI-C50453
14.1-Hydroxy-2-methoxy-4-allylbenzene
15.1-Hydroxy-2-methoxy-4-prop-2-enylbenzene
16.2-Methoxy-4-(2-propenyl)phenol
17.2-Methoxy-4-prop-2-enylphenol
18.4-Allyl-1-hydroxy-2-methoxybenzene
19.4-Allylcatechol-2-methyl ether
20.1,3,4-Eugenol
21.FA 100
22.FEMA No. 2467
23.2-Metoksy-4-allilofenol
24.2-Hydroxy-5-allylanisole
25.Allylguaiacol
26.4-(2-Propenyl)-2-methoxyphenol
27.NSC 209525
28.Phenol, 2-methoxy-4-(2-propen-1-yl)-

Estimated non-polar retention index (n-alkane scale):
Value: 1392 iu
Confidence interval (Diverse functional groups): 89(50%) 382(95%) iu

Retention index.
1. Value: 1337 iu
Column Type: Capillary
Column Class: Standard non-polar
Active Phase: BP-1
Column
Length: 50 m
Carrier Gas: He
Column Diameter: 0.22 mm
Phase Thickness: 0.25 um
Data Type: Linear
RI
Program Type: Ramp
Start T: 60 C
End T: 220 C
Heat Rate: 2 K/min

Scanned by CamScanner

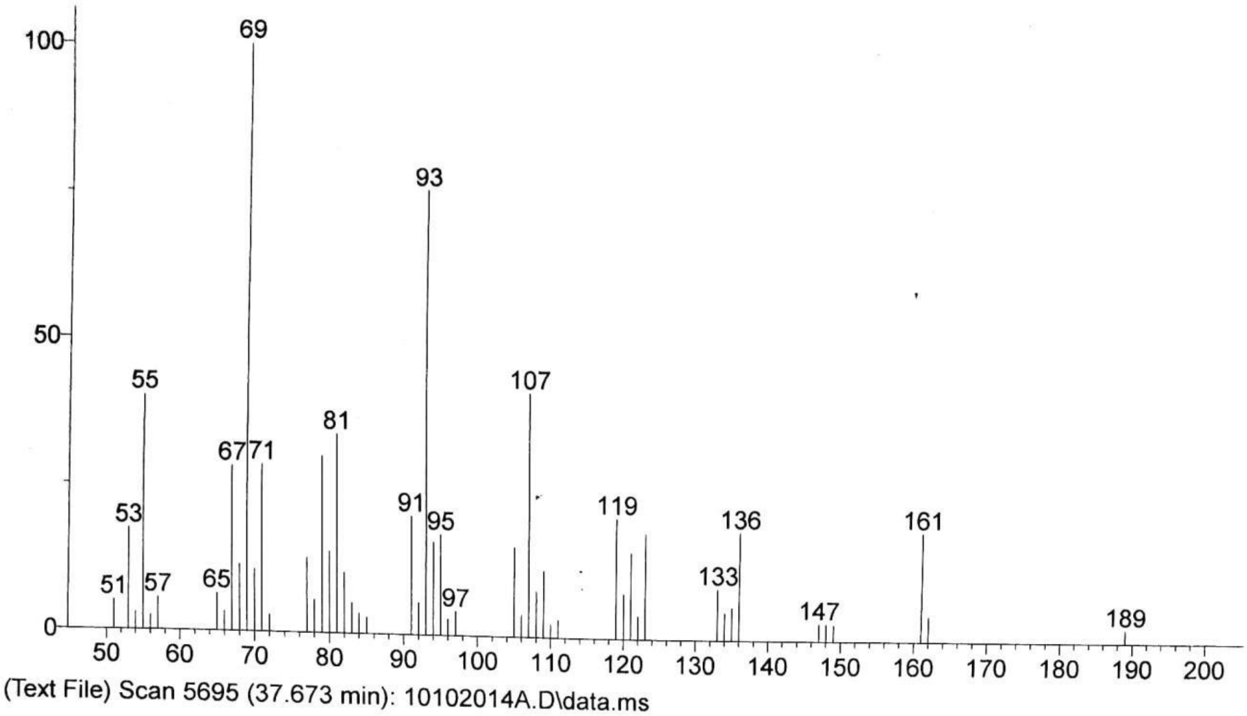

(Text File) Scan 5695 (37.673 min): 10102014A.D\data.ms

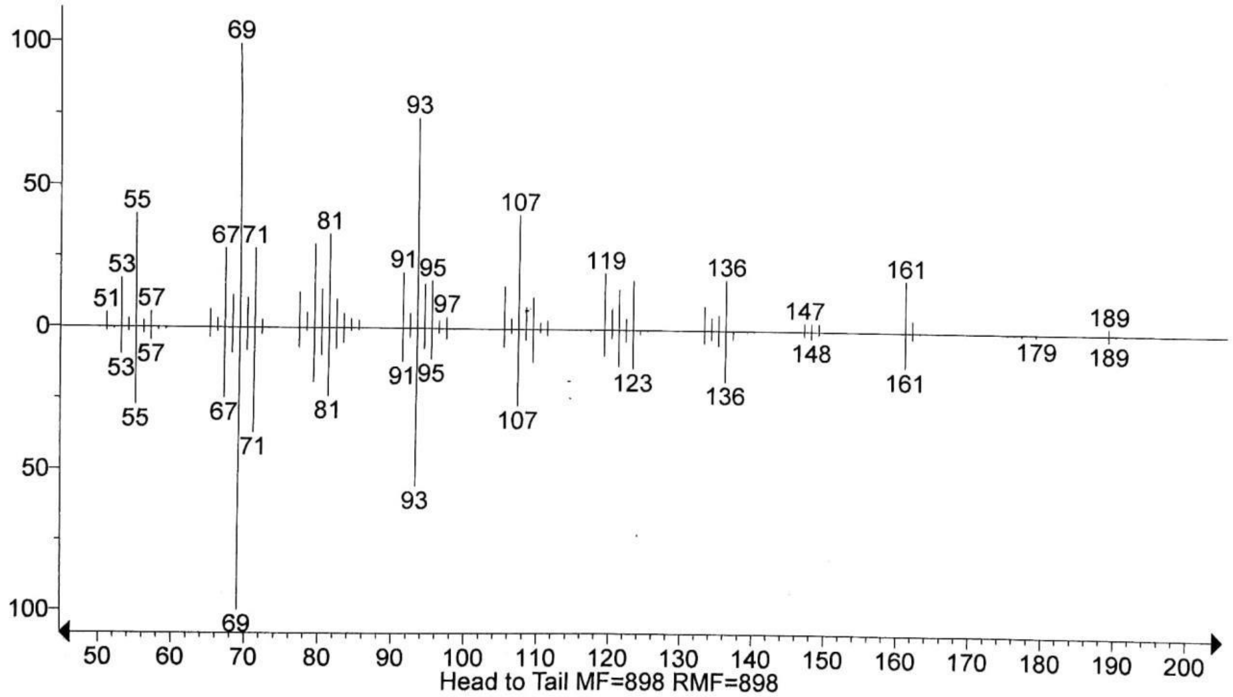

Head to Tail MF=898 RMF=898

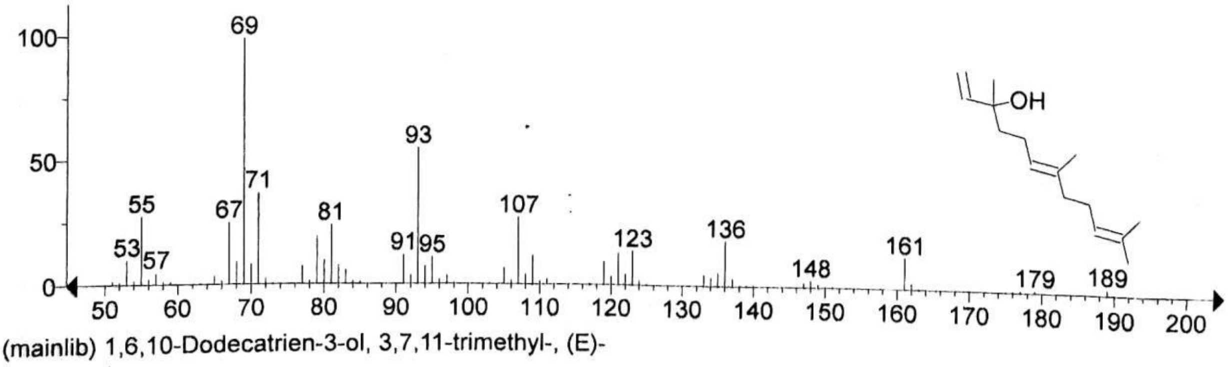

(mainlib) 1,6,10-Dodecatrien-3-ol, 3,7,11-trimethyl-, (E)-

Scanned by CamScanner

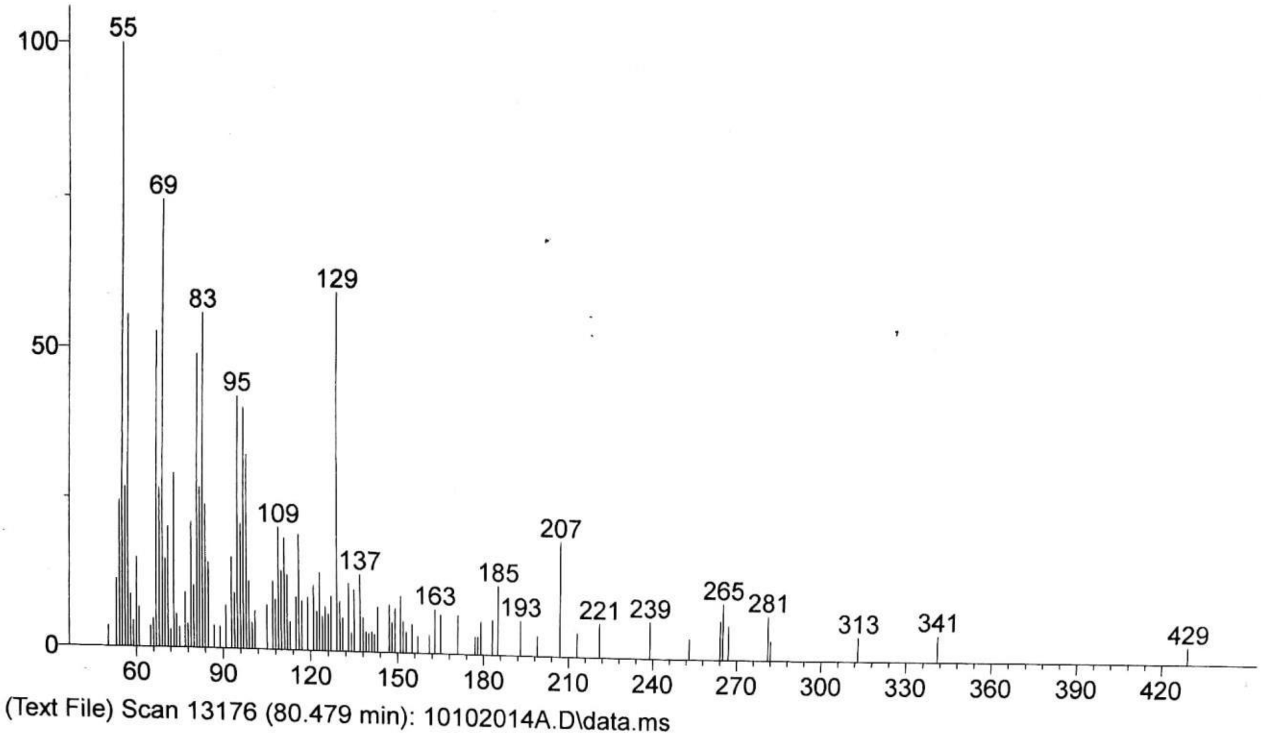

(Text File) Scan 13176 (80.479 min): 10102014A.D\data.ms

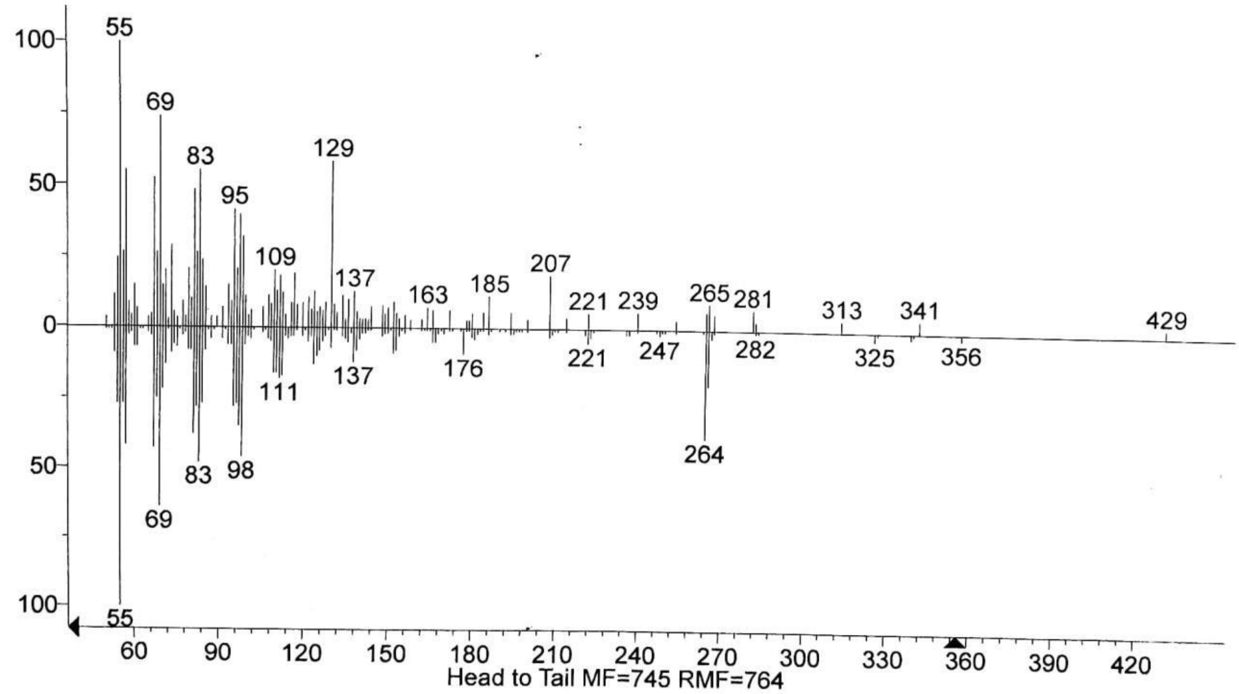

Head to Tail MF=745 RMF=764

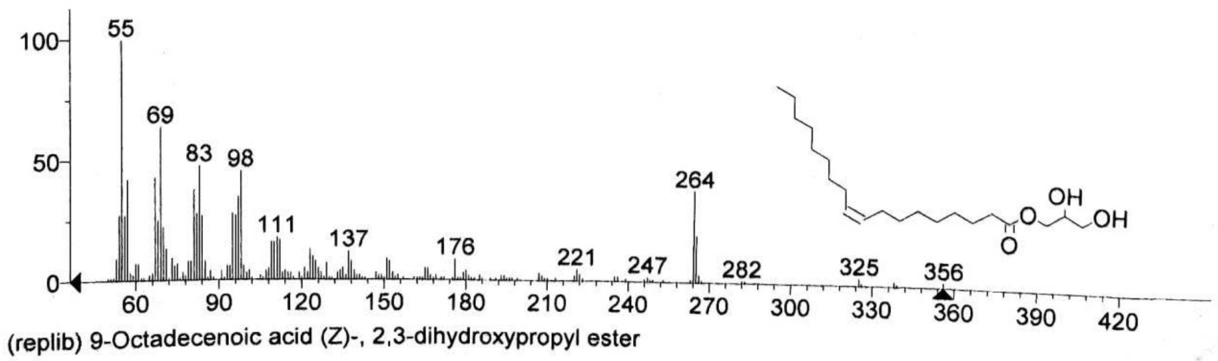

(replib) 9-Octadecenoic acid (Z)-, 2,3-dihydroxypropyl ester

Scanned by CamScanner

Instrument : GCMS [illegible] METHOD.M
Sample Name: GBENGA PEP CHEM DEPT.
Misc Info :
Vial Number: 1

Dennet

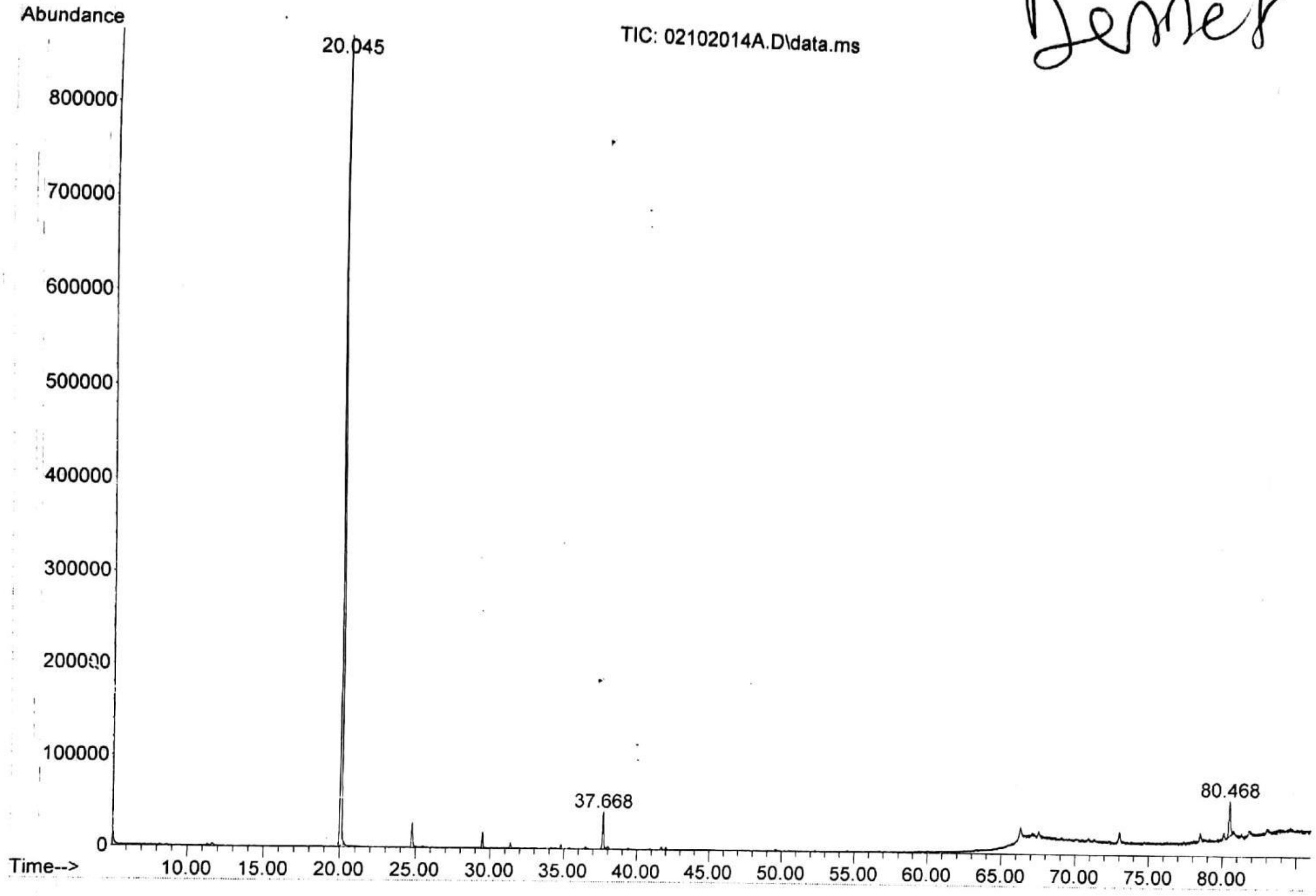

Scanned by CamScanner

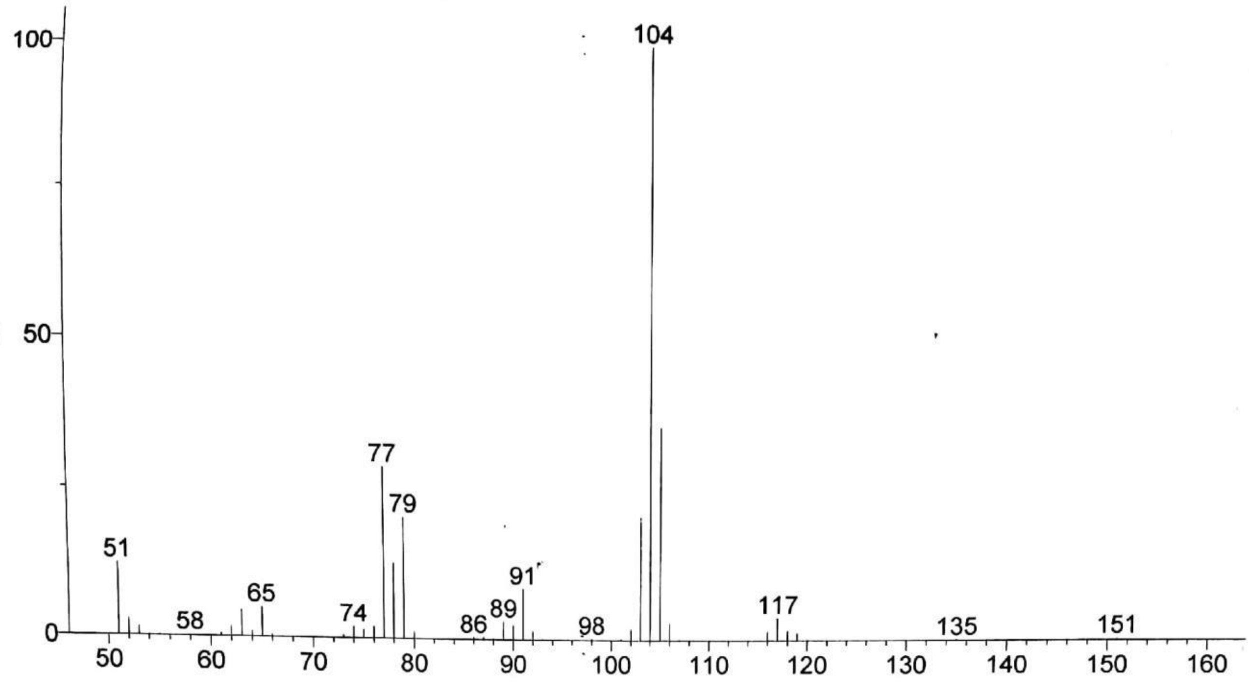

(Text File) Scan 2614 (20.044 min): 02102014A.D\data.ms

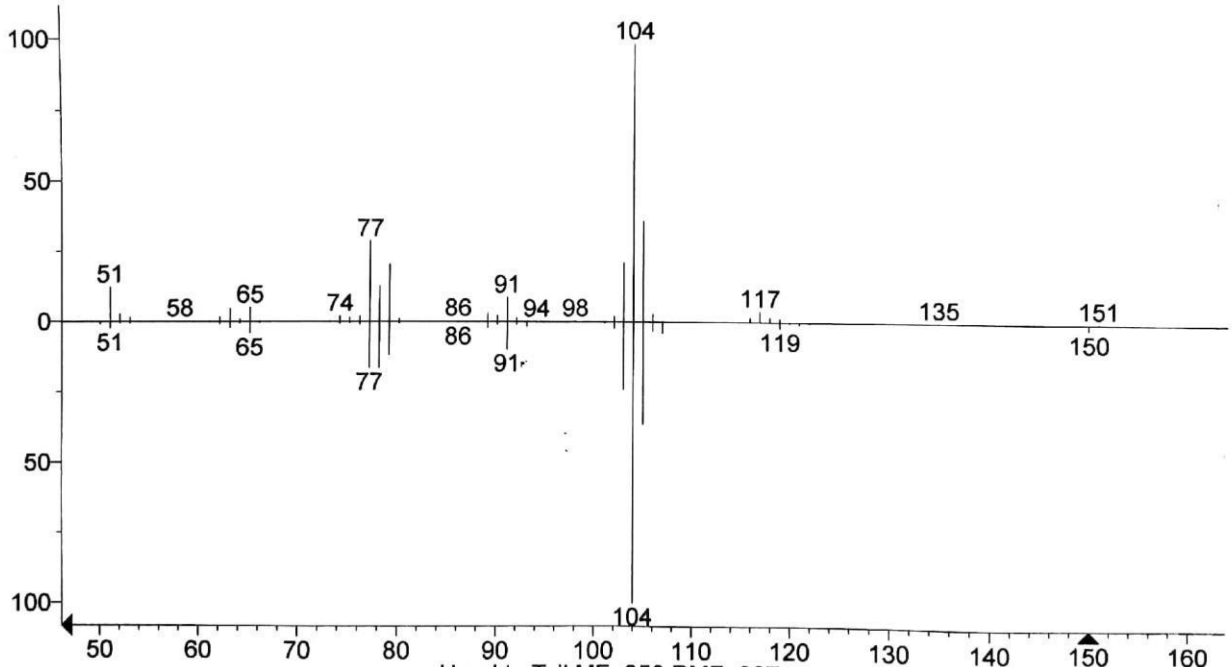

Head to Tail MF=850 RMF=867

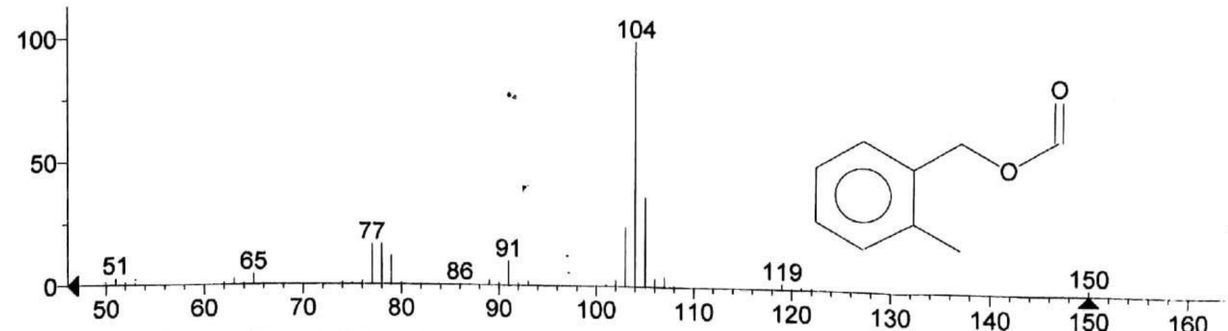

(mainlib) Formic acid, (2-methylphenyl)methyl ester

Scanned by CamScanner

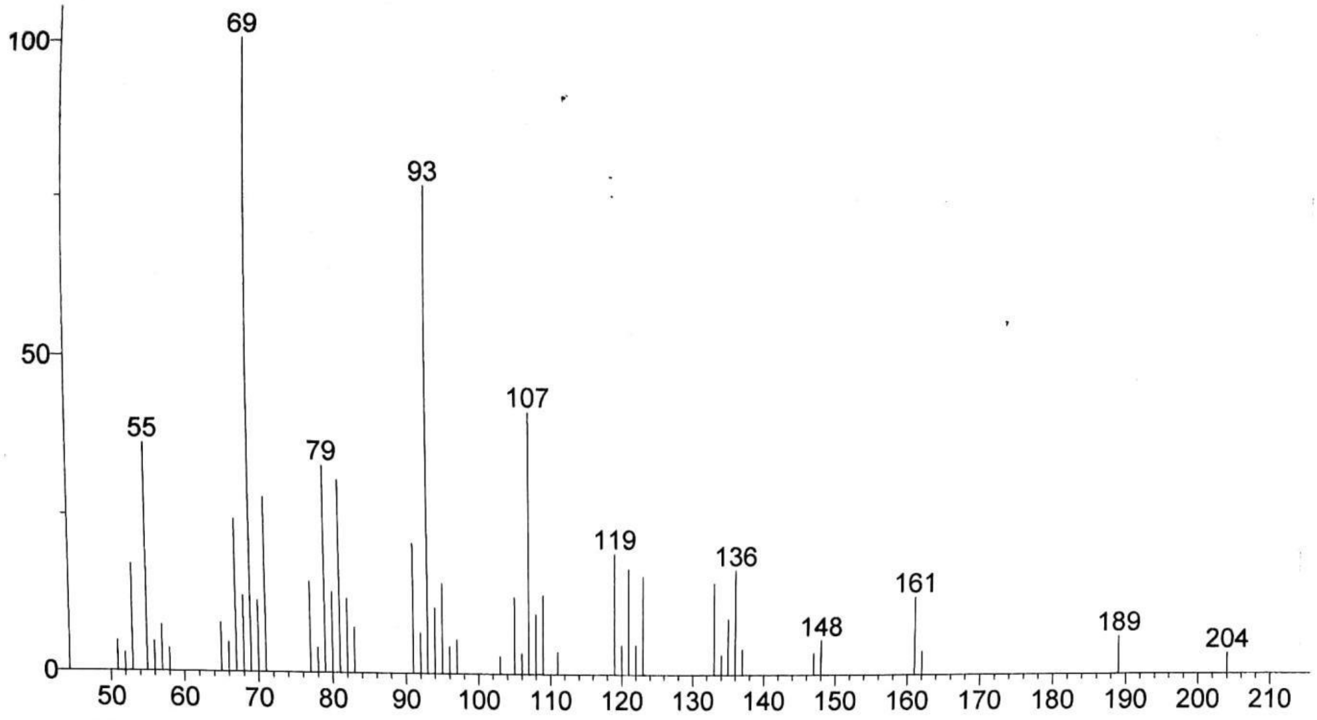

(Text File) Scan 5694 (37.668 min): 02102014A:D\data.ms

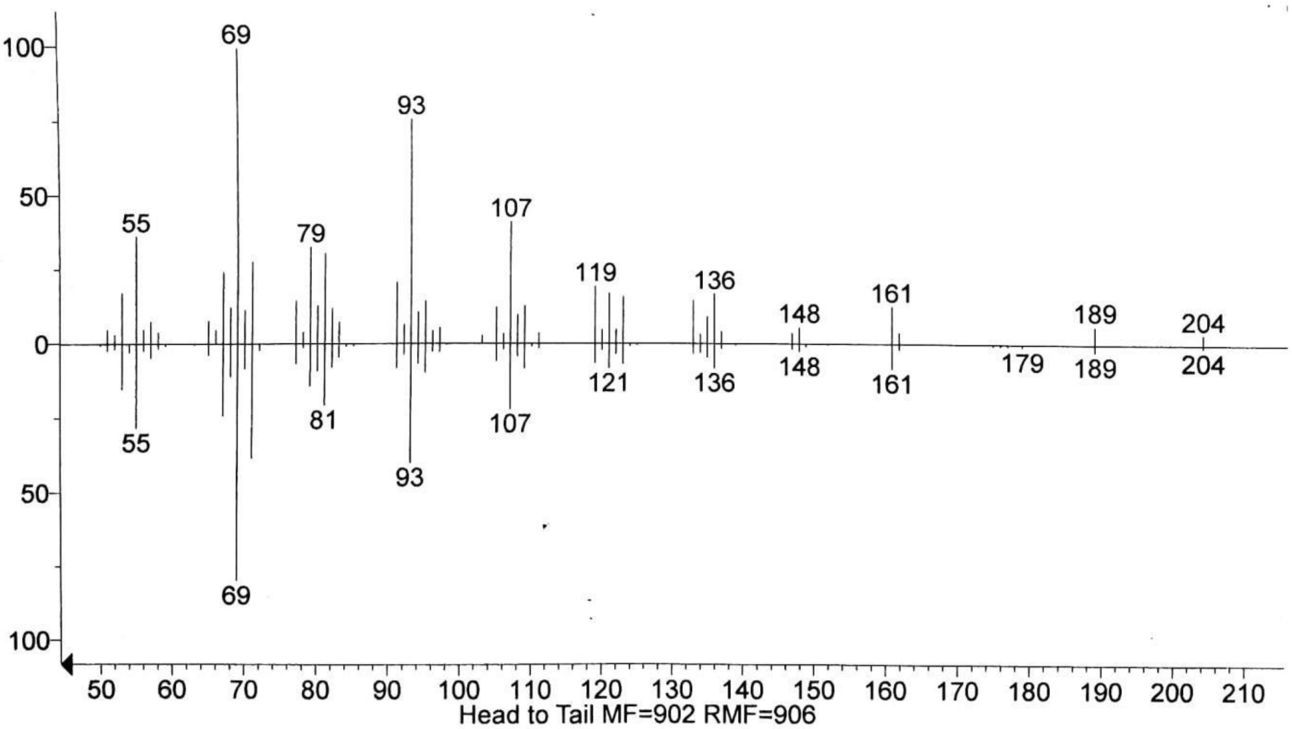

Head to Tail MF=902 RMF=906

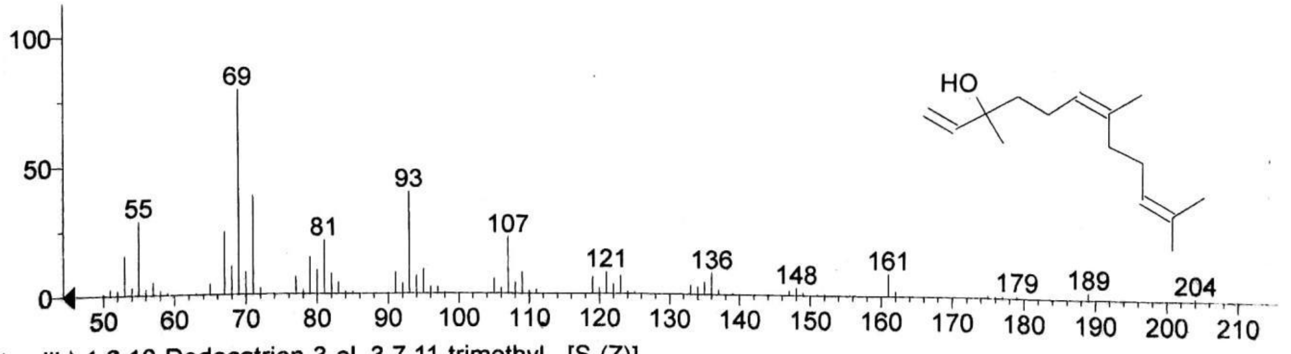

(replib) 1,6,10-Dodecatrien-3-ol, 3,7,11-trimethyl-, [S-(Z)]-

Scanned by CamScanner

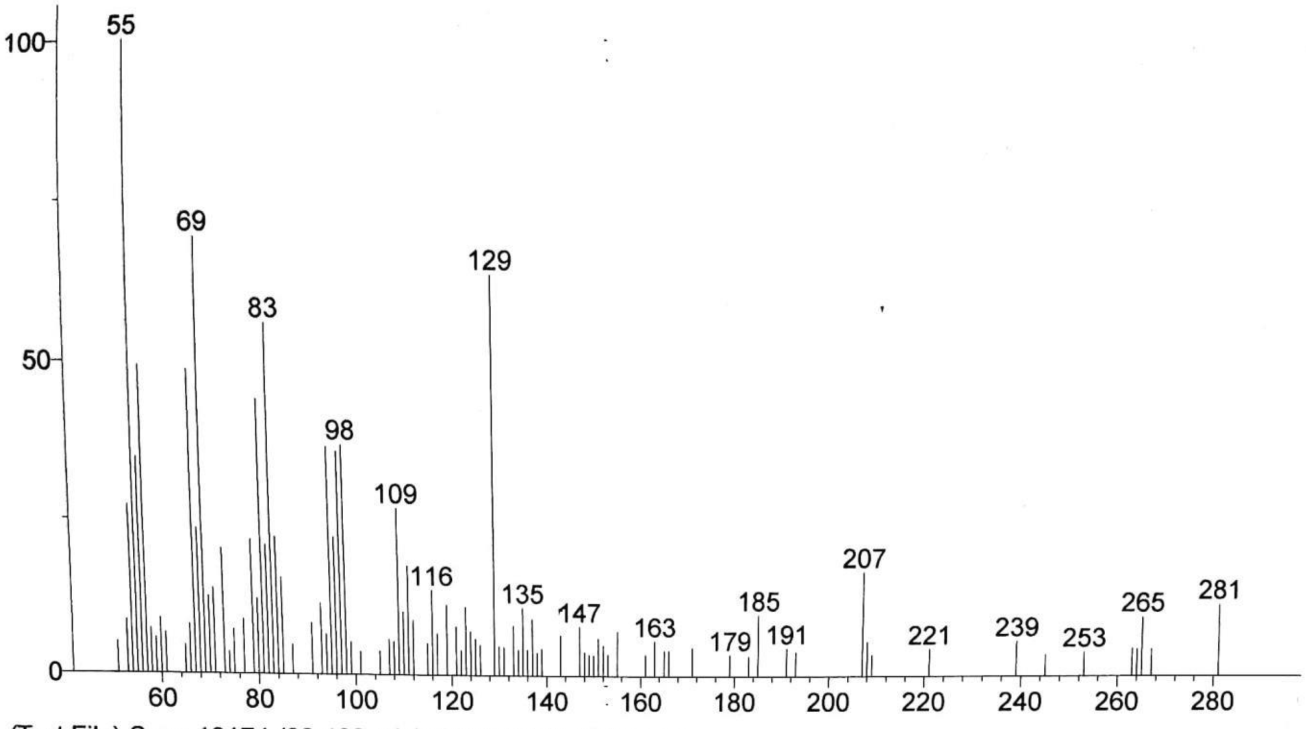

(Text File) Scan 13174 (80.468 min): 02102014A.D\data.ms

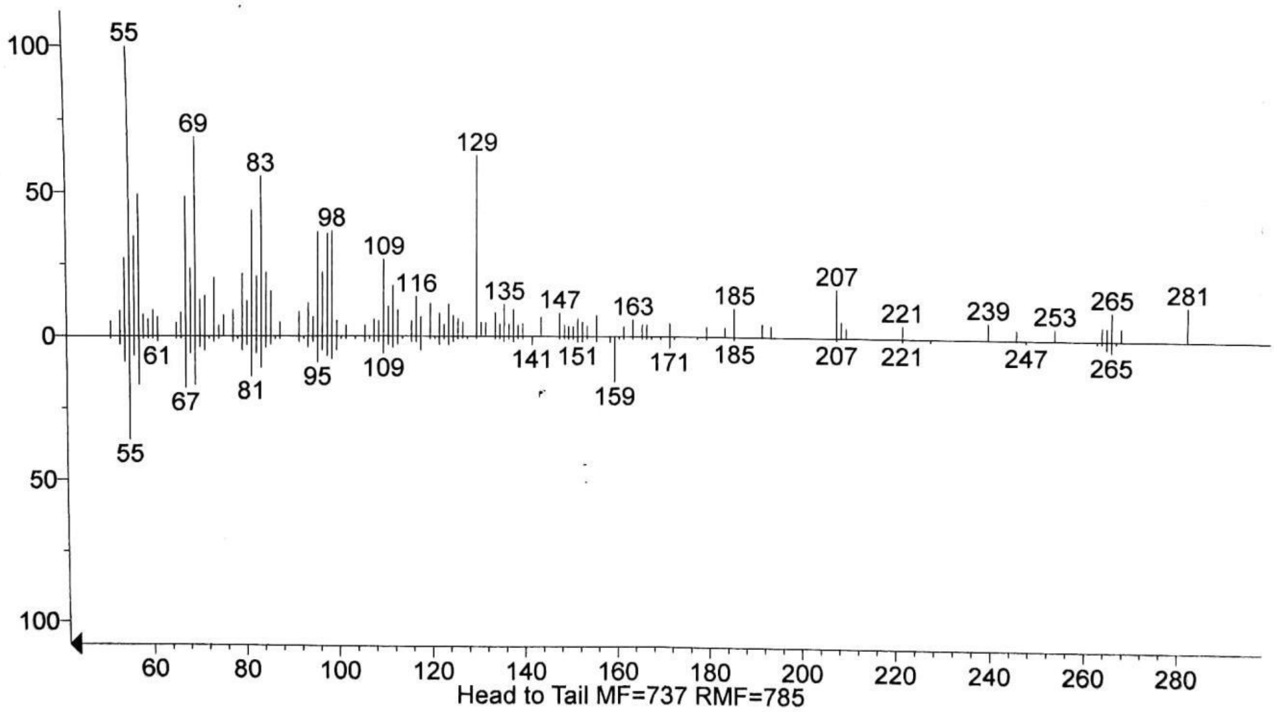

Head to Tail MF=737 RMF=785

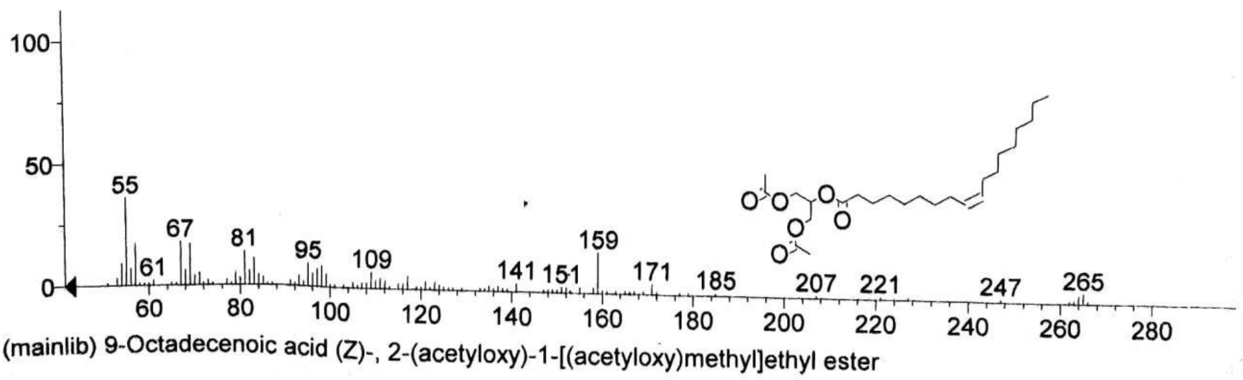

(mainlib) 9-Octadecenoic acid (Z)-, 2-(acetyloxy)-1-[(acetyloxy)methyl]ethyl ester

Scanned by CamScanner

[illegible]strument :
Sample Name: GBENGA PEP CHEM DEPT.
Misc Info :
Vial Number: 1

Dennett

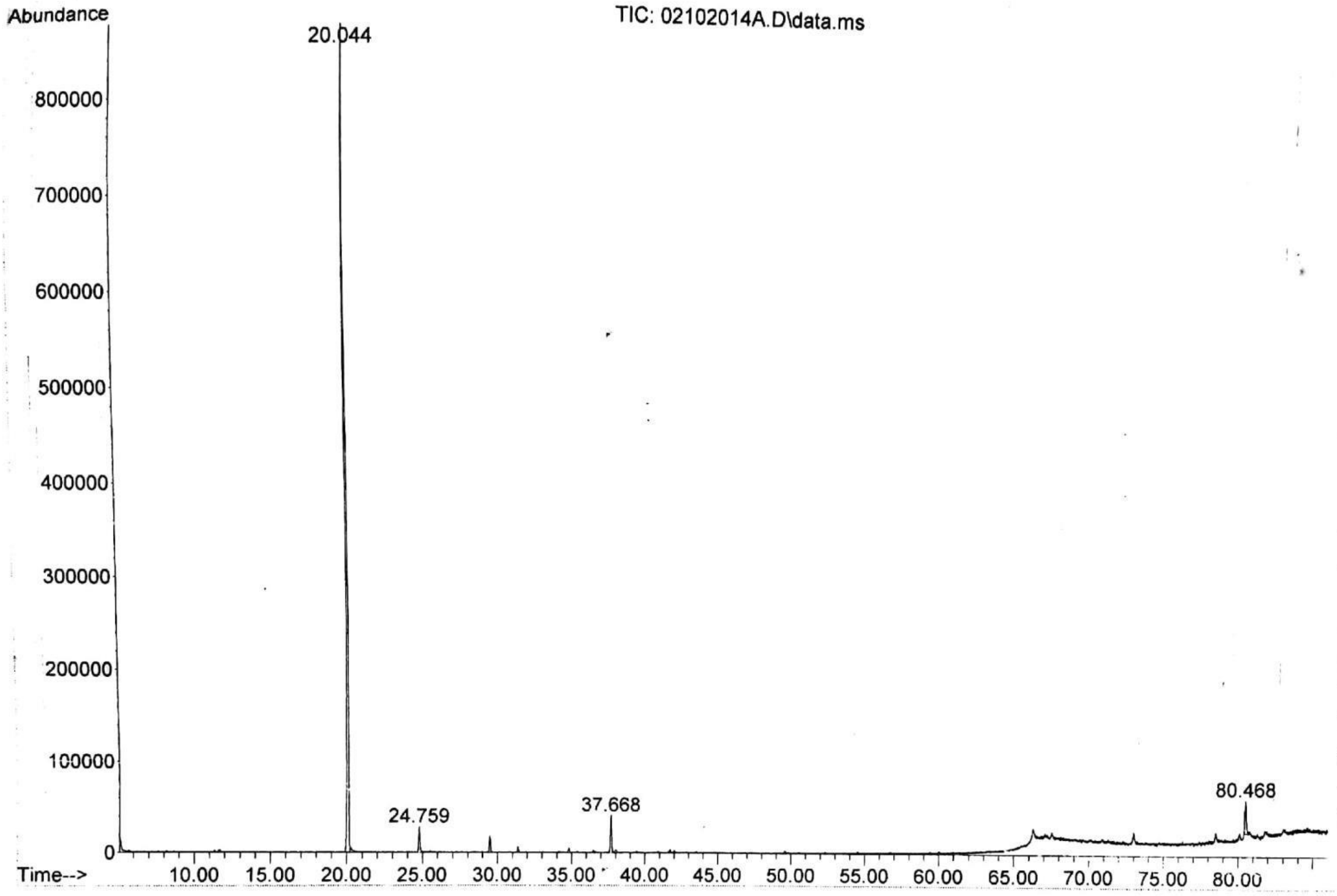

Scanned by CamScanner

Operator
Sample : GBENGA PEP CHEM DEPT.
Misc :
ALS Vial : 1 Sample Multiplier: 1

Search Libraries: C:\Database\NIST11.L Minimum Quality: 0

Unknown Spectrum: Apex
Integration Events: ChemStation Integrator - autoint1.e

Pk#	RT	Area%	Library/ID	Ref#	CAS#	Qual
1	20.044	89.93	C:\Database\NIST11.L			
			Oxalic acid, 2-phenylethyl propyl ester	90433	1000309-65-5	72
			4-Ethylbenzoic acid, 2-phenylethyl ester	105793	1000293-50-1	59
			Acetic acid, trifluoro-, 2-phenyle thyl ester	75324	055419-66-4	50
2	37.668	4.36	C:\Database\NIST11.L			
			Nerolidol 1	79383	1000285-43-5	87
			1,6,10-Dodecatrien-3-ol, 3,7,11-tr imethyl-, (E)-	79437	040716-66-3	83
			Nerolidol 2	79382	1000285-43-6	80
3	80.468	5.71	C:\Database\NIST11.L			
			9-octadecenoic acid, 2,2,2-trifluo roethyl ester	193101	1000376-54-3	53
			1-Cyclohexylnonene	67948	114614-84-5	46
			9-Oxabicyclo[6.1.0]nonane, cis-	11439	004925-71-7	38

Scanned by CamScanner

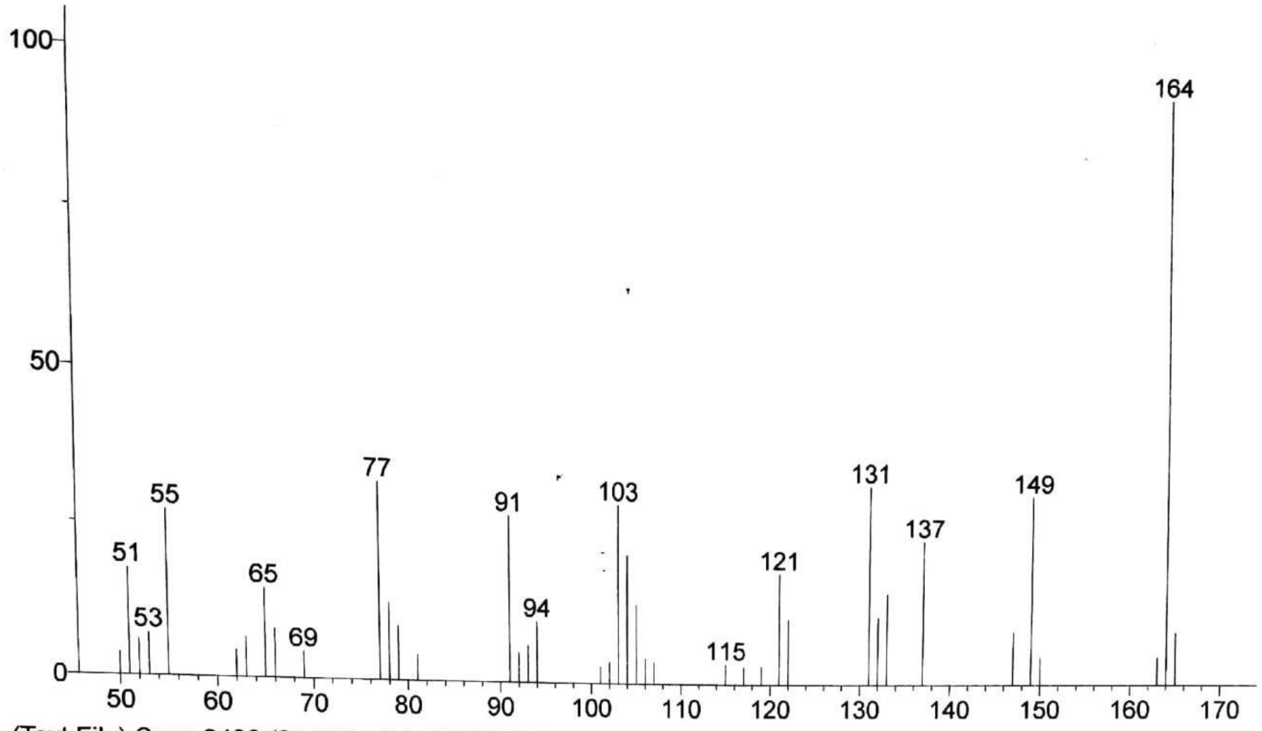

(Text File) Scan 3438 (24.759 min): 02102014A.D\data.ms

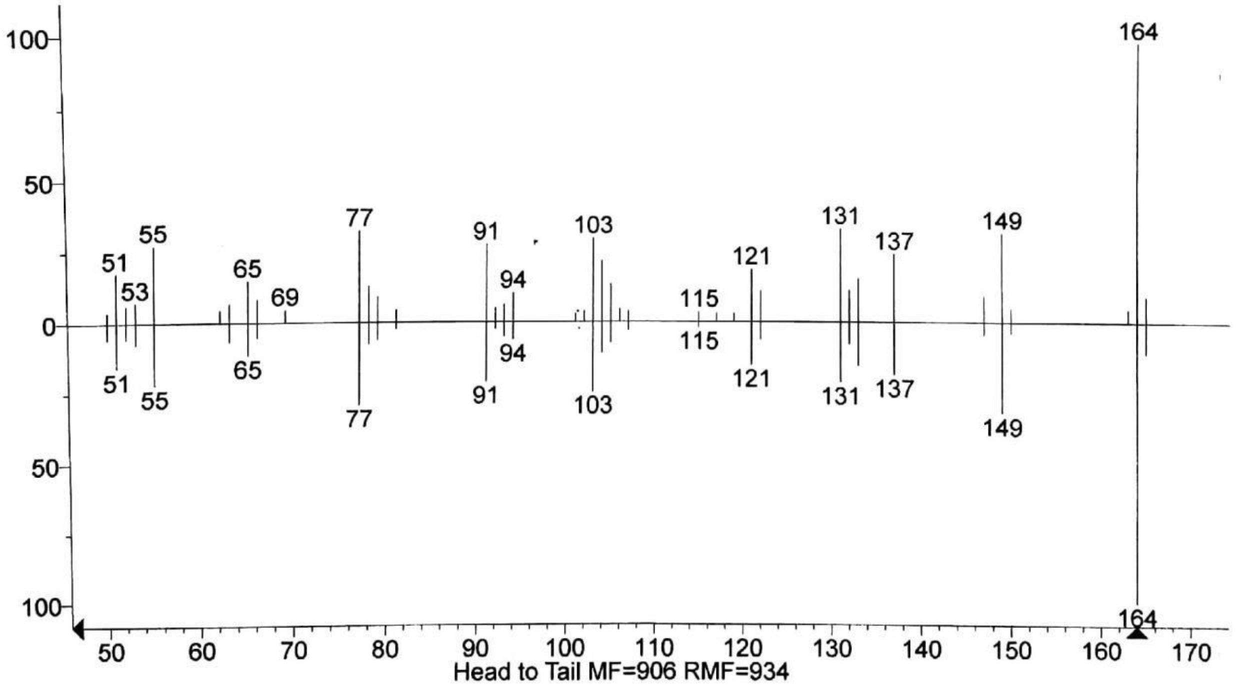

Head to Tail MF=906 RMF=934

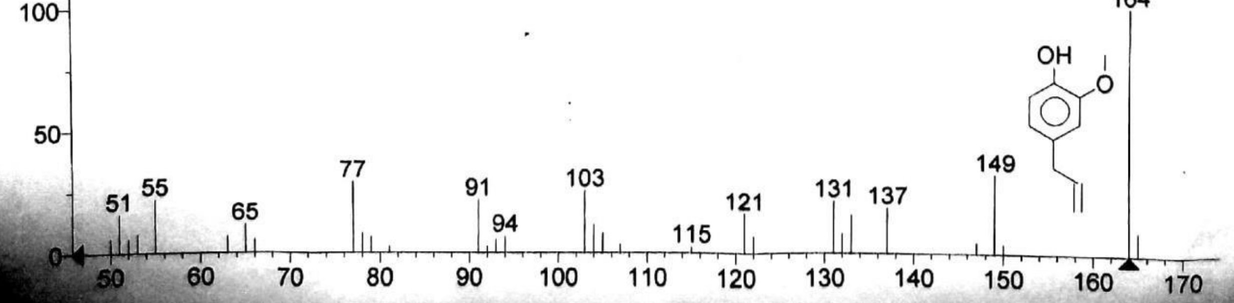

[illegible] Eugenol

Scanned by CamScanner
