# Supplementary material for: Antimicrobial potential of toothpaste formulated from extracts of Syzygium aromaticum, Dennettia tripetala and Jatropha curcas latex against some oral pathogenic microorganisms
Source: AMB Express. 2019 Feb 4;9:20. doi: 10.1186/s13568-019-0744-2 (PMC6362185; doi:10.1186/s13568-019-0744-2)
Supplement: Supplementary file 2 — Additional file 2. The chromatogram of Syzygium aromaticum extract. GCMS revealed the library ID of bioactive compounds in the extract with their peaks, area, retention time, molecular formulae and respective weight. [file 13568_2019_744_MOESM2_ESM.pdf]

Additional file 2

AMB Express

Antimicrobial Potential of Toothpaste formulated from Extracts of *Syzygium aromaticum*, *Dennettia tripetala* and *Jatropha curcas* latex against some Oral Pathogenic Microorganisms

Olugbenga Oludayo Oluwasina (oooluwasina@futa.edu.ng)[1*], Ifunanya Vivian Ezenwosu (oluwasinagbenga@yahoo.com)[1], Clement Olusola Ogidi (clementogidi@yahoo.com) [2,3] and Victor Olusegun Oyetayo (ovonew67@gmail.com)[2]

[1]Department of Chemistry, The Federal University of Technology, PMB 704, Akure, Nigeria

[2]Department of Microbiology, The Federal University of Technology, PMB 704, Akure, Nigeria

[3] Biotechnology Unit, Department of Biological Sciences, Kings University, PMB 555, Odeomu, Nigeria

*****Corresponding author: oooluwasina@futa.edu.ng, +2348107246660

Additional file 2: the chromatogram of *Syzygium aromaticum* extract. GCMS revealed the library ID of bioactive compounds in the extract with their peaks, area, retention time, molecular formulae and respective weight.

Scanned by CamScanner

mple Name: GBENGA CHEM PEP
sc Info :
al Number: 1

Syzgium

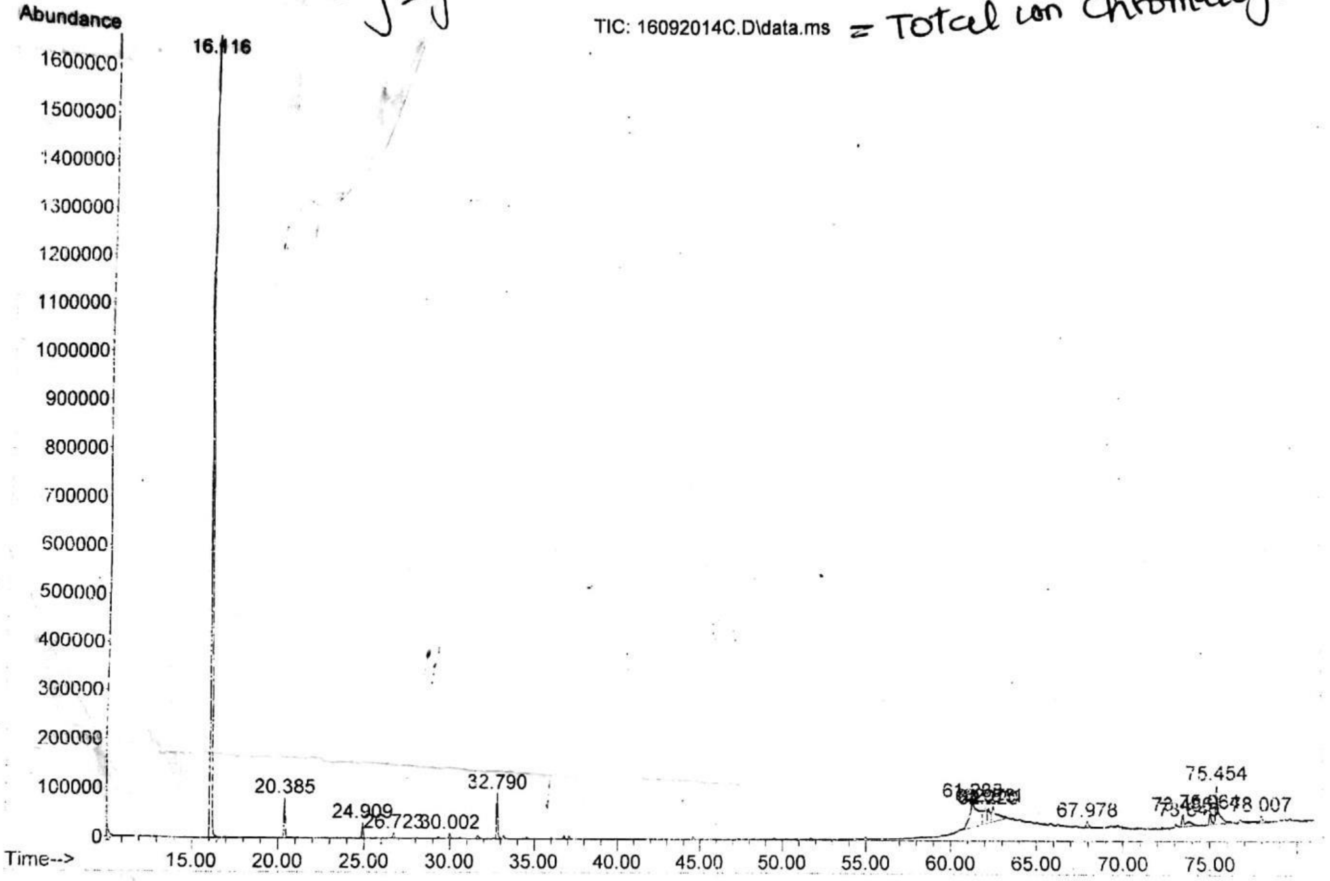

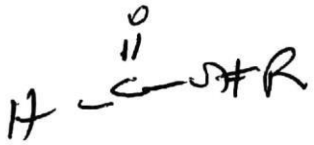

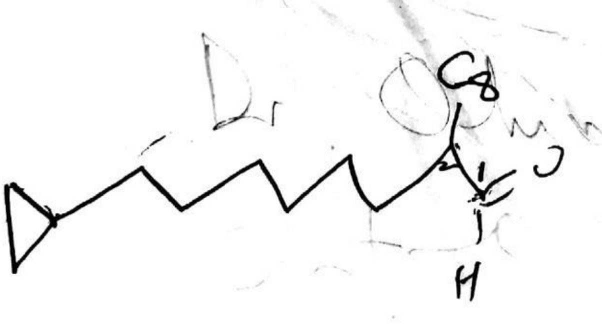

Scanned by CamScanner

Sample : GBENGA CHEM. PEP
Misc :
ALS Vial : 1 Sample Multiplier: 1

Search Libraries: C:\Database\NIST11.L Minimum Quality: 0

Unknown Spectrum: Apex
Integration Events: ChemStation Integrator - autoint1.e

Pk#	RT	Area%	Library/ID	Ref#	CAS#	Qual
1	16.118	57.57	C:\Database\NIST11.L			
			Formic acid, (2-methylphenyl)methyl ester	24532	1000368-93-3	64
			Benzenemethanol, 2-methyl-, acetat	33429	017373-93-2	64
			Glutaric acid, di(phenethyl) ester	176924	1000358-69-4	64
2	20.386	2.90	C:\Database\NIST11.L			
			Phenol, 2-methoxy-3-(2-propenyl)-	33399	001941-12-4	98
			Eugenol	33238	000097-53-0	98
			Eugenol	33241	000097-53-0	98
3	24.907	1.12	C:\Database\NIST11.L			
			Caryophyllene	64272	000087-44-5	99
			Caryophyllene	64275	000087-44-5	96
			Bicyclo[7.2.0]undec-4-ene, 4,11,11-trimethyl-8-methylene-	64418	013877-93-5	93
4	26.721	0.52	C:\Database\NIST11.L			
			1,5-Dimethyl-1-vinyl-4-hexenyl butyrate	81047	000078-36-4	53
			Tricyclo[2.2.1.0(2,6)]heptane, 1,3,3-trimethyl-	15869	000488-97-1	47
			1,6-Octadien-3-ol, 3,7-dimethyl-, formate	47002	000115-99-1	47
5	30.005	0.38	C:\Database\NIST11.L			
			1-Methyltricyclo[2.2.1.0(2,6)]heptane	5508	004601-85-8	49
			Cyclohexane,ethenylidene-	5446	005664-20-0	43
			Tricyclo[2.2.2.0(1,4)]octane	5473	036120-88-4	38
6	32.792	3.36	C:\Database\NIST11.L			
			1,6,10-Dodecatrien-3-ol, 3,7,11-trimethyl-, (E)-	79437	040716-66-3	91
			Nerolidol 2	79382	1000285-43-6	91
			1,6,10-Dodecatrien-3-ol, 3,7,11-trimethyl-	79426	007212-44-4	90
7	61.287	11.80	C:\Database\NIST11.L			
			9,12-Octadecadienoic acid (Z,Z)-	127649	000060-33-3	95
			9,12-Octadecadienoic acid (Z,Z)-	127647	000060-33-3	89
			Cyclopropaneoctanal, 2-octyl-	127746	056196-06-6	68
8	62.071	3.30	C:\Database\NIST11.L			
			2-Methyl-Z,Z-3,13-octadecadienol	127747	1000130-90-5	94
			9,12-Octadecadienoic acid (Z,Z)-	127647	000060-33-3	93
			9,12-Octadecadienoic acid (Z,Z)-	127649	000060-33-3	90
9	62.220	2.20	C:\Database\NIST11.L			
			2-Methyl-Z,Z-3,13-octadecadienol	127747	1000130-90-5	93
			Octadec-9-enoic acid	129341	1000190-13-7	90
			9,12-Octadecadienoic acid (Z,Z)-	127647	000060-33-3	70
10	62.512	4.39	C:\Database\NIST11.L			
			Cyclopropaneoctanal, 2-octyl-	127746	056196-06-6	[illegible]
			9-Octadecenoic acid (Z)-, 2,3-dihy	[illegible]	[illegible]	[illegible]

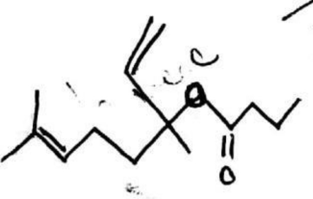

Scanned by CamScanner

Pk#	RT	Area%	Library/ID	Ref#	CAS#	Qual
			droxypropyl ester	127768	000296-56-0	81
			Cycloeicosane			
11	67.976	0.80	C:\Database\NIST11.L			
			Cyclododecane	36738	000294-62-2	53
			1,19-Eicosadiene	126193	014811-95-1	46
			Cyclododecane	36737	000294-62-2	41
12	73.486	1.21	C:\Database\NIST11.L			
			9-Octadecenal, (Z)-	115866	002423-10-1	93
			9-Octadecenoic acid (Z)-, 2-hydroxyethyl ester	166116	004500-01-0	62
			9-Octadecenoic acid (Z)-, 2-hydroxy-1-(hydroxymethyl)ethyl ester	188149	003443-84-3	58
13	73.841	1.49	C:\Database\NIST11.L			
			cis-9-Hexadecenal	92517	056219-04-6	30
			9-Octadecenal, (Z)-	115866	002423-10-1	15
			Trichloroacetic acid, undec-2-enyl ester	155835	1000299-26-1	15
14	75.066	1.18	C:\Database\NIST11.L			
			2(1H)-Naphthalenone, octahydro-4a-methyl-7-(1-methylethyl)-, (4a.alpha.,7.beta.,8a.beta.)-	67892	054594-42-2	92
			1,3,12-Nonadecatriene	112661	1000131-11-1	87
			cis,cis-7,10,-Hexadecadienal	90904	056829-23-3	62
15	75.455	6.69	C:\Database\NIST11.L			
			9-Octadecenal, (Z)-	115866	002423-10-1	90
			2-Methyl-Z,Z-3,13-octadecadienol	127747	1000130-90-5	83
			9,12-Octadecadienoic acid (Z,Z)-	127647	000060-33-3	70
16	78.007	1.10	C:\Database\NIST11.L			
			Phthalic acid, cylclohexylmethyl pentyl ester	170888	1000309-07-5	43
			Di(Z)-hex-3-enyl phthalate	169298	1000373-65-0	38
			Di(E)-hex-3-enyl phthalate	169297	1000373-64-8	38

Path
File
Acq On
Operator
Sample

Scanned by CamScanner

Sample : GBENGA CHEM. PEP
Misc :
ALS Vial : 1 Sample Multiplier: 1

Integration Parameters: events.e
Integrator: ChemStation

Method : C:\MSDCHEM\1\METHODS\IBITOYE METHOD\IBITOYE METHOD.M
Title :

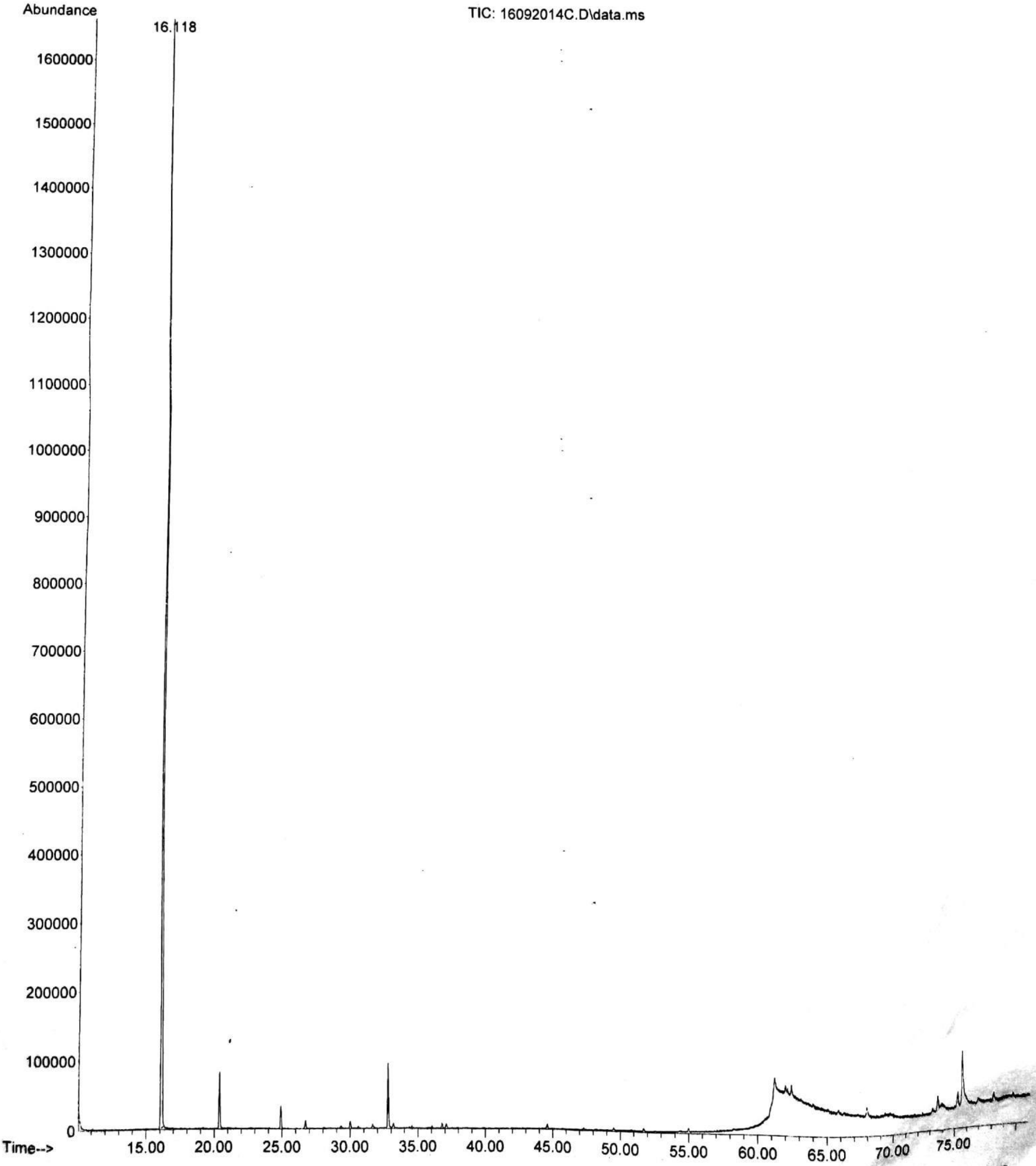

Scanned by CamScanner

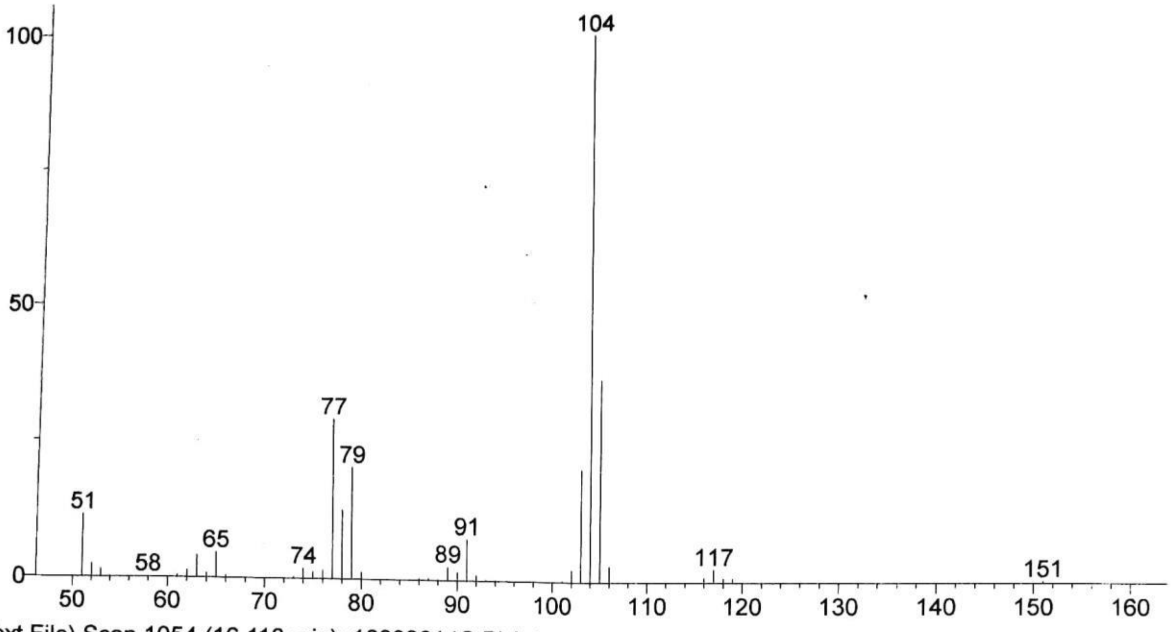

(Text File) Scan 1054 (16.118 min): 16092014C.D\data.ms

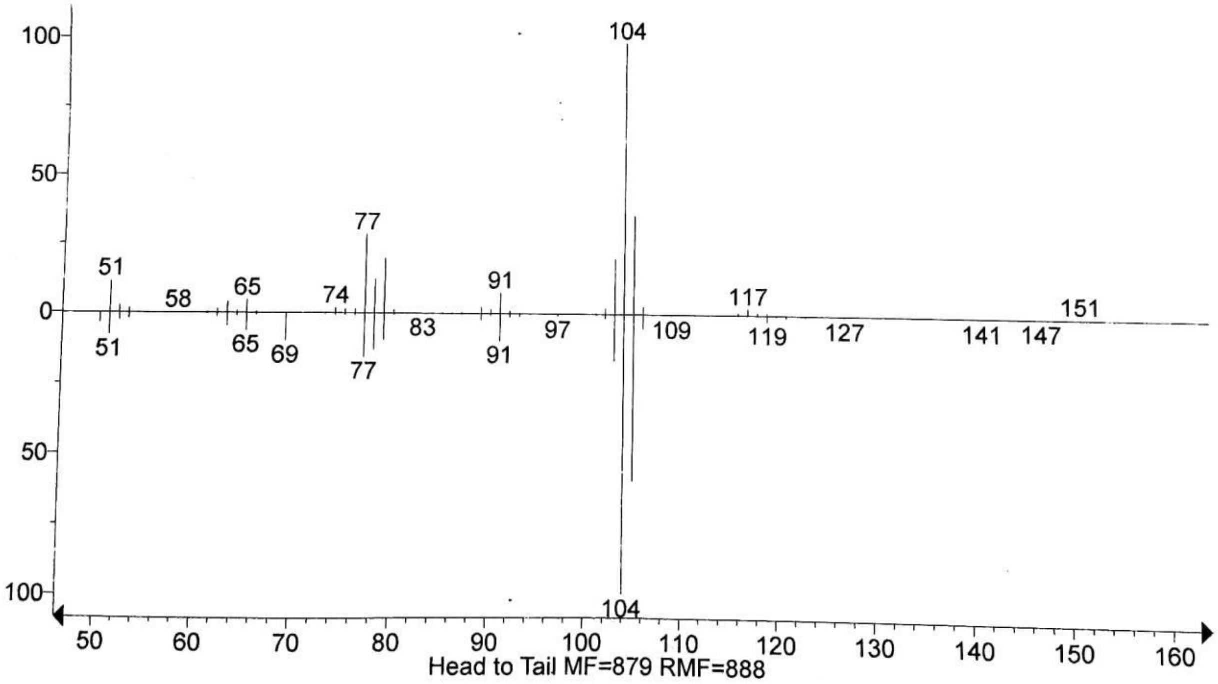

Head to Tail MF=879 RMF=888

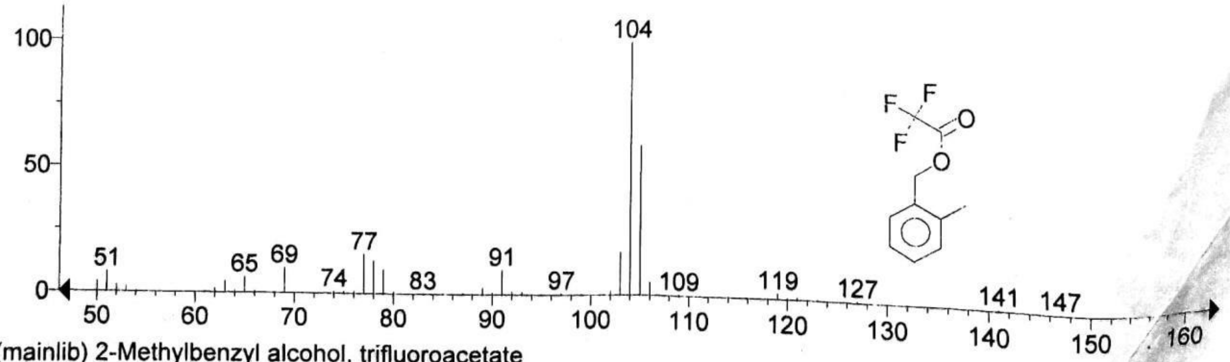

(mainlib) 2-Methylbenzyl alcohol, trifluoroacetate

Scanned by CamScanner

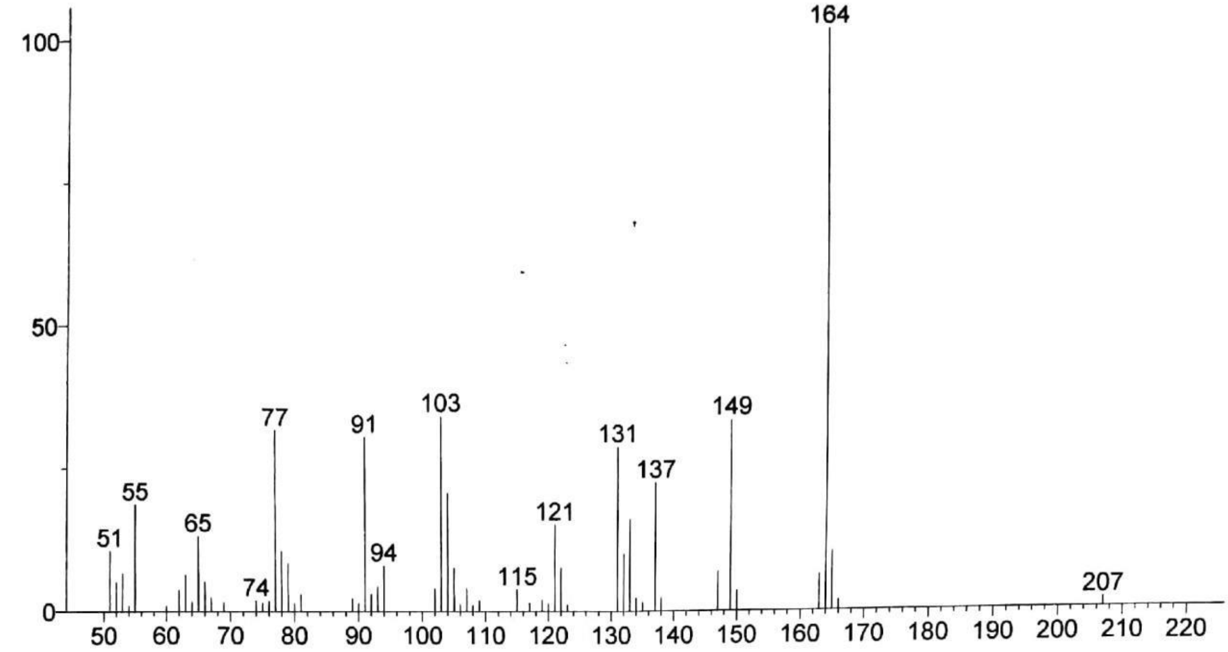

(Text File) Scan 1800 (20.386 min): 16092014C.D\data.ms

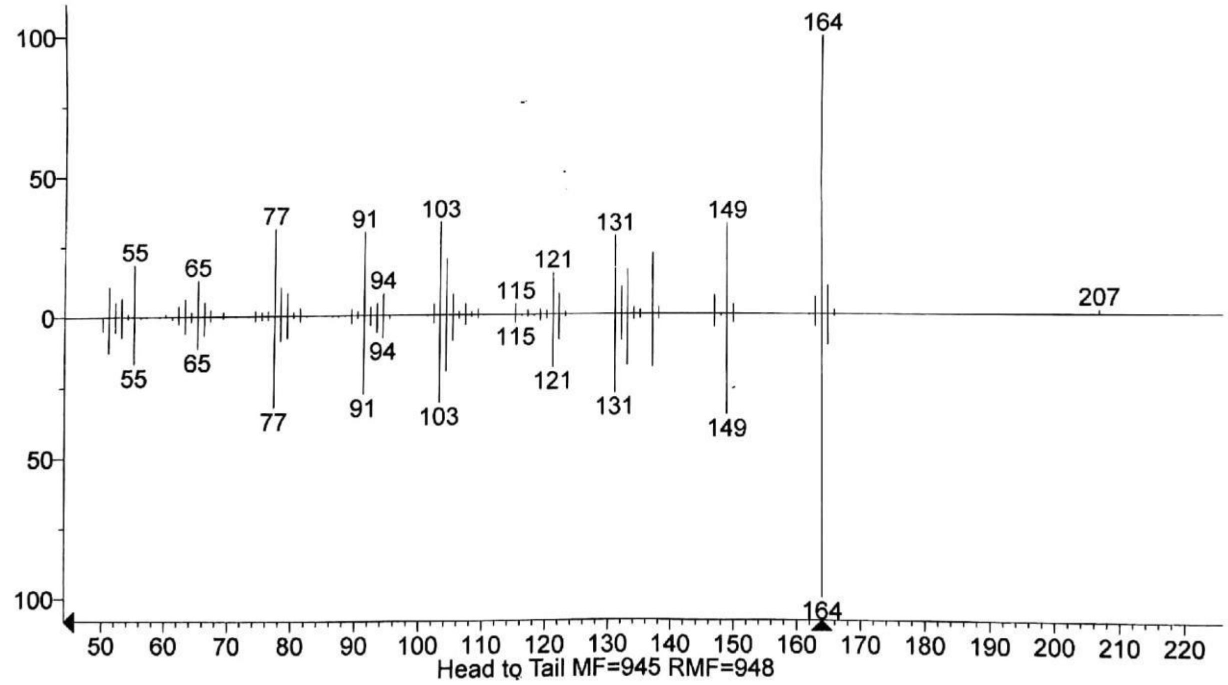

Head to Tail MF=945 RMF=948

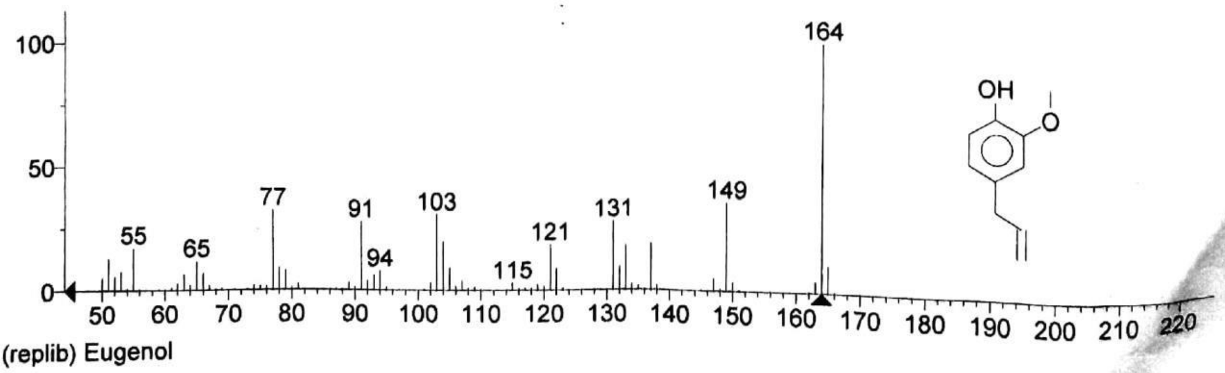

(replib) Eugenol

Scanned by CamScanner

Name: Eugenol
Formula: $C_{10}H_{12}O_2$
MW: 164 Exact Mass: 164.08373 CAS#: 97-53-0 NIST#: 378793 ID#: 23453 DB: replib
Other DBs: Fine, TSCA, RTECS, EPA, USP, HODOC, NIH, EINECS, IRDB
Contributor: United States Army Criminal Investigation Laboratory (USACIL)
10 largest peaks:

164 999	149 358	77 325	103 312	131 281
91 279	104 201	137 189	121 188	133 183

Synonyms:
1.Phenol, 2-methoxy-4-(2-propenyl)-
2.Phenol, 4-allyl-2-methoxy-
3.p-Allylguaiacol
4.p-Eugenol
5.Caryophyllic acid
6.Engenol
7.Eugenic acid
8.2-Methoxy-1-hydroxy-4-allylbenzene
9.2-Methoxy-4-allylphenol
10.4-Allyl-2-methoxyphenol
11.4-Allylguaiacol
12.4-Hydroxy-3-methoxyallylbenzene
13.NCI-C50453
14.1-Hydroxy-2-methoxy-4-allylbenzene
15.1-Hydroxy-2-methoxy-4-prop-2-enylbenzene
16.2-Methoxy-4-(2-propenyl)phenol
17.2-Methoxy-4-prop-2-enylphenol
18.4-Allyl-1-hydroxy-2-methoxybenzene
19.4-Allylcatechol-2-methyl ether
20.1,3,4-Eugenol
21.FA 100
22.FEMA No. 2467
23.2-Metoksy-4-allilofenol
24.2-Hydroxy-5-allylanisole
25.Allylguaiacol
26.4-(2-Propenyl)-2-methoxyphenol
27.NSC 209525
28.Phenol, 2-methoxy-4-(2-propen-1-yl)-

Estimated non-polar retention index (n-alkane scale):
Value: 1392 iu
Confidence interval (Diverse functional groups): 89(50%) 382(95%) iu

Retention index.
1. Value: 1337 iu
Column Type: Capillary
Column Class: Standard non-polar
Active Phase: BP-1
Column
Length: 50 m
Carrier Gas: He
Column Diameter: 0.22 mm
Phase Thickness: 0.25 um
Data Type: Linear
RI
Program Type: Ramp
Start T: 60 C
End T: 220 C
Heat Rate: 2 K/min

Scanned by CamScanner

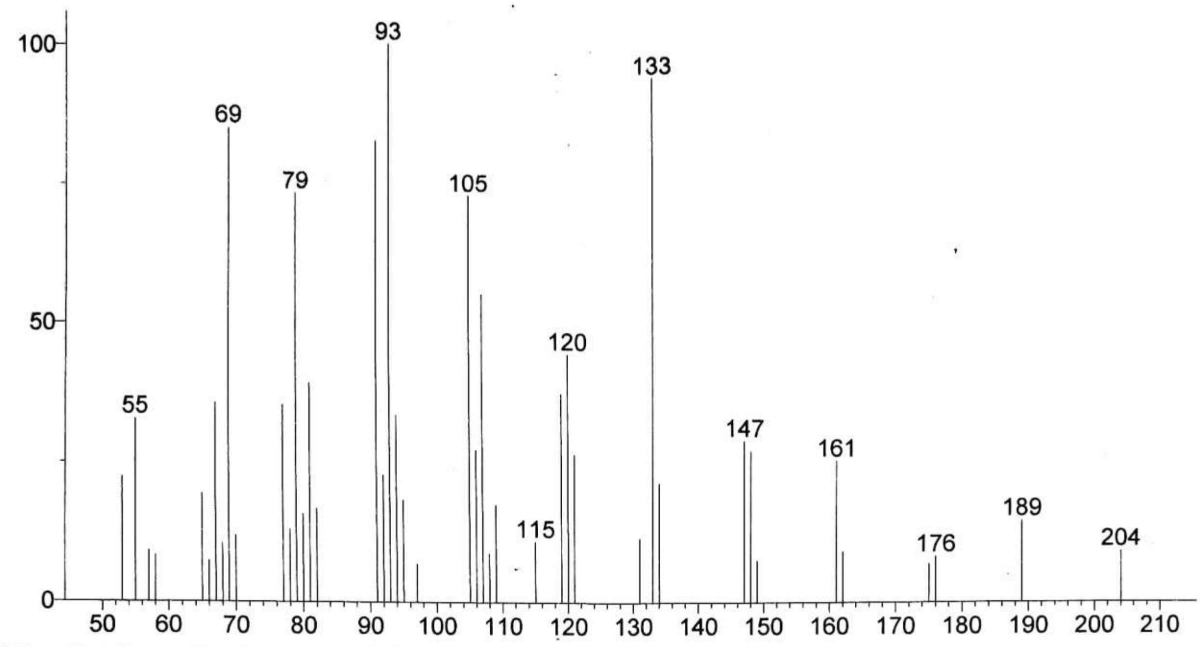

(Text File) Scan 2590 (24.907 min): 16092014C.D\data.ms

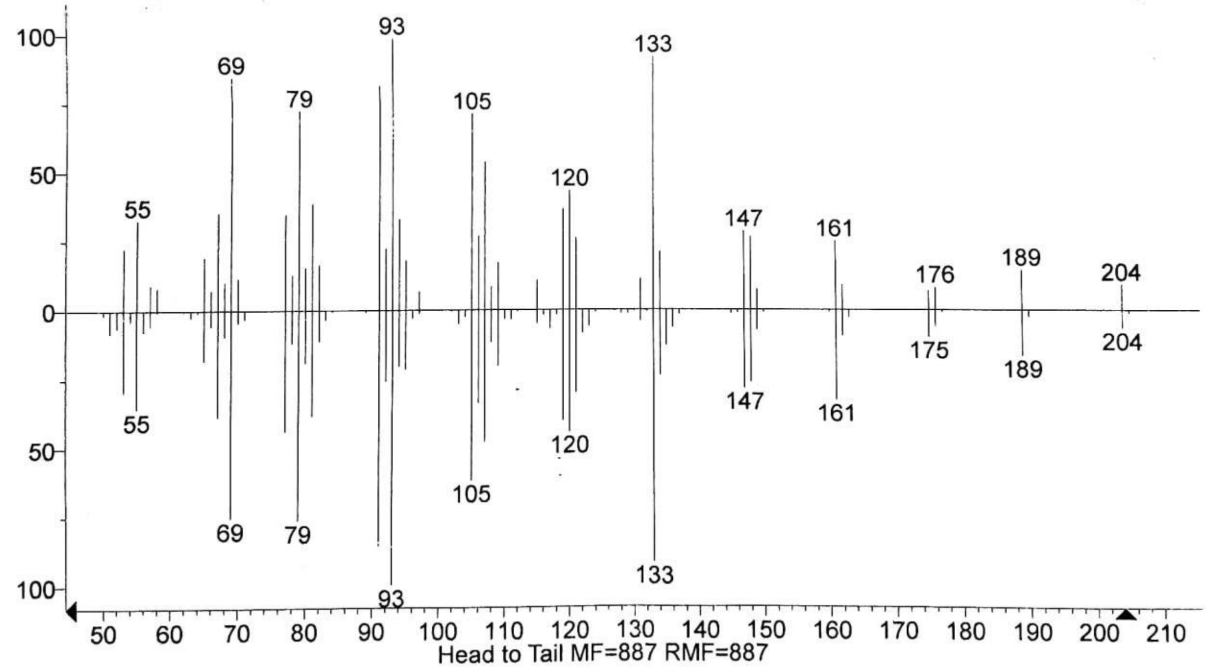

Head to Tail MF=887 RMF=887

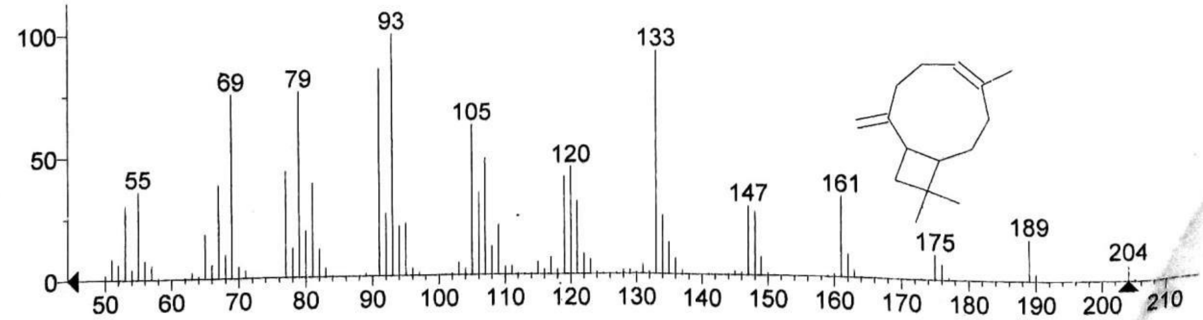

(mainlib) Caryophyllene

Scanned by CamScanner

Name: Caryophyllene
Formula: $C_{15}H_{24}$
MW: 204 Exact Mass: 204.1878 CAS#: 87-44-5 NIST#: 291486 ID#: 60555 DB: mainlib
Other DBs: Fine, TSCA, RTECS, HODOC, NIH, EINECS
Contributor: NIST Mass Spectrometry Data Center, 1998.
Related CAS#: 8007-38-3; 1407-53-0
10 largest peaks:

93 999 |	133 921 |	91 858 |	41 769 |	79 763 |
69 754 |	105 623 |	107 483 |	120 447 |	77 439 |

Synonyms:
1.Bicyclo[7.2.0]undec-4-ene, 4,11,11-trimethyl-8-methylene-, [1R-(1R*,4E,9S*)]-
2.Bicyclo[7.2.0]undec-4-ene, 4,11,11-trimethyl-8-methylene-, (E)-(1R,9S)-(-)-
3.β-Caryophyllen
4.β-Caryophyllene
5.trans-Caryophyllene
6.L-Caryophyllene
7.Bicyclo(7.2.0)undec-4-ene, 8-methylene-4,11,11-trimethyl-, (E)-(1R,9S)-(-)-
8.8-Methylene-4,11,11-(trimethyl)bicyclo(7.2.0)undec-4-ene, (1R,4E,9S)-
9.beta-Caryophyllene
10.β-(E)-Caryophyllene
11.β-trans-Caryophyllene
12.Caryophyllene, (E)
13.E-β-Caryophyllene
14.(E)-Caryophyllene
15.trans-β-Caryophyllene
16.(-)-(E)-Caryophyllene
17.Caryophyllene B
18.NSC 11906

Estimated non-polar retention index (n-alkane scale):
Value: 1494 iu
Confidence interval (Hydrocarbons): 39(50%) 167(95%) iu

Retention index.
1. Value: 1424 iu
Column Type: Capillary
Column Class: Standard non-polar
Active Phase: RTX-1
Column
Length: 60 m
Carrier Gas: He
Column Diameter: 0.22 mm
Phase Thickness: 0.25 um
Data Type: Linear
RI
Program Type: Ramp
Start T: 60 C
End T: 230 C
Heat Rate: 2 K/min
End Time: 30 min
Source:
Bendahou, M.; Muselli, A.; Grignon-Dubois, M.; Benyoucef, M.; Desjobert, J.-M.; Bernardini, A.-F.; Costa, J., Antimicrobial activity and chemical composition of Origanum glandulosum Desf. essential oil and extract obtained by microwave extraction: Comparison with hydrodistillation, Food Chem., 106, 2008, 132-139.

2.
Value: 1421 iu
Column Type: Capillary

Scanned by CamScanner

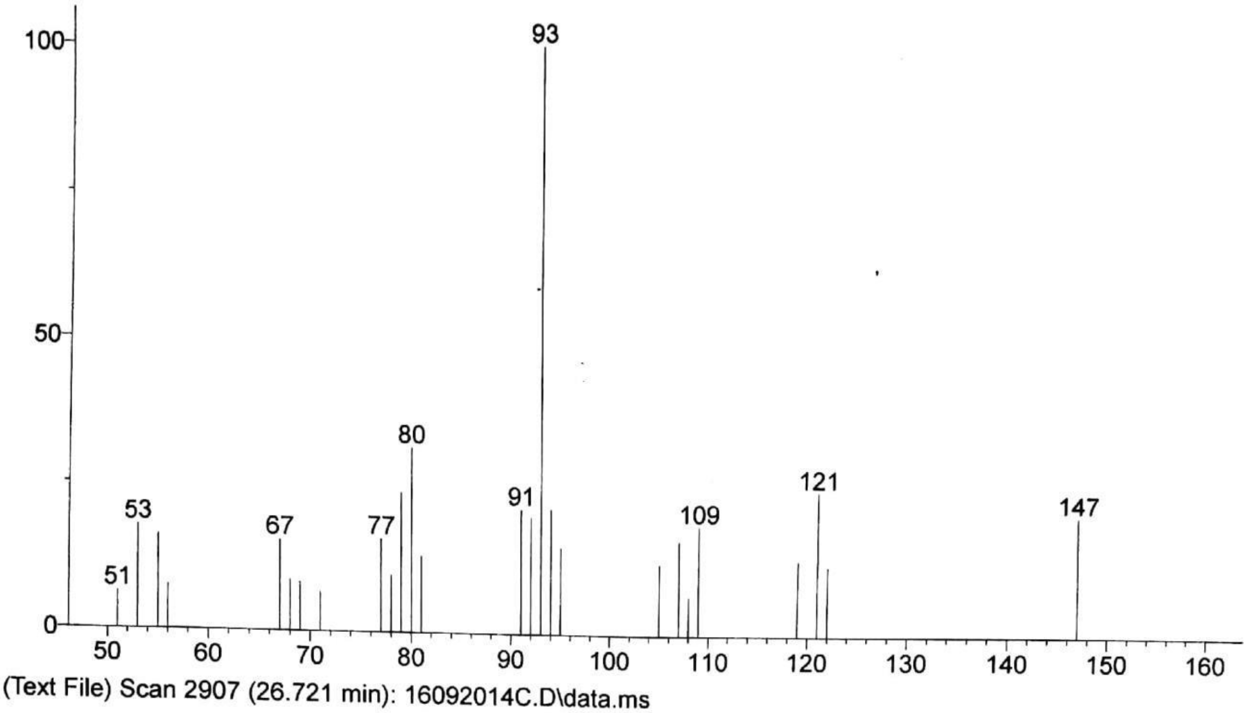

(Text File) Scan 2907 (26.721 min): 16092014C.D\data.ms

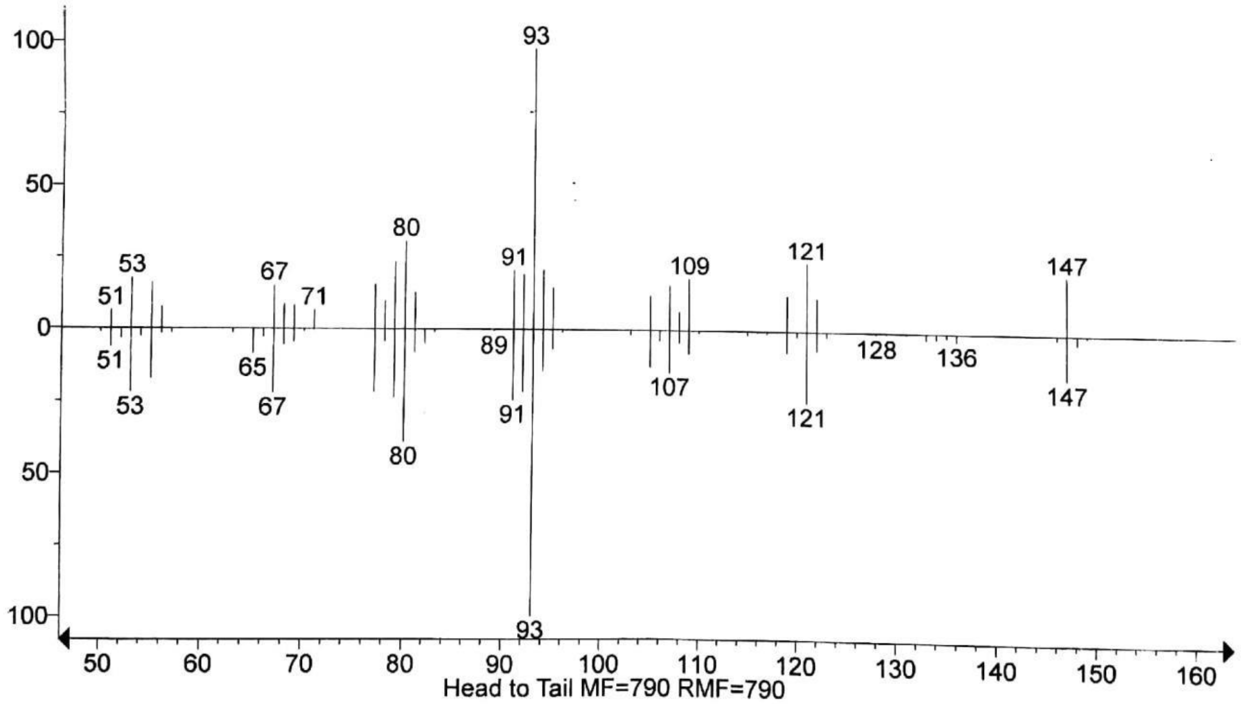

Head to Tail MF=790 RMF=790

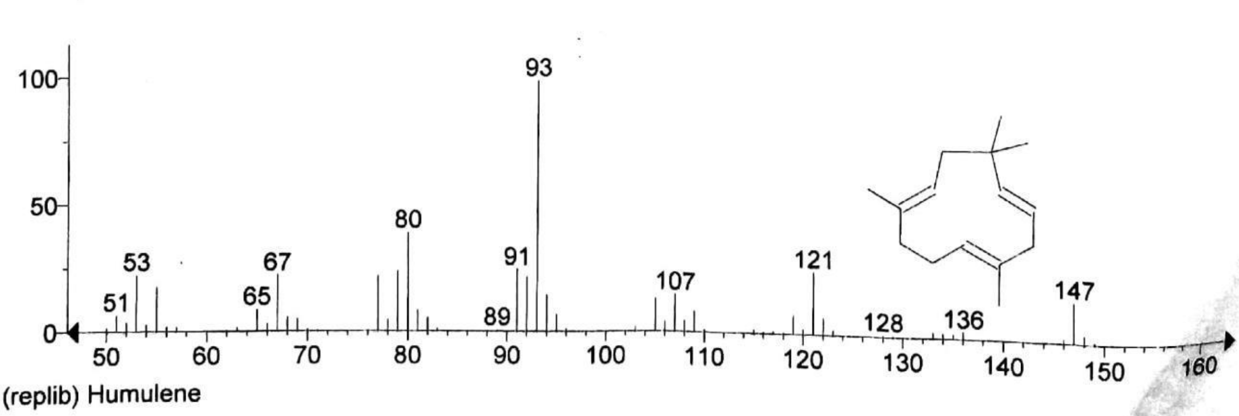

(replib) Humulene

Scanned by CamScanner

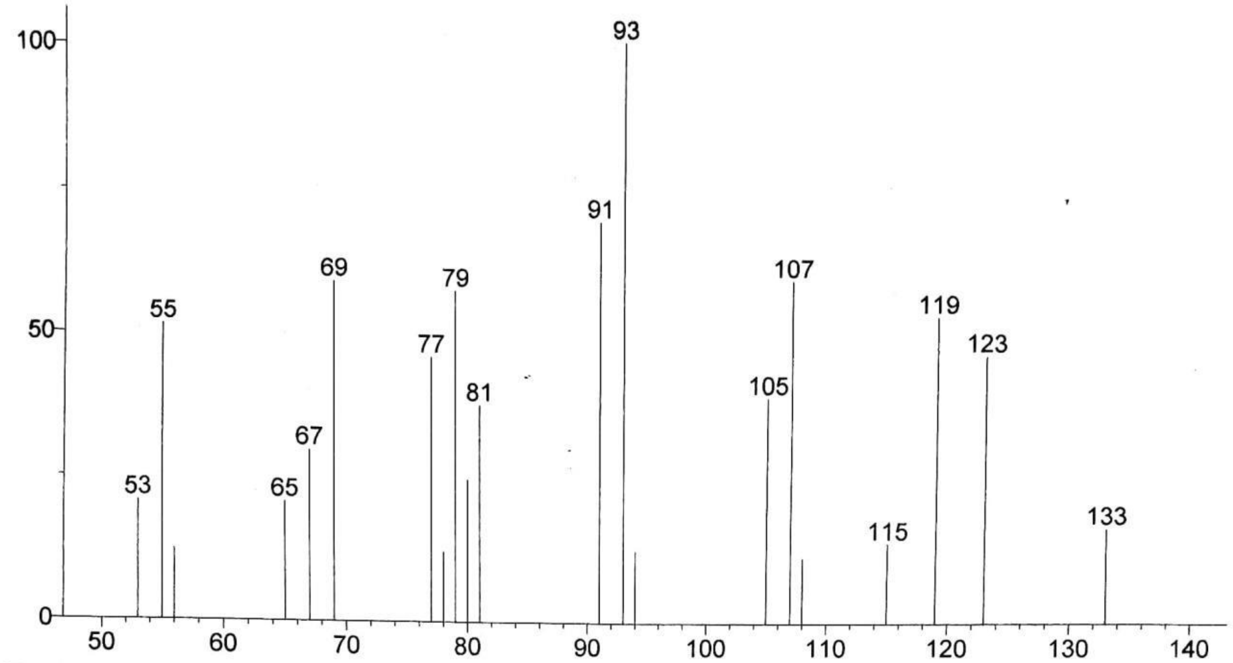

(Text File) Scan 3481 (30.005 min): 16092014C.D\data.ms

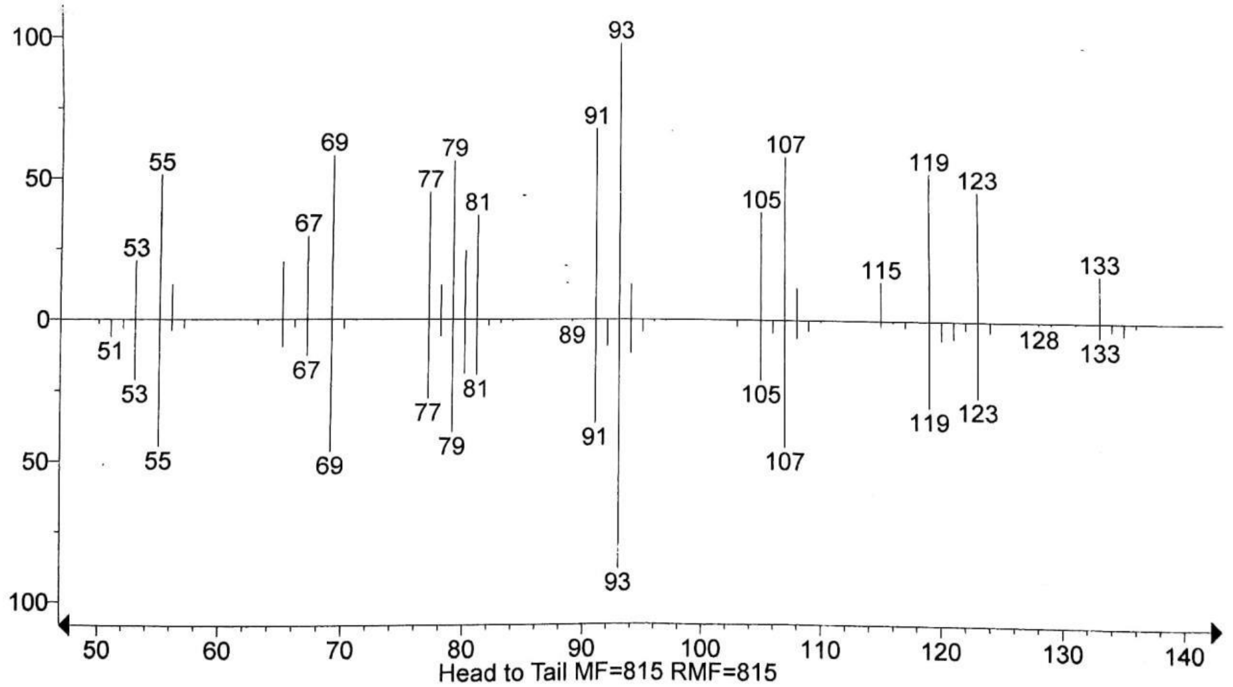

Head to Tail MF=815 RMF=815

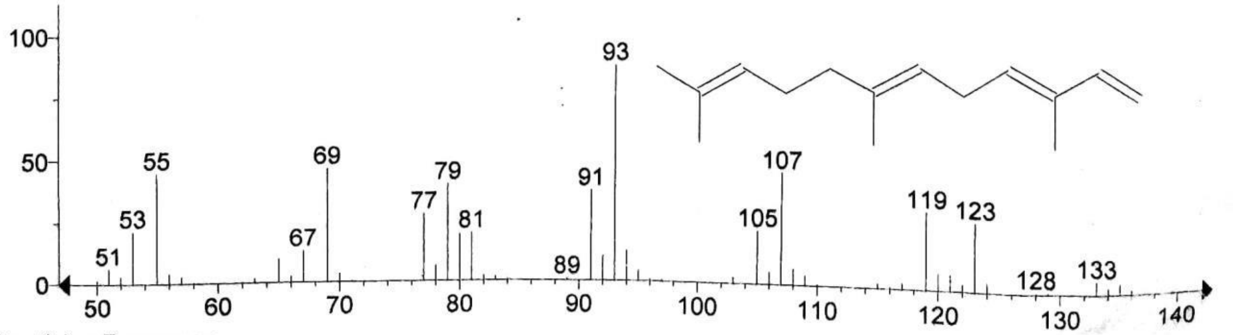

(replib) α-Farnesene

Scanned by CamScanner

Name: α-Farnesene
Formula: $C_{15}H_{24}$
MW: 204 Exact Mass: 204.1878 CAS#: 502-61-4 NIST#: 141113 ID#: 1267 DB: replib
Other DBs: Fine, TSCA, HODOC, EINECS
Contributor: Mark Whitten, Florida Museum of Natural History, U. of Florida
Related CAS#: 18452-58-9; 21499-64-9
10 largest peaks:

41 999	93 885	69 467	107 452	55 449
79 401	91 371	119 308	77 280	123 271

Synonyms:
1.1,3,6,10-Dodecatetraene, 3,7,11-trimethyl-, (E,E)-
2.Farnesene
3.2,6,10-Trimethyl-2,6,9,11-dodecatetraene, trans-
4.3,7,11-Trimethyl-1,3,6,10-dodecatetraene, (trans,trans)-
5.(3E,6E)-3,7,11-Trimethyl-1,3,6,10-dodecatetraene #
6.α-E,E-Farnesene
7.α-Farnesene, (E,E)-
8.α-trans-Farnesene
9.α-trans,trans-Farnesene
10.(3E,6E)- α-Farnesene
11.(E,E)-α-Farnesene
12.alpha-Farnesene
13.trans-α-Farnesene
14.trans,trans-α-Farnesene
15.1,3,6,10-Dodecatetraene, 3,7,11-trimethyl-, (3E,6E)-
16.trans-2,6,10-Trimethyl-2,6,9,11-dodecatetraene
17.trans-3,7,11-Trimethyl-1,3,6,10-dodecatetraene

Estimated non-polar retention index (n-alkane scale):
Value: 1458 iu
Confidence interval (Hydrocarbons): 39(50%) 167(95%) iu

Retention index.
1. Value: 1499 iu
Column Type: Capillary
Column Class: Standard non-polar
Active Phase: BP-1
Column
Length: 50 m
Carrier Gas: He
Column Diameter: 0.22 mm
Phase Thickness: 0.25 um
Data Type: Linear
RI
Program Type: Ramp
Start T: 60 C
End T: 220 C
Heat Rate: 2 K/min
End Time: 20 min
Source: Boti, J.
B.; Koukoua, G.; N'Guessan, T.Y.; Casanova, J., Chemical variability of Conyza sumatrensis and Microglossa pyrifolia from Cote d'Ivoire, Flavour Fragr. J., 22, 2007, 27-31.

2. Value: 1496 iu
Column Type:
Capillary
Column Class: Standard non-polar
Active Phase: RTX-1

Scanned by CamScanner

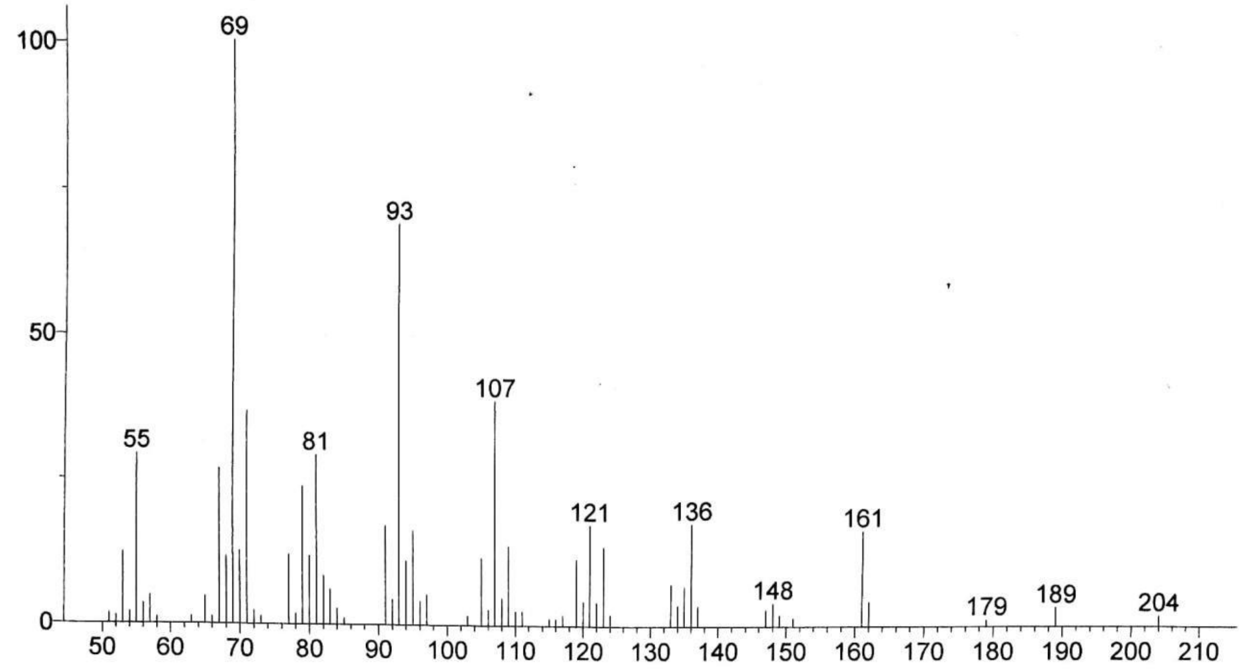

(Text File) Scan 3968 (32.792 min): 16092014C:D\data.ms

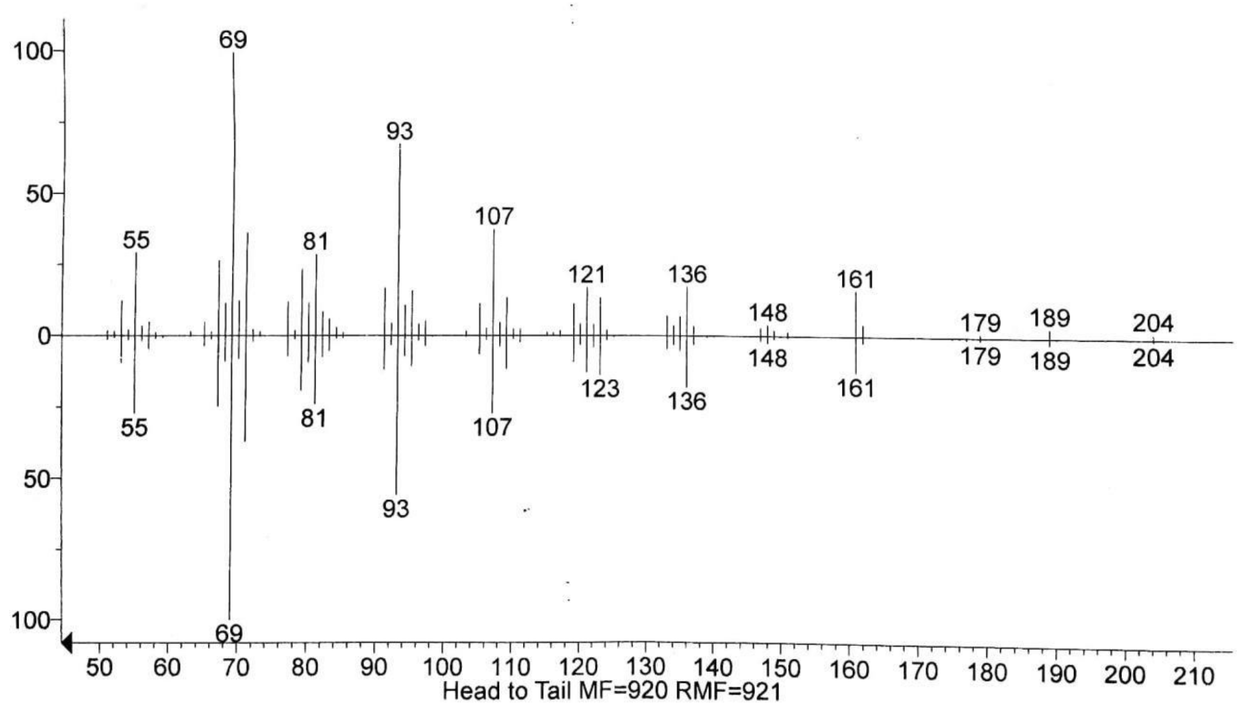

Head to Tail MF=920 RMF=921

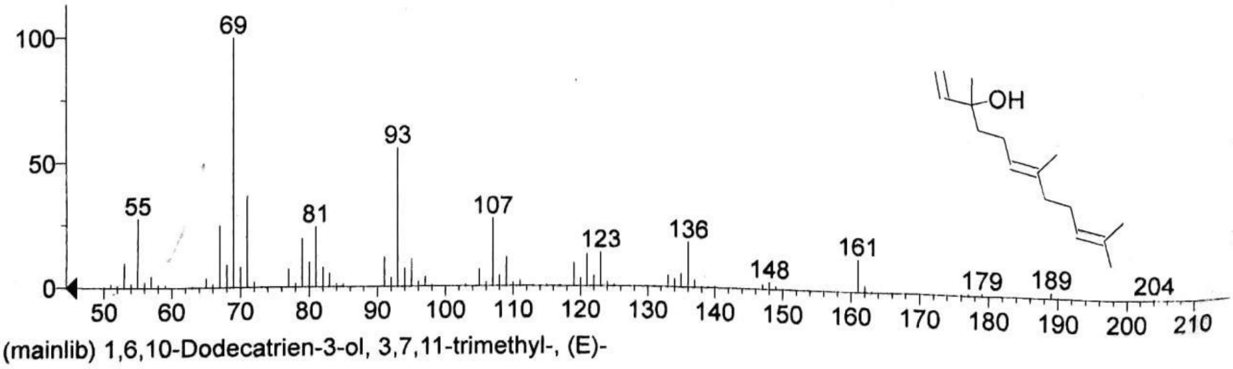

(mainlib) 1,6,10-Dodecatrien-3-ol, 3,7,11-trimethyl-, (E)-

Scanned by CamScanner

Name: 1,6,10-Dodecatrien-3-ol, 3,7,11-trimethyl-, (E)-
Formula: $C_{15}H_{26}O$
MW: 222 Exact Mass: 222.198365 CAS#: 40716-66-3 NIST#: 108468 ID#: 31602 DB: mainlib
Other DBs: EINECS
Contributor: Chuck Anderson, Aldrich Chemical Co.
Related CAS#: 2211-29-2
10 largest peaks:

69 999 |	41 586 |	93 560 |	43 410 |	71 370 |
107 275 |	55 272 |	67 249 |	81 241 |	79 192 |

Synonyms:
1.(±)-trans-Nerolidol
2.(6E)-3,7,11-Trimethyl-1,6,10-dodecatrien-3-ol #
3.(6E)-Nerolidol
4.E-Nerolidol
5.Nerolidol, E-
6.Nerolidol, trans
7.trans-Nerolidol
8.trans-3,7,11-Trimethyl-dodeca-1,6,10-trien-3-ol
9.3,7,11-Trimethyl-1,6,10-dodecatrien-3-ol, (E)-

Estimated non-polar retention index (n-alkane scale):
Value: 1564 iu
Confidence interval (Alcohols): 41(50%) 176(95%) iu

Retention index.
1. Value: 1548 iu
Column Type: Capillary
Column Class: Standard non-polar
Active Phase: BP-1
Column
Length: 50 m
Carrier Gas: He
Column Diameter: 0.22 mm
Phase Thickness: 0.25 um
Data Type: Linear
RI
Program Type: Ramp
Start T: 60 C
End T: 220 C
Heat Rate: 2 K/min
End Time: 20 min
Source: Boti, J.
B.; Koukoua, G.; N'Guessan, T.Y.; Casanova, J., Chemical variability of Conyza sumatrensis and Microglossa pyrifolia from Cote d'Ivoire, Flavour Fragr. J., 22, 2007, 27-31.

2. Value: 1555 iu
Column Type:
Capillary
Column Class: Standard non-polar
Active Phase: CP-Sil PONA GB
Column Length: 100 m
Carrier
Gas: He
Column Diameter: 0.25 mm
Phase Thickness: 0.25 um
Data Type: Linear RI
Program Type:
Ramp

Scanned by CamScanner

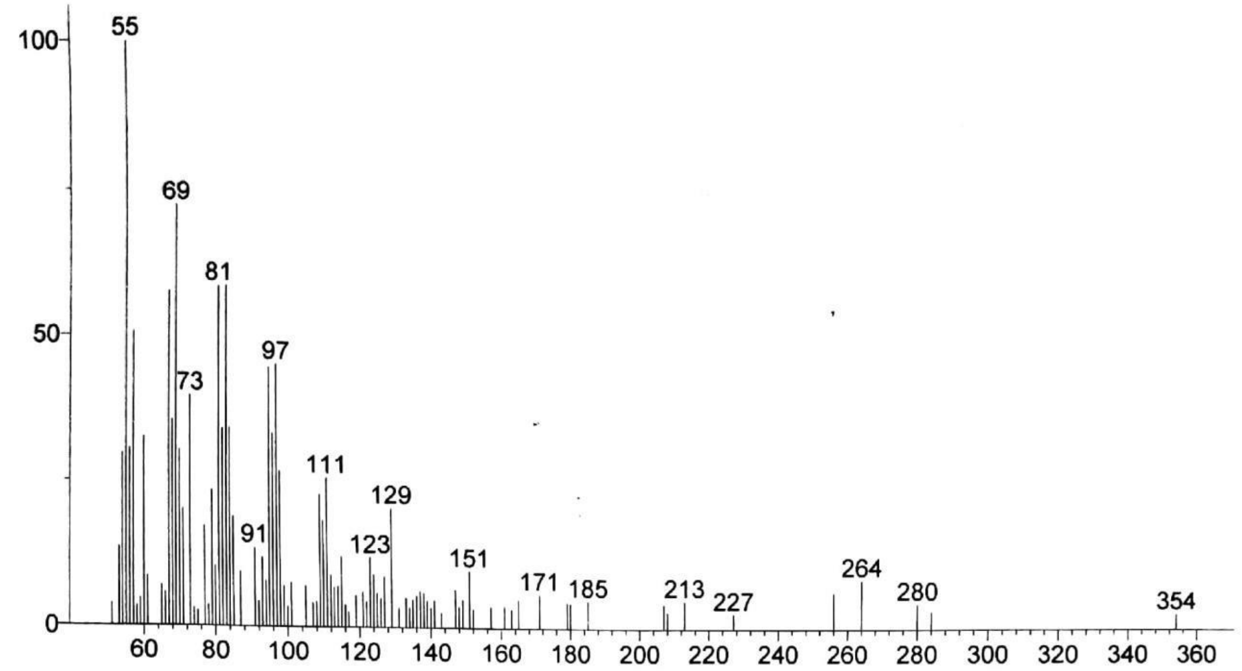

(Text File) Scan 8948 (61.287 min): 16092014C.D\data.ms

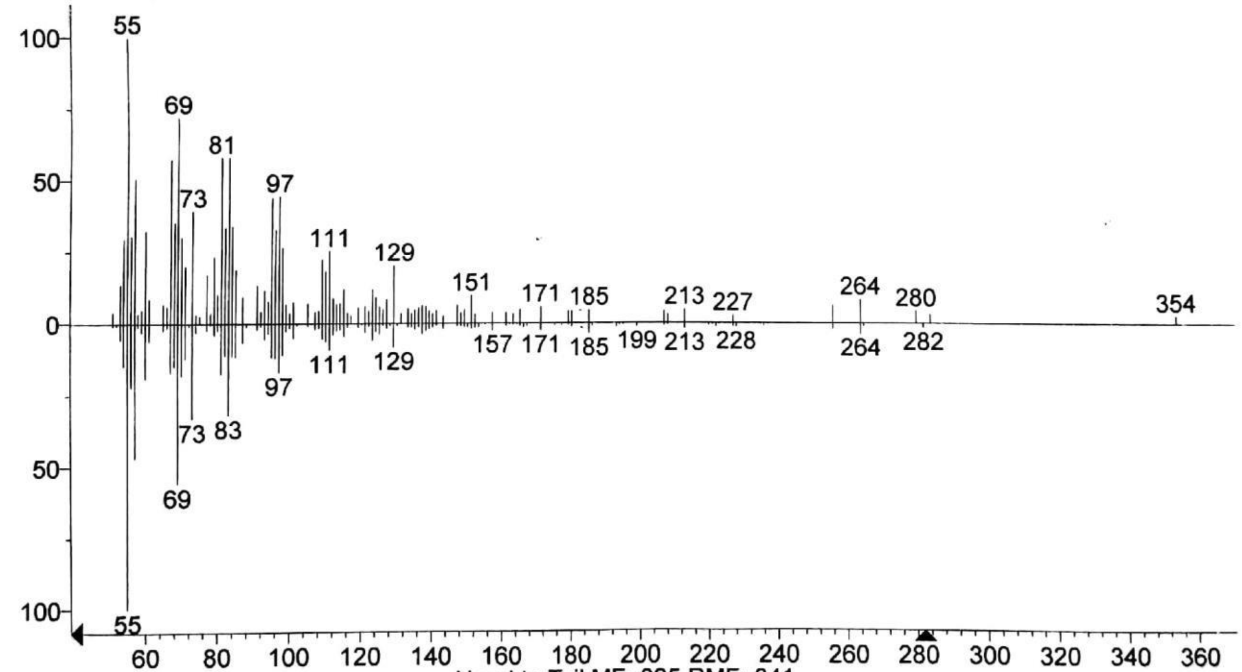

Head to Tail MF=825 RMF=841

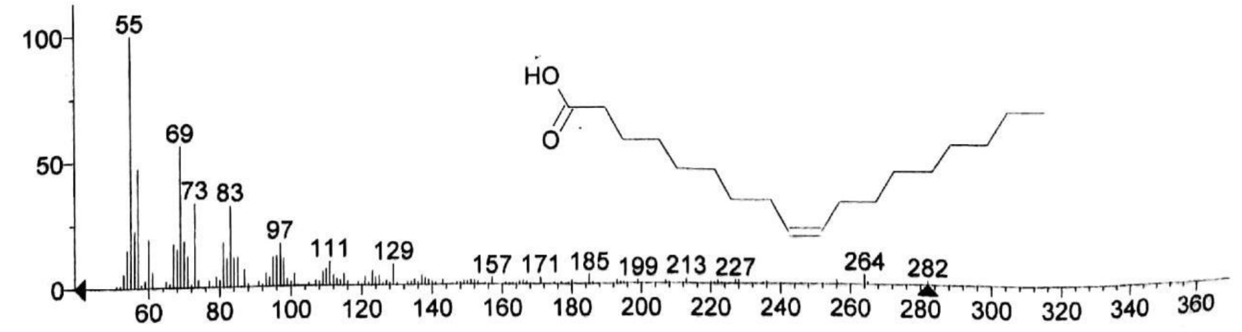

(replib) Oleic Acid

Scanned by CamScanner

Name: Oleic Acid
Formula: $C_{18}H_{34}O_2$
MW: 282 Exact Mass: 282.25588 CAS#: 112-80-1 NIST#: 154664 ID#: 4486 DB: replib
Other DBs: TSCA, RTECS, USP, HODOC, NIH, EINECS, IRDB
Contributor: Chemical Concepts
Related CAS#: 56833-51-3; 8046-01-3; 949900-16-7
10 largest peaks:

55 999 |	41 607 |	69 560 |	57 471 |	43 356 |
73 332 |	83 321 |	29 259 |	56 224 |	60 192 |

Synonyms:
1.9-Octadecenoic acid (Z)-
2..DELTA.9-cis-Oleic acid
3.cis-Oleic Acid
4.cis-9-Octadecenoic Acid
5.Emersol 211
6.Emersol 220 White Oleic Acid
7.Emersol 221 Low Titer White Oleic Acid
8.Oelsauere
9.Oleine 7503
10.Pamolyn 100
11.Vopcolene 27
12.Wecoline OO
13.Z-9-Octadecenoic acid
14.cis-Octadec-9-enoic acid
15.cis-.DELTA.9-octadecenoic acid
16.cis-.DELTA.9-Octadecenoate
17.neo-Fat 90-04
18.neo-Fat 92-04
19.Century cd fatty acid
20.Elaidoic acid
21.Emersol 210
22.Emersol 213
23.Emersol 6321
24.Glycon RO
25.Glycon WO
26.Groco 2
27.Groco 4
28.Groco 5l
29.Groco 6
30.Hy-phi 1055
31.Hy-phi 1088
32.Hy-phi 2066
33.Hy-phi 2088
34.Hy-phi 2102
35.K 52
36.L'Acide oleique
37.Metaupon
38.Tego-oleic 130
39.9-Octadecenoic acid, cis-
40.Elaic acid
41.Industrene 105
42.Industrene 205
43.Industrene 206
44.Oleinic acid
45.Pamolyn
46.Wochem no. 320
47.(Z)-9-Octadecanoic acid
48.Emersol 6313 NF

Scanned by CamScanner

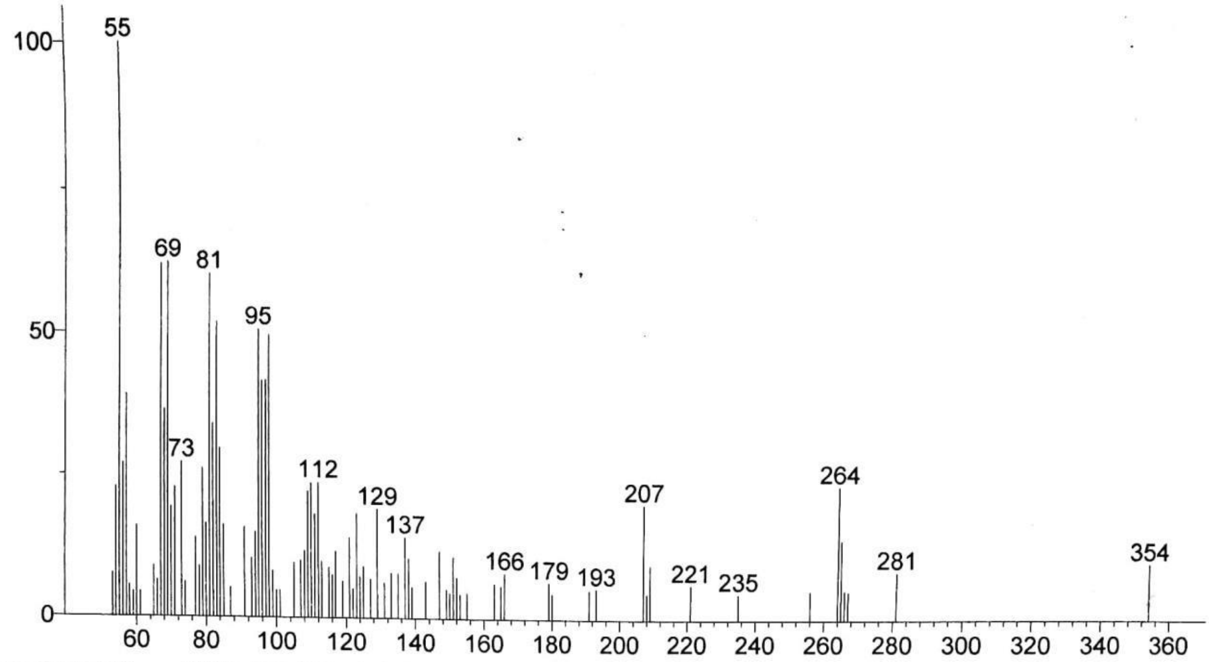

(Text File) Scan 11080 (73.486 min): 16092014C.D\data.ms

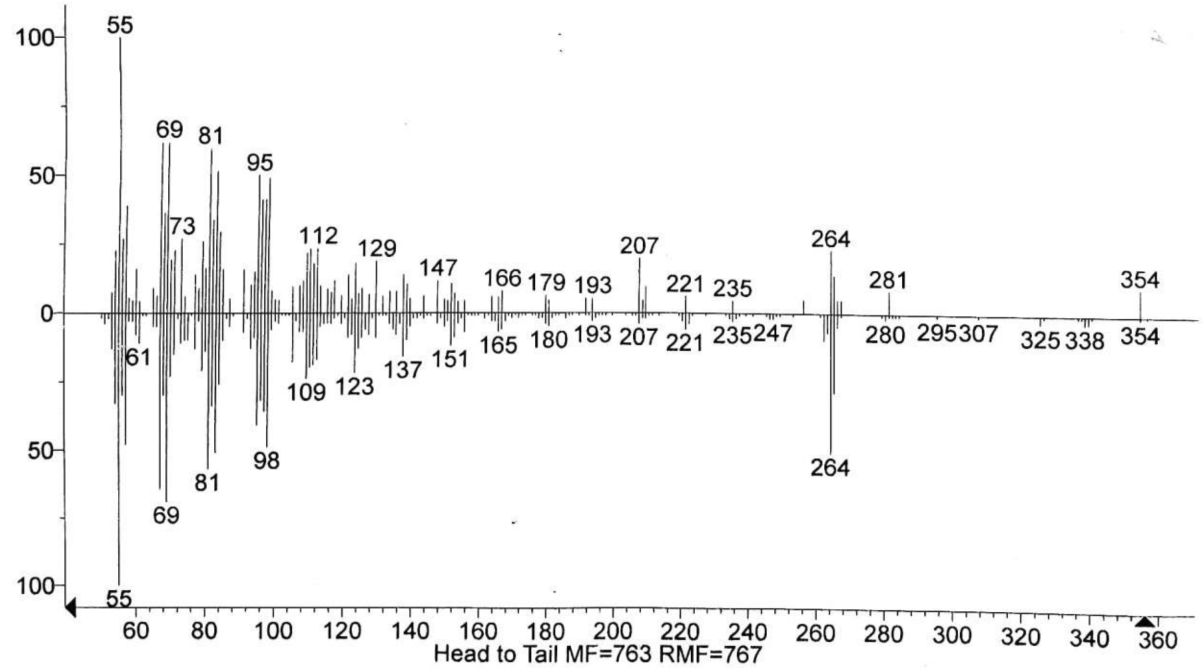

Head to Tail MF=763 RMF=767

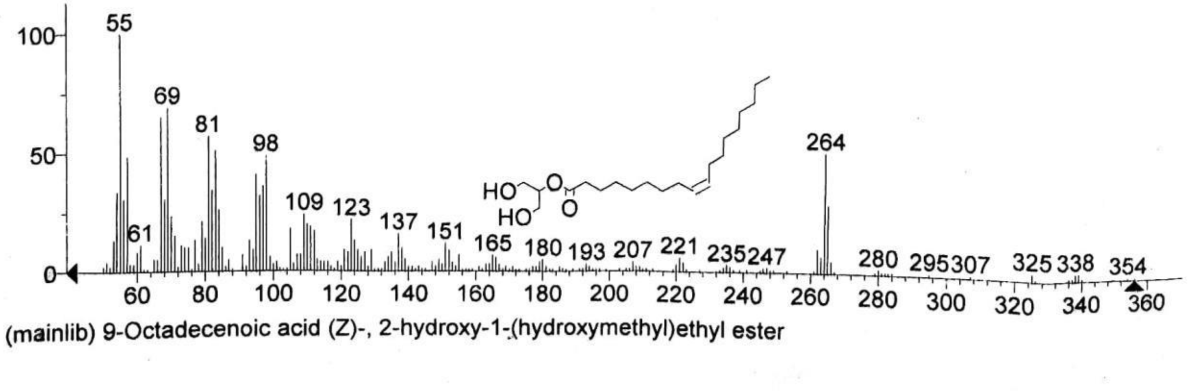

(mainlib) 9-Octadecenoic acid (Z)-, 2-hydroxy-1-(hydroxymethyl)ethyl ester

Scanned by CamScanner

Name: 9-Octadecenoic acid (Z)-, 2-hydroxy-1-(hydroxymethyl)ethyl ester
Formula: $C_{21}H_{40}O_4$
MW: 356 Exact Mass: 356.29266 CAS#: 3443-84-3 NIST#: 16061 ID#: 18035 DB: mainlib
Other DBs: None
10 largest peaks:

55 999 |	41 900 |	69 690 |	43 660 |	67 650 |
81 570 |	83 510 |	264 510 |	98 490 |	57 480 |

Synonyms:
1.Olein, 2-mono-
2.β-Monoolein
3.Glycerol 2-monooleate
4.2-Monoolein
5.2-Monooleoylglycerol
6.2-Oleoyl glycerol ether
7.2-Oleoylglycerol
8.2-Hydroxy-1-(hydroxymethyl)ethyl (9Z)-9-octadecenoate
9.β-Glyceryl monooloeate

Estimated non-polar retention index (n-alkane scale):
Value: 2705 iu
Confidence interval (Diverse functional groups): 89(50%) 382(95%) iu

Scanned by CamScanner

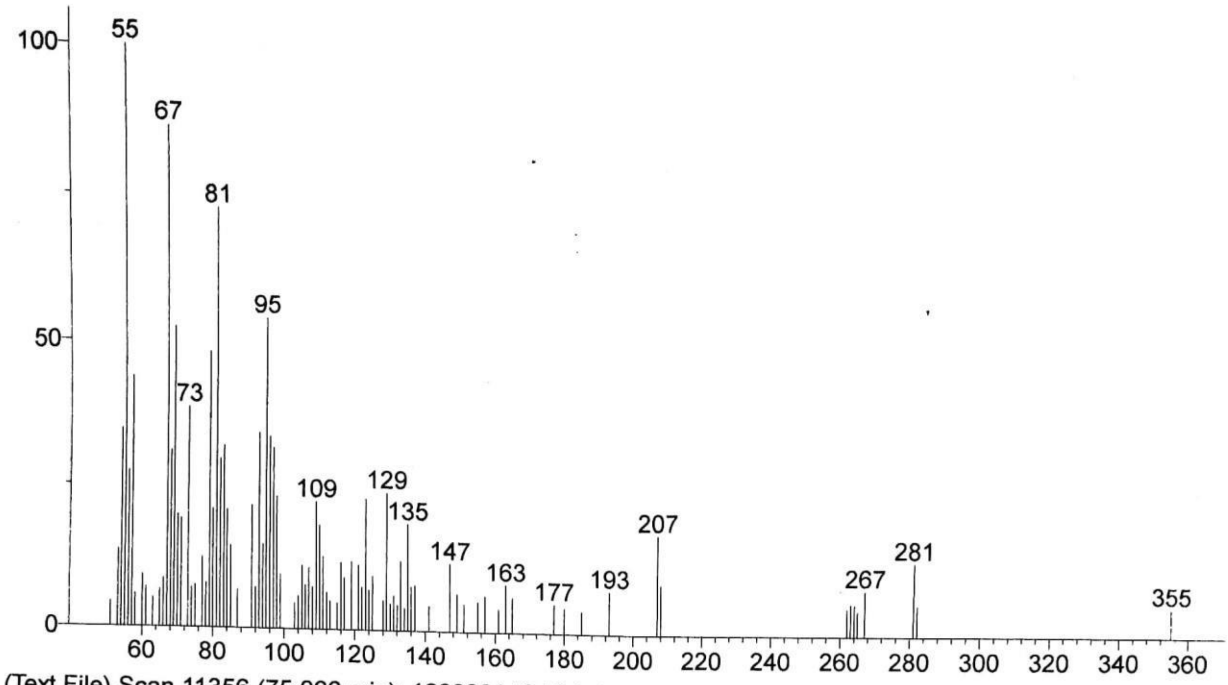

(Text File) Scan 11356 (75.066 min): 16092014C.D\data.ms

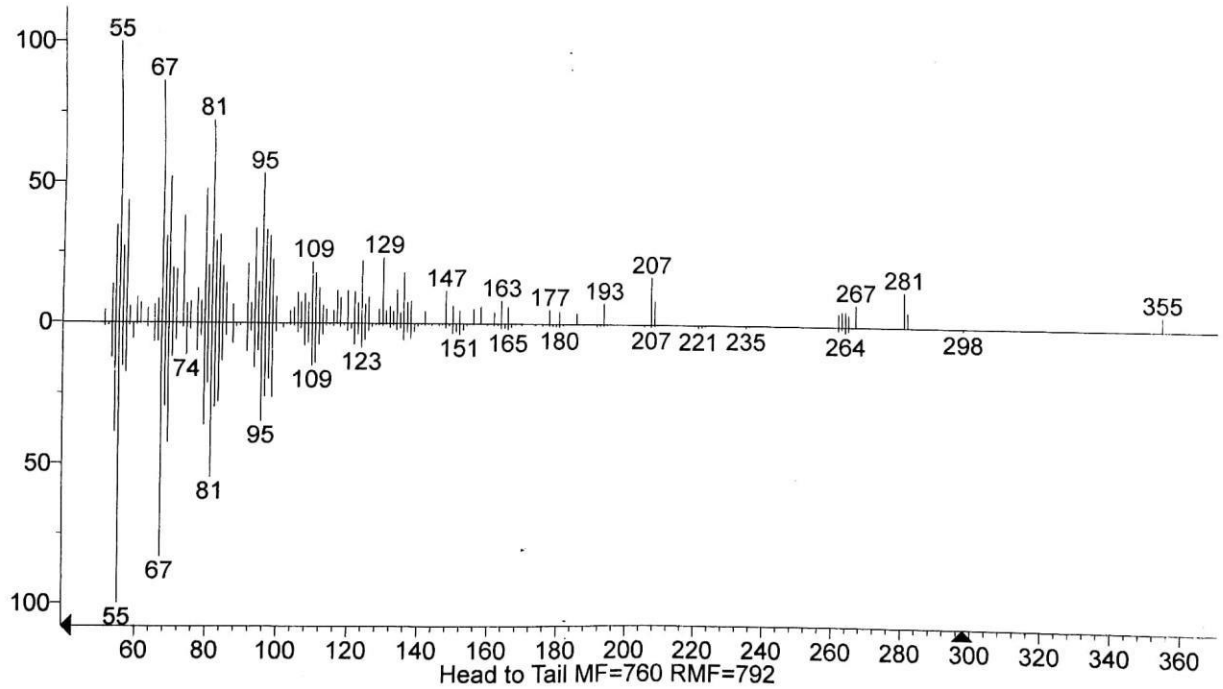

Head to Tail MF=760 RMF=792

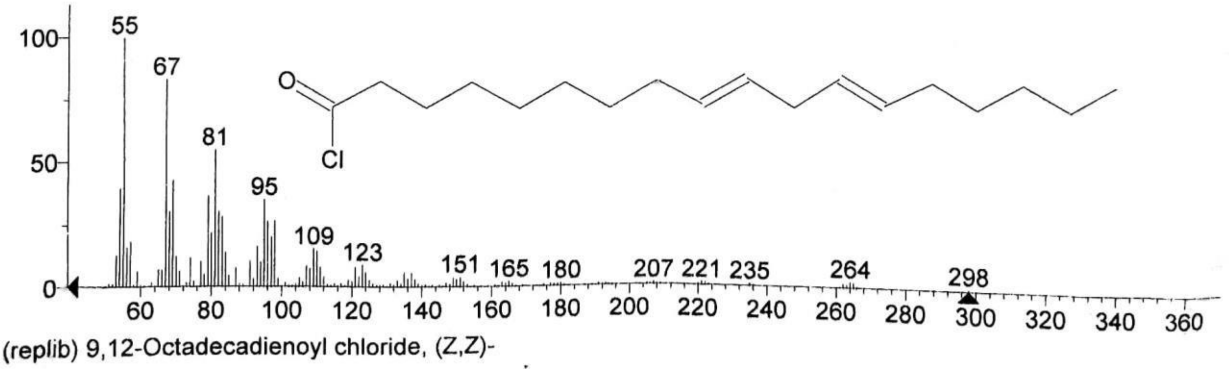

(replib) 9,12-Octadecadienoyl chloride, (Z,Z)-

Scanned by CamScanner

Name: 9,12-Octadecadienoyl chloride, (Z,Z)-
Formula: $C_{18}H_{31}ClO$
MW: 298 Exact Mass: 298.206343 CAS#: 7459-33-8 NIST#: 76312 ID#: 4686 DB: replib
Other DBs: Fine, TSCA, NIH, EINECS
Contributor: RADIAN CORP
10 largest peaks:

55 999 |	67 832 |	41 754 |	81 549 |	69 426 |
54 389 |	79 362 |	43 355 |	95 350 |	82 299 |

Synonyms:
1.Linoleoyl chloride
2.Lineoleoyl chloride
3.Linoleic acid chloride
4.(9E,12E)-9,12-Octadecadienoyl chloride #

Estimated non-polar retention index (n-alkane scale):
Value: 2139 iu
Confidence interval (Diverse functional groups): 89(50%) 382(95%) iu

Scanned by CamScanner

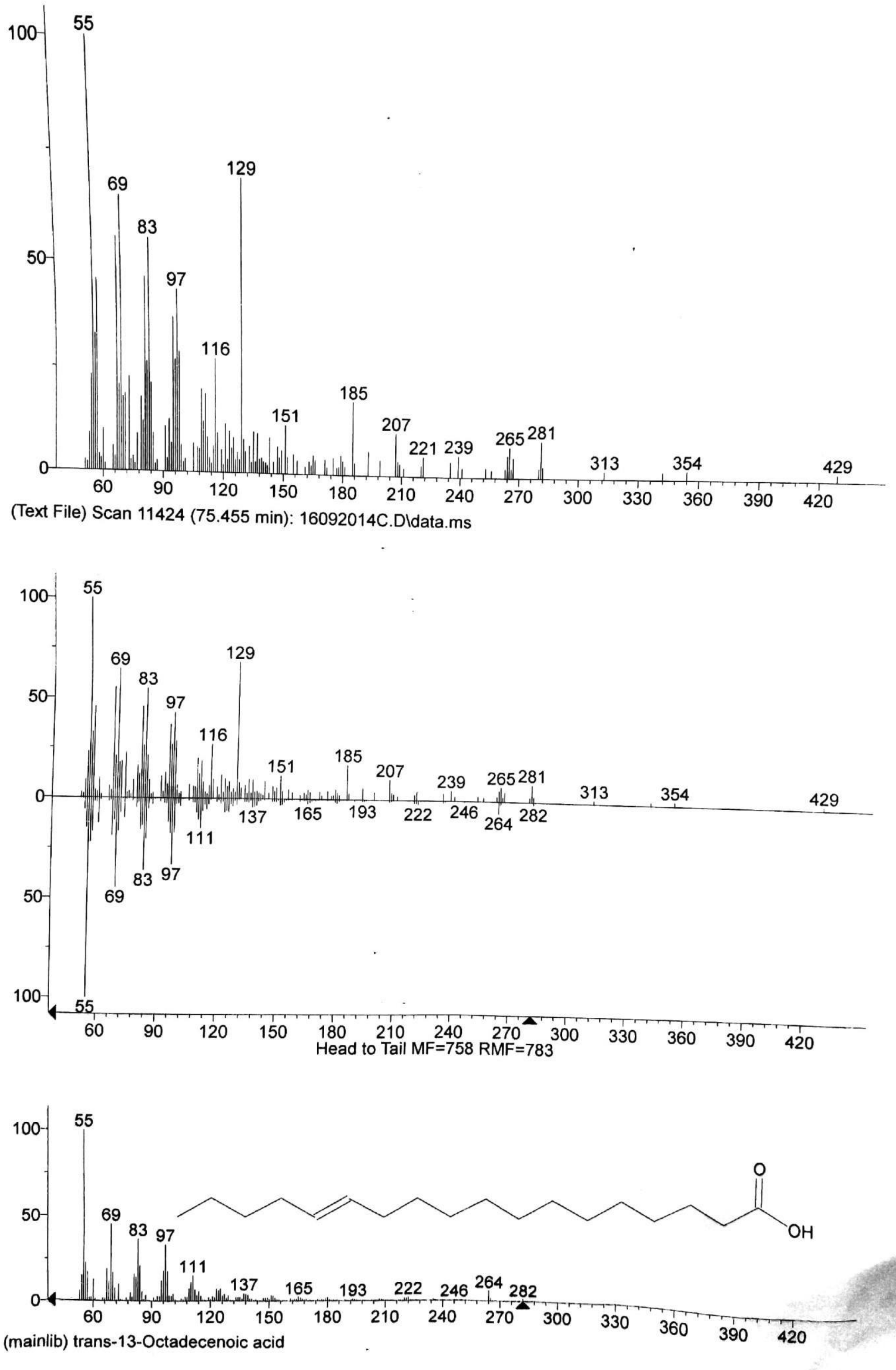

Scanned by CamScanner

Name: trans-13-Octadecenoic acid
Formula: $C_{18}H_{34}O_2$
MW: 282 Exact Mass: 282.25588 CAS#: 693-71-0 NIST#: 333615 ID#: 18062 DB: mainlib
Other DBs: None
Contributor: NIST Mass Spectrometry Data Center
10 largest peaks:

55 999 |	41 463 |	69 448 |	83 362 |	97 333 |
43 248 |	56 222 |	84 206 |	67 188 |	96 177 |

Synonyms:
no synonyms.

Estimated non-polar retention index (n-alkane scale):
Value: 2175 iu
Confidence interval (Carboxylic acids): 51(50%) 220(95%) iu

Retention index.
1. Value: 2163.6 iu
Column Type: Capillary
Column Class: Semi-standard non-polar
Active Phase: VF
-5MS
Column Length: 30 m
Carrier Gas: He
Column Diameter: 0.25 mm
Phase Thickness: 0.25 um
Data
Type: Linear RI
Program Type: Complex
Description: Multi-step temperature program; T(initial)=60C; T(final)
=270C
Source: Tret'yakov, K.V., Retention Data. NIST Mass Spectrometry Data Center., 2007.

Scanned by CamScanner

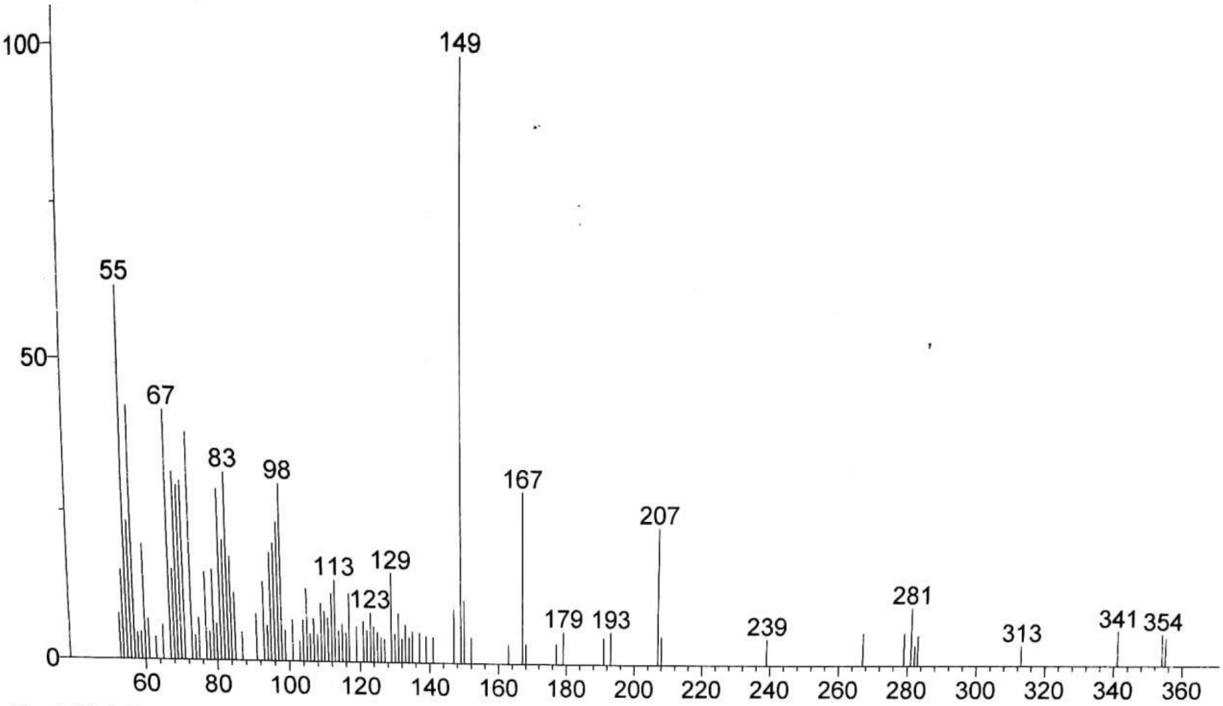

(Text File) Scan 11870 (78.007 min): 16092014C.D\data.ms

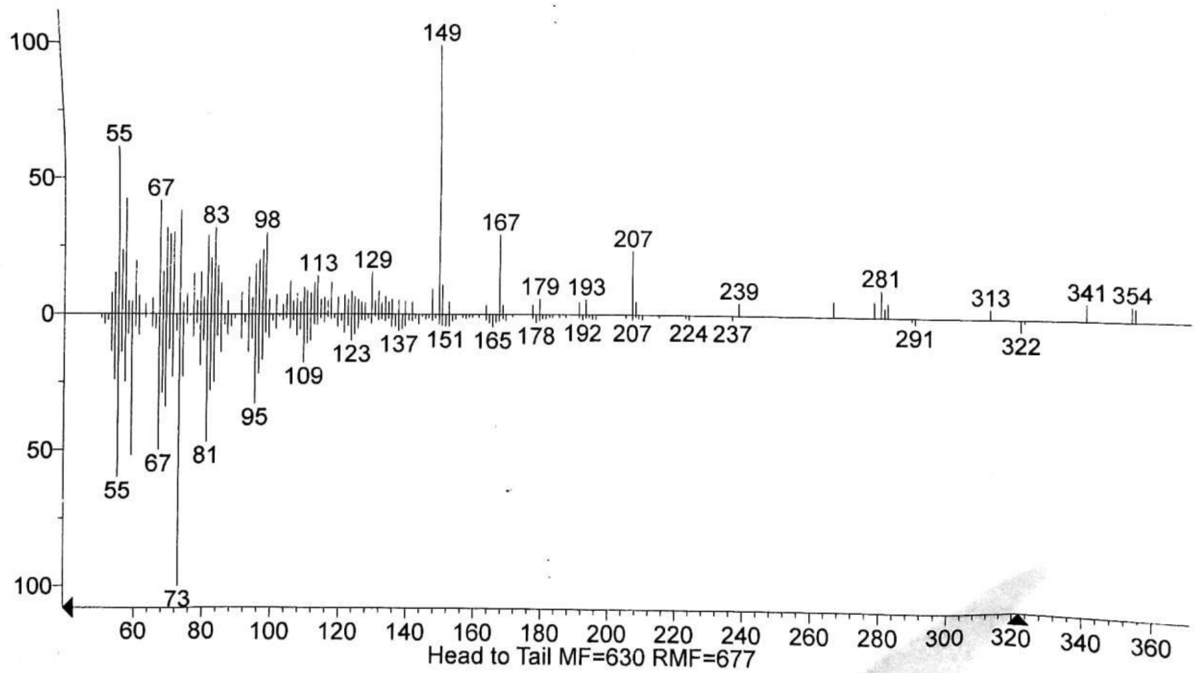

Head to Tail MF=630 RMF=677

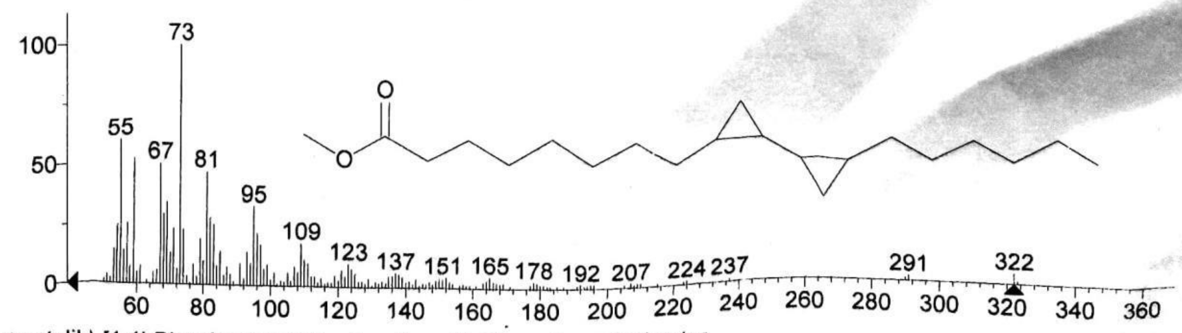

(mainlib) [1,1'-Bicyclopropyl]-2-octanoic acid, 2'-hexyl-, methyl ester

Scanned by CamScanner
